# Study on the Chemical Composition and Multidrug Resistance Reversal Activity of *Euphorbia uralensis* (Euphorbiaceae)

**DOI:** 10.3390/ijms26010412

**Published:** 2025-01-06

**Authors:** Yina Ding, Yuhao Liu, Qianru Dang, Zubair Akram, Anam Arshad, Haochan Zhu, Jianxiang Zhang, Bo Han, Chimengul Turghun

**Affiliations:** 1 Key Laboratory of Xinjiang Phytomedicine Resource and Uilization, Ministry of Education, Shihezi 832002, China; 15693241553@163.com (Y.D.); liuyuhao@stu.shzu.edu.cn (Y.L.); 20211015197@stu.shzu.edu.cn (Q.D.); zubair.a.sidhu@gmail.com (Z.A.); anamarshad9293@gmail.com (A.A.); zhuhaochan@stu.shzu.edu.cn (H.Z.); 2 School of Pharmacy, Shihezi University, Shihezi 832002, China; 20221015169@stu.shzu.edu.cn; 3 School of Chemistry and Chemical Engineering, Shihezi University, Shihezi 832002, China

**Keywords:** activity-oriented methods, monomer compound, MDR reversal activity, MDR reversal mechanism

## Abstract

*Euphorbia uralensis* belongs to the family Euphorbiaceae and is widely distributed in northern Xinjiang, making it a characteristic plant of the region in Xinjiang, China. The chemical composition and biological activity of *Euphorbia uralensis* have not yet been reported, although certain compounds isolated from *Euphorbia* plants in Xinjiang, China, have demonstrated exceptional multidrug resistance (MDR) reversal. This study aims to investigate the chemical components present in *Euphorbia uralensis* with the potential to reverse MDR. The aerial parts of *Euphorbia uralensis* were extracted using organic solvents of varying polarities, resulting in dichloromethane (Fr-E) and petroleum ether (Fr-S) fractions, which exhibited greater MDR reversal activity than the other fractions. The chemical constituents of the Fr-S fraction were analyzed using GC-MS. The chemical components of the Fr-E fraction were isolated and purified using column chromatography. The most effective compounds with MDR reversal activity were screened out, and the mechanism was investigated using molecular docking, molecular dynamics simulations, Western blotting, and rhodamine 123 staining. GC-MS analysis showed that the Fr-S fraction was rich in triterpenes, fatty acids, phenols, and long-chain alkanes, all of which were identified for the first time in *Euphorbia uralensis*. Among these, palmitic acid was present at a content level of 15.86%. This study notably unveils the discovery of a new compound and 16 previously recorded compounds for the first time in this plant, with the main types identified as steroids, sesquiterpenes, and flavonoids. The isolated compounds were tested for cytotoxicity and MDR reversal activity. The new compounds Euphouralosides A, pubinernoid A, naringenin, and punigratine showed good MDR reversal activity against MCF-7 and MCF-7/ADR cell lines. Punigratine was the most active compound. Moreover, punigratine could stably bind to the ABCB1 protein. Western blot analysis revealed that punigratine did not affect the expression of the ABCB1 protein in cells (*p* > 0.05). However, following treatment with punigratine (0.16 μM), there was a significant increase the intracellular accumulation of Rh123 in MCF-7/ADR cells (*p* < 0.05). These findings suggest that punigratine can inhibit the efflux of the ABCB1 protein, thereby overcoming MDR in tumors. This study provides a foundation for further research on the biological activity and medicinal potential of *Euphorbia uralensis*.

## 1. Introduction

Over the past decade, significant progress has been made in early screening methods, diagnostic techniques, and treatment strategies, leading to a steady reduction in cancer mortality rates. Nevertheless, despite these notable advancements, cancer remains one of the leading causes of death across numerous nations [1]. Chemotherapy remains the primary therapeutic modality for tumors; however, its efficacy is often compromised by the frequent occurrence of multidrug resistant (MDR) [2]. MDR is defined as the phenomenon when cancer cells develop resistance to multiple drugs, leading to cross-resistance [3,4] and significantly diminishing the efficacy of chemotherapy [5]. Therefore, there is an urgent need to develop effective and low-toxicity MDR reversal agents to reduce drug resistance and tumor cell metastasis, thereby facilitating their effective therapeutic intervention.

The use of natural products to develop MDR reversal agents represents a prominent strategy in current cancer treatment [6]. Consequently, the remarkable MDR reversal activity [7,8] exhibited by flavonoids, terpenoids, steroids, and other compounds has attracted significant attention from researchers [9].

Xinjiang province in China is renowned for its diverse variety of *Euphorbia* plants, which exhibit extensive biological activity and have a long-standing history of medicinal use in treating edema, ascites, and myalgia [10,11]. Numerous researchers have systematically isolated and purified chemical constituents from *Euphorbia* plants in Xinjiang [12,13,14,15,16], with some compounds exhibiting promising MDR reversal activity [17]. *Euphorbia uralensis* Fisch. Ex Link. (1822) is classified in the Euphorbiaceae family and the *Euphorbia* genus and is an indigenous species in Xinjiang, China. However, the chemical composition and biological activity of *Euphorbia uralensis* have not yet been reported. We hypothesize that *Euphorbia uralensis* may also contain MDR reversal compounds similar to those found in other *Euphorbia species* in Xinjiang. Accordingly, the active components were isolated using an activity-guided methodology in this study. The most potent compound was then selected to investigate its mechanism of action in reversing MDR, with the aim of discovering highly effective, low-toxicity agents for cancer treatment. This study also aims to provide a foundation for developing distinctive medicinal resources in Xinjiang. This work may also contribute to the isolation of novel natural compounds that act as potent inhibitors for cancer treatment.

## 2. Results and Discussion

### 2.1. Cytotoxicity Results of Total Extracts, Extraction Fractions

To determine the cytotoxicity, the maximum non-toxic concentrations of the extracts from different sites were determined using the Cell Counting Kit-8 (CCK-8) method. MCF-7 and MCF-7/ADR cells were treated with total extracts and fractions in the concentration range of 0–100 μg/mL, using doxorubicin (DOX, 0–50 μM) as a drug-resistant inducer, and the results (Table 1) showed that the maximum non-toxic concentrations of the extracts from each site and verapamil, a positive control, were as follows: verapamil (0–55 μM); total extracts Fr-Z (0–12.5 μg/mL); petroleum ether Fr-S fraction (0–6.25 μg/mL), dichloromethane Fr-E fraction (0–12.5 μg/mL), ethyl acetate Fr-Y fraction (0–12.5 μg/mL), n-butanol Fr-J fraction (0–25 μg/mL). Neither the total extract nor the fractions within these concentrations were cytotoxic to MCF-7 and MCF-/ADR cells.

**Figure 1:**
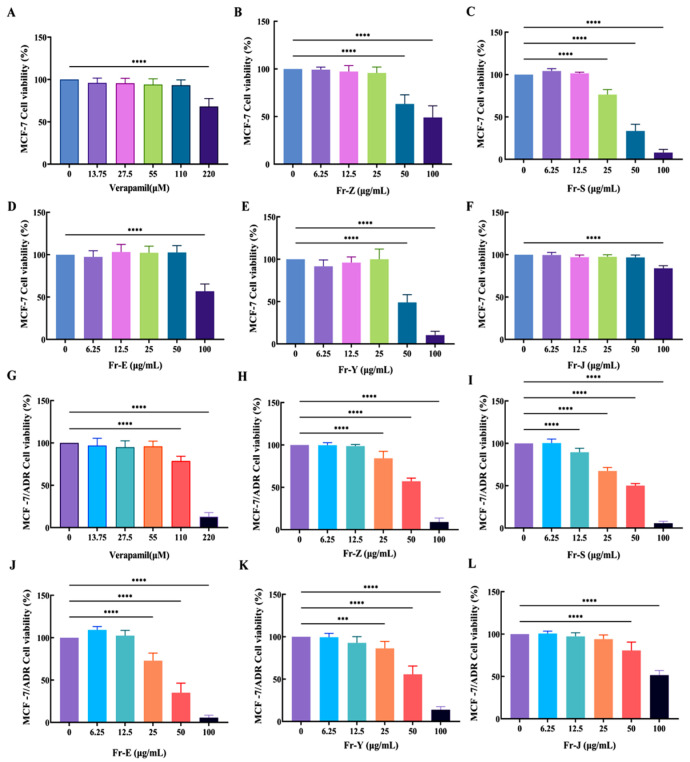
Cytotoxicity of extracts and fractions on MCF-7 cells (**A**–**F**) and MCF-7/ADR cells (**G**–**L**) (mean ± SD, n = 3). Note: Cell Viability (%) = [(OD Drug − OD Blank)/(OD control − OD blank)] × 100. All data were compared with a blank control group (0 μg/mL of drug concentration) *** *p* < 0.001; **** *p* < 0.0001.

Notably, the Fr-E fraction within the concentration range of 25–50 μg/mL and the Fr-S fraction at concentrations of 6.25–12.5 μg/mL demonstrated significant cytotoxicity towards MCF-7/ADR cells while exhibiting no cytotoxicity towards MCF-7 cells. This suggests that these fractions may possess anti-tumor properties and potential MDR reversal activity (Figure 1).

### 2.2. MDR Reversal Activity Evaluation of Total Extracts and Fractions

Based on the cytotoxicity results, the maximum non-toxic concentrations of the total extracts and fractions were selected as the administered concentrations for the MDR reversal activity assay. The IC_50_ values of the five extracts and fractions on the MCF-7/ADR cells treated with DOX (0–50 μM) were determined using the CCK-8 method, and the RF values for MDR reversal were calculated. The results demonstrated that treatment with the extracts significantly reduced the IC_50_ values of Fr-S (30.57 ± 5.48 μM) and Fr-E (21.99 ± 1.94 μM) compared to the control DOX group (65.62 ± 5.54 μM) (* *p* < 0.05). However, there was no significant change in the IC_50_ values of the Fr-J, Fr-Z, and Fr-Y extracts compared to the control DOX. In addition, the RF values of Fr-E and Fr-S were 3.0 and 2.1, respectively, indicating that they had strong MDR reversal activities, with the strongest activity at the Fr-E site (Figure S2, Table 1).

The results above demonstrate that both the Fr-E and Fr-S extracts can enhance the sensitivity of MCF-7/ADR cells to DOX. Moreover, the cytotoxicity of the Fr-E extract is comparatively lower while it exhibits a stronger ability to reverse MDR compared to the Fr-S extract. Therefore, it is recommended that further purification research be focused specifically on targeting the effective properties of Fr-E. The flow fraction Fr-E was separated using forward silica gel chromatography. The solvent mixture of petroleum ether and ethyl acetate was eluted using a series of concentration gradients of 100:1, 50:1, 25:1, 10:1, 5:1, and 0:100. The individual flow fractions were detected using thin-layer chromatography (TLC), and similar flow fractions were combined to obtain three flow fractions: Fr-E-1, Fr-E-2, and Fr-E-3.

The Fr-E-1, Fr-E-2, and Fr-E-3 fractions were evaluated for their efficacy in reversing MDR. The IC_50_ value of the Fr-E-3 fraction was determined to be 25.60 ± 12.50 μM, and the RF value was 2.7 (* *p* < 0.05 vs. the control group). Additionally, other fractions, namely Fr-E-1 (IC_50_ = 31.86 ± 10.61; RF = 2.2) and Fr-E-2 (IC_50_ = 33.96 ± 13.36; RF = 2.0), also exhibited some degree of MDR reversal activity on MCF-7/ADR cells (Table 1). Therefore, the Fr-E-3 fraction demonstrated notable activity in reversing MDR reversal and exhibited dose-dependent responses with increasing DOX concentrations. The extraction of medicinal materials has been reported to demonstrate potential activity in reversing MDR. For example, Stellera chamaejasme extract significantly inhibits the proliferation of MDA-MB-231/MDR cells in triple-negative breast cancer [18,19]. Additionally, numerous studies have documented the purification and isolation of compounds from herbs that exhibit potent anti-drug resistance activity, such as *Euphorbia sororiai* [20], *Euphorbia glomerulans* [21], and *Euphorbia macrorrhiza* [22], which possess diverse activities in reversing drug resistance [23]. Consequently, the Fr-E-3 fraction was further separated and purified based on the activity results.

### 2.3. Research on the Chemical Composition of Fr-E-3 Fractions from Euphorbia uralensis

**Figure 2:**
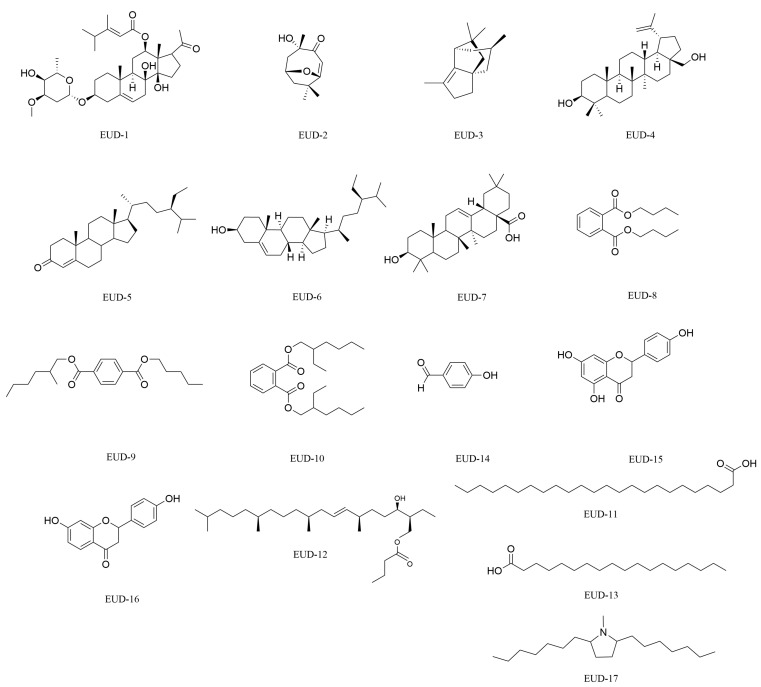
Structures of the isolated monomer compounds from *Euphorbia uralensis*.

In this study, 17 compounds were isolated from the Fr-E-3 fraction of *Euphorbia uralensis,* including 1 new compound and 16 known compounds. The structural identification was performed using one-dimensional and two-dimensional nuclear magnetic resonance (^1^H-NMR, ^13^C-NMR) and Fourier-transform infrared spectroscopy. The results revealed that the compounds primarily consisted of triterpenes, steroids, flavonoids, alkaloids, aromatic lipids, aldehydes, and long-chain fatty acids. Furthermore, all individual compounds were successfully isolated from *Euphorbia uralensis* for the first time. The structural formulas of the compounds are shown in Figure 2, and the names and types of compounds are listed in Table S1.

### 2.4. Structural Analysis of the New Compound

EUD-1 is a white amorphous powder. Based on the HRESI(+) MS data (*m*/*z* 641.3672 [M+Na]^+^, theoretical value C_35_H_54_NaO_9_, 641.3660), the molecular formula was determined to be C_35_H_54_O_9_. Following TLC analysis, brownish–black spots were visible under 254 nm. According to the IR spectrum analysis, the compound contains the following functional groups and structural fragments: stretching vibration of O-H (3317 cm^−1^), stretching vibration of C=O (1711, 1697 cm^−1^), skeletal vibration of C_6_H_6_ (benzene ring) at 1460 cm^−1^, ester group absorption peak at 1227 cm^−1^, and asymmetric stretching vibration of the ether bond (C-O-C) at 1090 cm^−1^.

The ^1^H-NMR and ^13^C-NMR (chloroform-d, 400 Hz) spectra show a set of double bond signals in the high-field region: *δ*_C_ 139.3 (C-5), *δ*_C_ 119.0 (C-6), *δ*_H_ 5.37 (d, J = 4.8 Hz, 1H). The following signals were observed for an acetyl fragment: *δ*_C_ 217.8 (C-20), *δ*_C_ 33.1 (C-21), *δ*_H_ 2.21 (s, 3H). Two hydroxyl signals were observed: *δ*_H_ 5.70 (s, 1H) and *δ*_H_ 5.21 (brs, 1H), as well as a set of ring hydrogen protons from a double oxygen-linked ring: *δ*_C_ 95.6 (C-1″), *δ*_H_ 4.78 (dd, J = 9.6, 1.6, 1H). Two angular methyl signals were observed: *δ*_C_ 121 (C-18) and *δ*_H_ 1.19 (s, 3H), *δ*_C_ 18.3 (C-19) and *δ*_H_ 1.18 (s, 3H), and a methylene-linked methyl signal was also observed: *δ*_C_ 57.4 (C-7″), *δ*_H_ 3.43 (s, 3H). Additionally, there were three methyl signals, including one single methyl peak and two double methyl peaks: *δ*_C_ 21.0 (C-5′), *δ*_H_ 1.08 (d, J = 6.8, 3H), *δ*_C_ 21.0 (C-6′), *δ*_H_ 1.08 (d, J = 6.8, 3H), *δ*_C_ 16.6 (C-7′), and *δ*_H_ 2.17 (s, 3H). There were also eight methylene signals: *δ*_C_ 39.1 (C-1), *δ*_H_ 1.85 (m, 2H), *δ*_C_ 29.2 (C-2), *δ*_H_ 1.91 (m,1H), *δ*_H_ 1.63 (m, 1H), *δ*_C_ 35.7 (C-4), *δ*_H_ 2.12 (m, 2H), *δ*_C_ 38.8 (C-7), *δ*_H_ 2.32 (m, 2H), *δ*_C_ 37.1 (C-15), *δ*_H_ 1.90 (m, 1H), *δ*_H_ 2.06 (m, 1H), *δ*_C_ 24.8 (C-16), *δ*_H_ 1.83 (m, 2H), *δ*_C_ 39.2 (C-2″), *δ*_H_ 2.19 (m, 1H), *δ*_H_ 1.58 (m, 1H). Meanwhile, the signals of *δ*_C_ 95.6 (C-1″), 39.2 (C-2″), 77.6 (C-3″), 72.6 (C-4″), 70.9 (C-5″), and 18.4 (C-6″), combined with the terminal proton signal of C-1″: *δ*_H_ 4.76 (dd, J = 9.6, 1H), suggested that the compound contained a substituted structure of *β*-pyran ring. In the ^13^C-NMR spectrum, the glycosylation shifts of C-2 (−2.2 ppm), C-3 (+6.1 ppm), and C-4 (−4.0 ppm) indicated that the sugar chain was attached to C-3 of the glycoside element.

Based on the ^1^H-^1^H COSY spectrum, four structural fragments were inferred (H_2_-1 to H_2_-2, H-3, H_2_-4; H-6 to H_2_-7; H-9 to H_2_-11, H-12; H_2_-15 to H_2_-16, H-17). The planar structure of the A ring was deduced by combining the relevant signals from H_3_-19 to C-1, C-5, C-9, and C-10 in the HMBC spectrum. The planar structure of the A ring was deduced. The planar structures of the B ring were deduced by combining the relevant signals from H-6 to C-5, H_2_-7 to C-8, C-9, and C-14 in the HMBC spectrum. The planar structures of the C and D rings were deduced by combining the relevant signals from H_3_-18 to C-12, C-13, C-14, and C-17 in the HMBC spectrum. The acetyl carbon was directly connected to C-17 of the D ring, as inferred from the relevant signals from H_3_-21 to C-20 and C-17 in the HMBC spectrum.

In the ^1^H-^1^H COSY spectrum, the correlation between H-4′ and H_3_-5′, H_3_-6′ simultaneously indicated that the structure contained an (E)-3,4-dimethyl-pentenoic acid structural fragment. This was further supported by the HMBC spectrum, which showed the correlation between H_3_-7′ and C-2′, C-3′, and C-4′, as well as between H-2′ and C-1′, and C-3′. The oxygen atom of fragment 1′ was directly connected to C-12 of the C ring, as inferred from the HMBC correlation signal from H-12 to C-1′.

In the ^1^H-^1^H COSY spectrum, starting from H-1″, sequential signals for H-2″, H-3″, H-4″, H-5″, and H-6″ were observed in sequence. Based on the correlation from H-7″ to C-3″ and H-5″ to C-1″ in the HMBC spectrum, it was inferred that the compound contained a sugar ring E. The relevant signal from H-3 to C-1″ in the HMBC spectrum confirmed that the oxygen atom at the 1″ position of sugar ring E was directly connected to C-3 of ring A.

**Figure 3:**
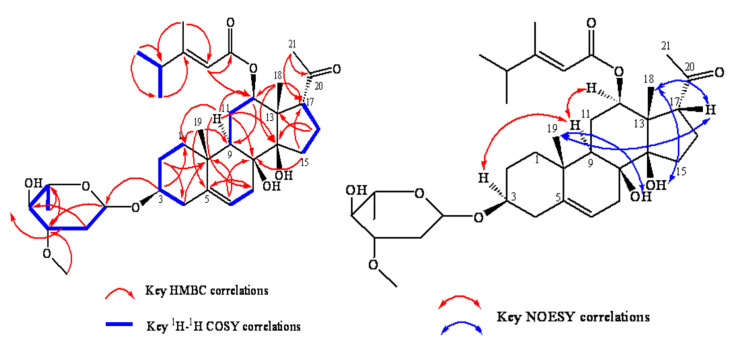
The main NOESY, ^1^H-^1^H COSY, and HMBC relationships of the new compound EUD-1.

In the NOESY spectrum, H-9 was assigned to the *α*-configuration, with correlated signals between H-3 and H-12 and H-9, indicating that both H-3 and H-12 were in the *α*-configuration. Since NOESY correlation signals were found between H_3_-18, H_3_-19, and H-17, and no correlation signals were observed between H_3_-18, H_3_-19, H-3, H-9, and H-12, it was suggested that H-17, H_3_-18, and H_3_-19 were all in the *β*-configuration. Correlation signals were also observed between 8-OH and H_3_-19 and between 14-OH and H_3_-18, indicating that 8-OH and 14-OH are on the same side of the steroid nucleus as H_3_-19 and H_3_-18 in the *β*-configuration. After comparing the data with those in the literature and consulting the SciFinder database (SciFinder^n^), the compound EUD-1 was identified as a new C_21_ steroidal glycoside analogue containing a surachidonin-3-O-*β*-D-magnetoside substitution. The NOESY, ^1^H-^1^H COSY, and HMBC relationships of EUD-1 are shown in Figure 3, and the spectral data of ^1^H-NMR and ^13^C-NMR are shown in Table 2. The spectral data are shown in Supplementary Figures S4–S8. We named this new compound Euphouralosides A.

### 2.5. Structural Analysis of Known Compounds

(1) EUD-2: White amorphous powder with the molecular formula C_11_H_16_O_3_; *m*/*z*: 196 [M]+. After TLC detection, dark spots were observed at 254 nm. The spectral details are provided in Table S2, and the spectral diagram of the compound is shown in Figure S9. After comparison with the literature [24], the compound was identified as pubinernoid A.

(2) EUD-3: White powder, molecular formula C_15_H_24_; *m*/*z*: 204 [M]+. After TLC detection, dark spots were observed under 254 nm. The spectral information is shown in Table S3, and the spectral details are shown in Figure S10. After comparison with the literature [25], the compound was identified as ginsinsene.

(3) EUD-4: White solid, molecular formula C_30_H_50_O_2_; *m*/*z*: 442 [M]+. It was readily soluble in petroleum ether and dichloromethane, with a melting point of 256–257 °C. ^13^C-NMR (chloroform-d, 100 Hz) spectral data indicated a total of 30 carbon atoms, suggesting that the compound has a triterpene parent nucleus structure. The spectral details are shown in Table S4, and the spectral diagram of the compound is shown in Figure S11. After comparing with the literature [26], the compound was identified as Betulin.

(4) EUD-5: White powder, molecular formula C_29_H_48_O; *m*/*z*: 412 [M]+. It was readily soluble in organic solvents such as petroleum ether and dichloromethane. The spectral details are shown in Table S5, and the spectral diagram is shown in Figure S12. After comparison with the reference literature [27], the compound was identified as (24R)-24-stigmast-4-en-3-one.

(5) EUD-6: White needle-like crystal, molecular formula C_29_H_50_O; *m*/*z*: 414 [M]+. It was readily soluble in chloroform, slightly soluble in ethanol or acetone, and insoluble in water. The spectral details are provided in Table S6, and the spectral diagram is shown in Figure S13. After comparison with the literature [28], the compound was identified as β-sitosterol.

(6) EUD-7: White amorphous powder with the molecular formula C_30_H_48_O_3_; *m*/*z*: 456 [M]+. It was readily soluble in petroleum ether and dichloromethane. The reaction of acetic anhydride with concentrated sulfuric acid yielded purple–red spots, suggesting that the compound might be a terpene or a sterol. The spectral details are provided in Table S7, and the compound’s spectral diagram is shown in Figure S14. After comparing with the literature [29], the compound was identified as oleanolic acid.

(7) EUD-8: A pale-yellow oil with the molecular formula C_16_H_22_O_4_; *m*/*z*: 278 [M]+. After TLC development, distinct fluorescent spots were observed at 254 nm. It was insoluble in water and exhibited low solubility and volatility. The spectral details are shown in Table S8, and the spectrum is provided in Figure S15. After comparing with the literature [30], the compound was identified as dibutyl phthalate.

(8) EUD-9: A pale-yellow oil with the molecular formula C_20_H_30_O_4_; *m*/*z*: 335 [M+H]+. After TLC development, distinct fluorescent spots were observed at 254 nm. It was readily soluble in solvents such as petroleum ether and dichloromethane but insoluble in water. The spectral details are provided in Table S9, and the spectrum is shown in Figure S16. After a comprehensive comparison with the literature [31], the compound was identified as auriculatum A.

(9) EUD-10: A pale yellow oil with the molecular formula C_24_H_38_O_4_; *m*/*z*: 391 [M+H]+. After TLC development, it exhibited distinct fluorescent spots at 254 nm. It was readily soluble in solvents such as petroleum ether, dichloromethane, and acetone and miscible with most hydrocarbons but insoluble in water. The spectral details are provided in Table S10, and the spectrum is shown in Figure S17. After comparing with the literature [32], the compound was identified as di-(2-ethyl) hexyl phthalate.

(10) EUD-11: A pale-yellow oily substance with the molecular formula C_24_H_48_O_2_; *m*/*z*: 368 [M]+. It was readily soluble in petroleum ether, dichloromethane, chloroform, and poorly soluble in methanol. Thin layer chromatography showed no dark spots under ultraviolet light. The spectral details are shown in Table S11, and the spectrum is shown in Figure S18. After comparing with the literature [33], the compound was identified as n-tetradecanoic acid.

(11) EUD-12: Yellow solid, molecular formula: C_29_H_56_O_3_; *m*/*z*: 453 [M+H]+. The spectral details are shown in Table S12, and the spectral diagram is provided in Figure S19. After comparing with the literature [34], the compound was identified as 2,6,10,14-tetramethyl-18-butylcarboxymethyl-12-en-17β-ol.

(12) EUD-13: White powder with the molecular formula: C_18_H_36_O_2_; *m*/*z*: 284 [M]+. It was soluble in petroleum ether and chloroform and almost insoluble in water. The spectral details are provided in Table S13, and the spectral diagram is shown in Figure S20. After comparing with the literature [35], the compound was identified as n-octadecanoic acid.

(13) EUD-14: White powder (petroleum ether–acetone extract), molecular formula: C_7_H_6_O_2_; *m*/*z*: 121 [M-H]+. After TLC development, distinct fluorescent spots were observed under 254 nm. After staining with 10% concentrated sulfuric acid and heating, it appeared purplish–red. The spectral details are provided in Table S14, and the spectrum is shown in Figure S21. After comparing with the literature [36], it was determined that the compound was p-hydroxybenzaldehyde.

(14) EUD-15: Yellow amorphous powder with the molecular formula C_15_H_12_O_5_; *m*/*z*: 271 [M-H]-. It was readily soluble in chloroform, acetone, and methanol. The spectral details are provided in Table S15, and the compound’s spectrum is shown in Figure S22. After comparing with the literature [37], the compound was identified as naringenin.

(15) EUD-16: Yellow amorphous powder with the molecular formula C_15_H_12_O_4_; *m*/*z*: 257 [M+H]+. It was soluble in organic solvents such as methanol, ethanol, and DMSO. The spectral details are provided in Table S16, and the compound’s spectral diagram is shown in Figure S23. After comparing with the literature [38], the compound was identified as glycyrrhetinic acid.

(16) EUD-17: light yellow powder with the molecular formula C_19_H_39_N; *m*/*z*: 281 [M]+. The spectral data are provided in Table S17, and the compound’s spectral diagram is shown in Figure S24. After comparing with the literature [39], the compound was identified as punigratine.

### 2.6. GC-MS Analysis of Petroleum Ether Parts

The MDR reversal activity results of each extraction showed that the Fr-S extracts exhibited the second-highest MDR reversal activity, following only the Fr-E extracts. Consequently, the chemical composition of the Fr-S was analyzed and identified using GC-MS technology.

The mass-to-charge ratio, base peak, and relative abundance of chromatographic peaks were analyzed via comparison with a mass spectrometry database and related literature, and the chemical composition of the petroleum ether extract of *Euphorbia uralensis* was determined.

The results showed that 22 chemical components were detected in the petroleum ether extract, including palmitic acid, palmitic acid methyl ester, (+)-calonyctone B, caryophyllene, and (E)-Atlantone, with relative percentages of 15.86%, 12.45%, 6.69%, 2.91%, and 2.39%, respectively. All of these components were identified for the first time in *Euphorbia uralensis*, as shown in Figure S25 and Table S18. The results indicated that the main components of the Fr-S of *Euphorbia uralensis* were triterpenes, fatty acids, phenols, long-chain alkanes, and steroids.

Among the 22 identified components, the content of palmitic acid was 15.86%. Research has shown that palmitic acid possesses a wide range of pharmacological activities, including antiviral, anti-inflammatory, analgesic, and lipid metabolism regulation [40,41], and it can induce cell cycle arrest and promote apoptosis in MCF-7 [42]. In addition, palmitic acid encapsulated in poly (D,L-lactic acid-co-glycolic acid) (PLGA) nanoparticles (NPs) has been shown to significantly enhance the therapeutic effects of DOX on MCF-7, with strong MDR reversal activity [43]. Therefore, the strong MDR activity of the petroleum ether fraction may be related to its high content of fatty acid compounds. Additionally, the petroleum ether extract of *Euphorbia uralensis* can serve as a rich source for extracting compounds such as palmitic acid.

### 2.7. Evaluation of MDR Reversing Activity of Some Compounds

#### 2.7.1. Cytotoxicity of Compounds

**Figure 4:**
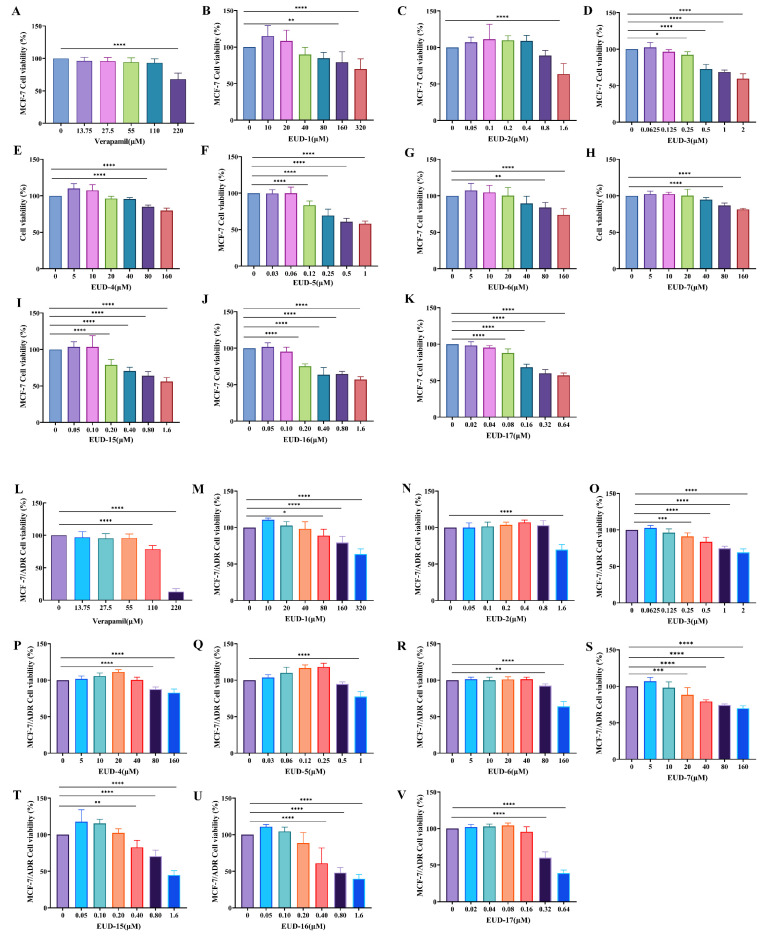
Cytotoxicity of compounds on MCF-7 cells (**A**–**K**) and MCF-7/ADR cells (**L**–**V**) (mean ± SD, n = 3). Note: Cell Viability (%) = [(OD Drug − OD Blank)/(OD contro − OD blank)] × 100. All data were compared with a blank control group (0 μg/mL of drug concentration). * *p* < 0.05, ** *p* < 0.01, *** *p* < 0.001, **** *p* < 0.0001.

The MDR reversal activity of compounds derived from *Euphorbia uralensis* was evaluated in this study using MCF-7 and MCF-7/ADR cells. Previous research has indicated a scarcity of studies and reports on the anti-tumor and MDR reversal properties of long-chain fatty acids and aromatic compounds. Additionally, due to the limited availability of monomers, the remaining 10 compounds were selected to evaluate their potential as effective agents for reversing MDR. The concentration ranges of verapamil (0–55 μM), EUD-1 (0–40 μM), EUD-2 (0–0.8 μM), EUD-3 (0–0.125 μM), EUD-4 (0–40 μM), EUD-5 (0–160 μM), EUD-6 (0–40 μM), EUD-7 (0–10 μM), EUD-15 (0–0.2 μM), EUD-16 (0–0.2 μM), and EUD-17 (0–0.4 μM) were found to be non-toxic to both MCF-7 and MCF-7/ADR cells (Figure 4).

#### 2.7.2. MDR Reversal Activity of Compounds

The results indicated that the compounds and positive controls (as shown in Figure S3 and Table 3) enhanced the sensitivity of MCF-7/ADR cells to DOX, demonstrating varying degrees of MDR reversal activity. The IC_50_ value of compound EUD-17 (16.52 ± 1.93 μM) was comparable to that of verapamil (8.88 ± 0.84 μM), and its RF value reached 3.5, indicating that compound EUD-17 exhibits superior MDR reversal activity and significantly enhances the sensitivity of MCF-7/ADR cells to DOX. Compounds EUD-1, EUD-2, and EUD-15 also exhibited MDR reversal activity, with RF values of 2.00, 1.80, and 2.00 respectively. Research has shown that Punigratane (EUD-17) exhibits inhibitory activity against efflux pumps, thereby significantly enhancing the function of efflux pumps in multidrug-resistant pneumonia strains [39]. The combination of naringenin (EUD-15) and uncaria alkaloids can effectively reverse theMDR of liver cancer cells [44]. The lipolide (EUD-2) also demonstrates potent antitumor activity and cytotoxicity due to its capacity to modulate the lipopolysaccharide-induced production of nitric oxide in RAW 264.7 cells (LPS) [45]. The findings of this study align with the existing literature, suggesting the presence of efficacious MDR reversal agents in *Euphorbia uralensis*.

#### 2.7.3. Hoechst 33258 Nuclear Staining for Compounds on Cell Apoptosis

**Figure 5:**
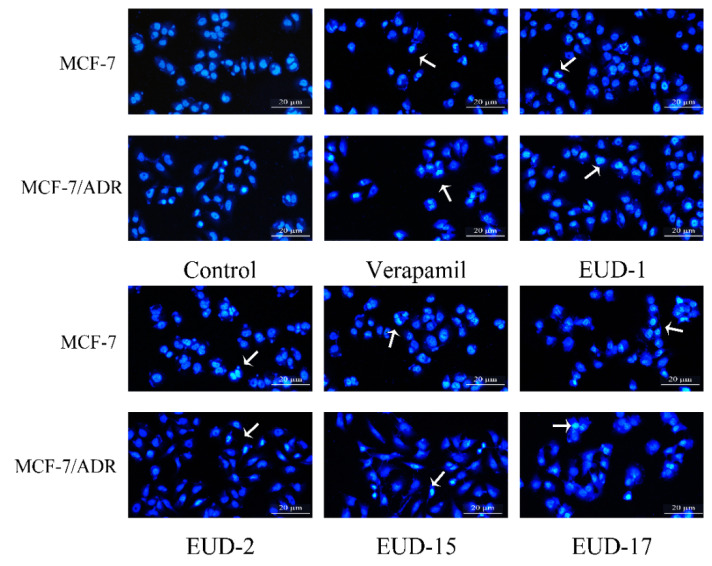
Effects of compounds on apoptotic morphology of MCF-7 and MCF-7/ADR cell injury (Hoechst 33258 staining method, ×200).

Evaluating whether it can promote tumor cell apoptosis is also one of the methods used to determine the reversal activity of MDR inhibitors [46]. In this study, Hoechst 33258 nuclear staining was employed to assess the impact of these active compounds on the apoptosis rate of MCF-7 and MCF-7/ADR cells. The apoptosis rate of MCF-7/ADR cells significantly increased following treatment with compounds EUD-1, EDU-2, EDU-15, and EDU-17 (*p* < 0.05). Following treatment, a sparser cellular arrangement was observed, with the majority of chromatin within the nucleus appearing fragmented of both MCF-7 and MCF-7/ADR cells. The results suggest that compounds EUD-1 (apoptotic rate of 5.5 ± 0.08), EDU-2 (apoptotic rate of 5.97 ± 0.05), EDU-15 (apoptotic rate of 4.17 ± 0.17), and EDU-17 (apoptotic rate of 6.4 ± 0.16) may exert MDR reversal effects by accelerating apoptosis in MCF-7 and MCF-7/ADR cells (Figure 5 and Table S19). Based on the test results of MDR reversal activity and Hoechst 33258 nuclear staining, we selected compound EUD-17 to study its mechanism of action in subsequent steps.

### 2.8. Study on the Mechanism of EUD-17 Reversing Tumor Cell MDR

#### 2.8.1. Molecular Docking Result

Currently, the two most extensively investigated mechanisms for reversing MDR are as follows: first, by inhibiting the expression of the ABCB1 protein to reduce the transport of antitumor drugs, thereby facilitating MDR reversal activity; second, by suppressing the drug efflux function of tumor cells to reverse MDR [47].

**Figure 6:**
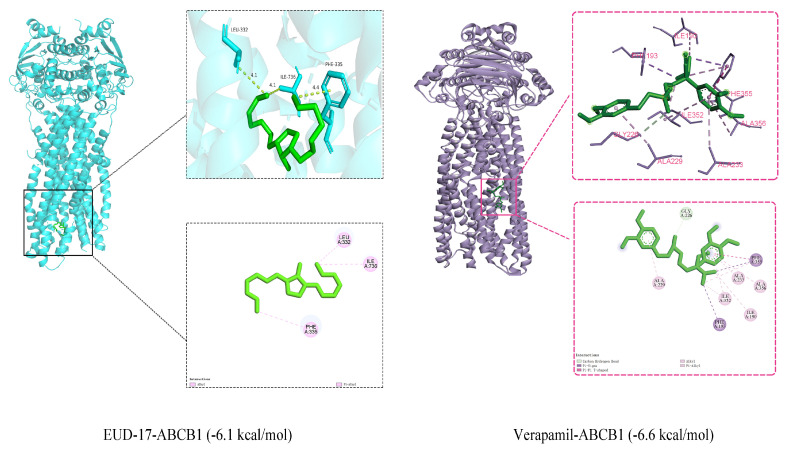
Schematic diagram of molecular docking of EUD-17, verapamil, and ABCB1 protein (binding energy ≤ −5 kcal/mol). Note: A. Alkyl and Pi-Alkyl are shown as pink dashed lines. B. carbon hydrogen bond is shown as green dashed line; Pi-sigma is shown as purple dashed line; Pi-Pi T-shaped is shown as dark pink dashed line; alkyl and Pi-alkyl are shown as light pink dashed lines.

The key target protein “ABCB1” was selected for molecular docking and affinity calculations to elucidate the mechanism of MDR reversal activity exhibited by compound EUD-17. The protein and compound exhibited an extensive contact area, strong binding, and favorable binding energies, with values of −6.1 kcal/mol for ABCB1-EUD-17 and −6.6 kcal/mol for ABCB1-verapamil (Figure 6). EUD-17 and verapamil interact with the amino acids of the ABCB1 protein through hydrogen bonding, hydrophobic interactions, and π–π stacking interactions.

#### 2.8.2. Molecular Dynamics Simulation Results

**Figure 7:**
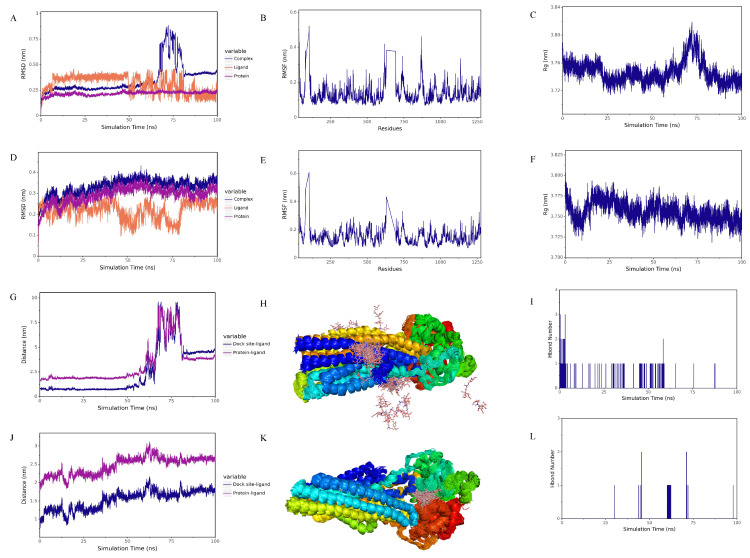
RMSD of complexes, proteins, and ligands: verapamil (**A**), EUD-17 (**D**), verapamil (**B**) and EUD-17 (**E**) with ABCB1 protein; Verapamil (**C**), EUD-17 (**F**) with ABCB1 protein Rg; Spacing of verapamil (**G**), EUD-17 (**J**) small molecules with ABCB1 protein binding sites; Simulated conformational superposition of verapamil (**H**), EUD-17 (**K**) with ABCB1 protein; The relationship between verapamil (**I**), EUD-17 (**L**), and the number of hydrogen bonds in the ABCB1 protein complexes.

The root mean square deviation (RMSD) served as a fundamental metric for assessing the stability of the system [48]. The RMSD curves of EUD-17, verapamil, and the ABCB1 target protein all fluctuated below 1 nm, indicating that their binding was stable (Figure 7A,D). The RMSD of the EUD-17–ABCB1 complex and ABCB1 protein gradually stabilized throughout the simulation, without significant fluctuations (Figure 7A).

The root mean square fluctuation (RMSF) was employed to depict the flexibility of protein amino acid residues [48]. As shown in Figure 7B,E, the RMSF curves indicate that most of the protein amino acid residues within the complexes of EUD-17, verapamil, and the ABCB1 target protein exhibit minor oscillations of less than 0.6 nm.

The Rg parameter was used to depict alterations in the overall structure and served as a measure of protein structural compactness. An increase in Rg signifies an augmented degree of system swelling [48]. The Rg of the EUD-17–ABCB1 complex gradually stabilized during the simulation, indicating that the structure of the complex also gradually stabilized (Figure 7C,F) when the Rg of the verapamil–ABCB1 complex remained stable at 0–50 ns and 82–100 ns.

The spacing between the protein and small molecule binding sites was used to analyze the state of small molecules on the protein surface [49]. As shown in Figure 7J, the distance between the binding site of ABCB1 and EUD-17 gradually stabilized during the simulation and did not fluctuate sharply. Both verapamil and EUD-17 stably bound to the initial binding site and its vicinity. The distance between the binding sites of ABCB1–verapamil, as depicted in Figure 7G, exhibited gradual stabilization during the time intervals of 0–50 ns and 82–100 ns, indicating a progressive establishment of stability in the interaction between ABCB1 and verapamil throughout this specific period.

The simulation of conformational stacking demonstrated the degree of stacking of small molecules [50]. The degree of overlap between EUD-17 and the ABCB1 protein was relatively high, indicating that the small molecules consistently bound to the initial site and its vicinity (Figure 7K). Verapamil was dispersed on the surface of the ABCB1 protein, with a high degree of overlap in some areas (Figure 7H).

Based on the comprehensive analysis of the above three sets of MD simulation results, it was concluded that in the RMSD, RMSF, Rg, and other graphs, both EUD-17 and verapamil stably bound to the ABCB1 target protein.

Hydrogen bonding was related to electrostatic interactions, and the hydrogen bonding interactions between proteins and ligands reflected the strength of the electrostatic interactions [51]. As shown in Figure 7I,L, verapamil–ABCB1 had fewer hydrogen bonding interactions than EUD-17–ABCB1.

The complex trajectories from 0–50 ns were selected for further analysis, taking into account the findings of RMSD, Rg, distance, and centroid evolution analysis, as well as interaction analysis (Figure 7 and Figure S26). The molecular mechanics Poisson–Boltzmann surface area (MM-PBSA) method was employed to compute the energy contributions associated with binding [52]. As shown in Table S20, van der Waals forces played a major role in the verapamil–ABCB1 and EUD-17–ABCB1 complexes, while hydrophobic and electrostatic interactions played a minor role. Both complexes exhibited high binding energy and affinity.

#### 2.8.3. Effect of Compound EUD-17 on Expression Level of ABCB1 Protein

**Figure 8:**
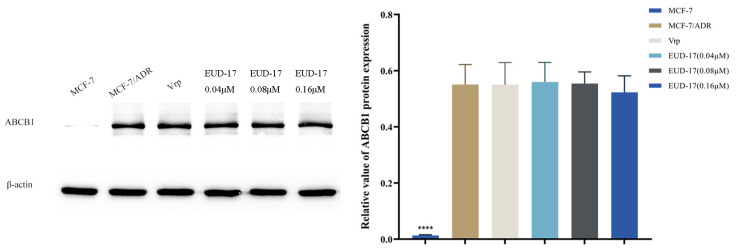
Effects of compounds EUD-17 on ABCB1 protein expression levels in MCF-7/ADR cells (treated group vs. model group, **** *p* < 0.0001).

The impact of compound EUD-17 on the expression of the ABCB1 protein in MCF-7 and MCF-7/ADR cells was investigated, with verapamil employed as a control. As demonstrated in Figure 8, the expression level of ABCB1 in drug-resistant MCF-7/ADR cells was significantly higher than in MCF-7 cells (*p* < 0.0001), indicating that the overexpression of ABCB1 protein is closely related to MDR. Treatment of MCF-7/ADR cells with verapamil and EUD-17 for 24 h showed no significant difference compared to the positive control group (*p* > 0.05) and did not lead to downregulation of ABCB1 protein expression (Figure 8).

Previous research has shown that drugs such as ciprofloxacin, osimertinib [53], antimicrobial peptides, and WD-Repeat Protein 5-0103 [54] effectively reverse ABCB1-mediated MDR by interacting with ABCB1 and inhibiting its drug transport function without altering ABCB1 expression level or affecting the expression and cellular localization of other transport proteins. Therefore, the findings of this study were consistent with previous reports suggesting that compound EUD-17 does not exert MDR reversal by inhibiting the expression of the ABCB1 protein. Alternatively, EUD-17 may potentially reverse MDR by modulating the functionality of the ABCB1 protein.

#### 2.8.4. Effects of Compound EUD-17 on Intracellular Accumulation of Rhodamine 123 (Rh123) in MCF-7/ADR

**Figure 9:**
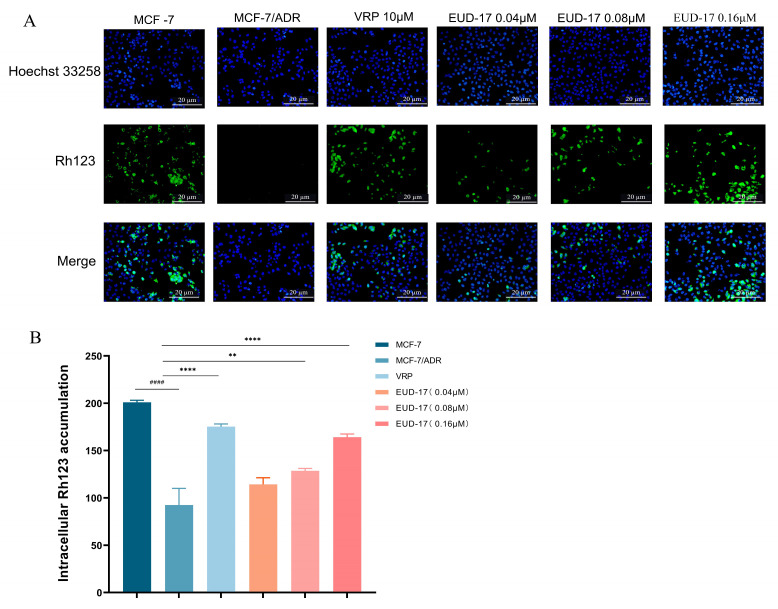
Effect of compound EUD-17 on Rh123 accumulation in MCF-7/ADR cells (treated group vs. model group, ** *p* < 0.01, **** *p* < 0.0001, model group vs. control group ^####^ *p* < 0.0001) (**B**). The accumulation of Rh123 was observed using fluorescence microscopy (**A**), and the average fluorescence intensity was quantified using ImageJ software.

To further verify the effects of EUD-17 on the function of the ABCB1 protein, we conducted Rh123 immunofluorescence experiments. Following EUD-17 treatment, there was a significant, dose-dependent increase in the accumulation of Rh123 in MCF-7/ADR cells (*p* < 0.05). Compound EUD-17 at 0.16 μM had the same effect as the positive control drug verapamil (Figure 9).

Research has shown that various natural MDR reversal agents, such as cannabinoid diterpenes, flavonoids, and steroids, can reverse MDR by inhibiting the function of the ABCB1 protein and increasing the accumulation of Rh123 in cells [55]. The findings of this study aligned with existing literature, providing preliminary evidence to support the proposed mechanism of action for compound EUD-17 in reversing MDR. Finally, due to the limited quantity of isolated compound available, we were unable to conduct further in-depth research.

## 3. Materials and Methods

### 3.1. Drugs and Reagents

The MCF-7 and MCF-7/ADR cell lines were sourced from Hunan Fenghui Biotechnology Co., Ltd. (Changsha, China) DMSO, PBS, T25 cell culture bottles, 96-well plates, and 6-well plates were purchased from Wuhan Punosai Life Technology Co., Ltd. (Wuhan, China) Verapamil and doxorubicin were purchased from MedChemExpress, USA (Shanghai, China). RPMI-1640 and RPMI-DMEM cell culture medium, trypsin/EDTA solution were purchased from Thermo Fisher Scientific Co., Ltd. (Santa Clara, CA, USA). Fetal bovine serum was purchased from Shanghai Yuanye Biological Co., Ltd. (Shanghai, China). Hoechst 33258 staining solution and rhodamine 123 (Rh123) staining solution were purchased from Shanghai Biyuntian Biotechnology Co., Ltd. (Shanghai, China). Petroleum ether, dichloromethane, ethyl acetate, and n-butyl alcohol were purchased from Tianjin Fuyu Fine Chemical Co., Ltd. (Tianjin, China). *β*-actin antibody and PVDF membrane were obtained from Merck KGaA (Darmstadt, Germany), and the ABCB1 antibody was purchased from MedChemExpress, USA. RIPA total protein lysate and protein loading buffer were obtained from Langeco Technology Co., Ltd. (Guangzhou, China). CCK-8 and Tween-20 were obtained from Guangzhou Saiguo Biotechnology Co., Ltd. (Guangzhou, China). The BCA protein concentration kit, PMSF, and phosphorylated protease inhibitor were obtained from Wuhan Xevil Biotechnology Co., Ltd. (Wuhan, China). TBS powder, electrophoresis buffer solution, transmembrane solution powder, and skimmed milk powder were purchased from Wuhan Baikandi Biotechnology Co., Ltd. (Wuhan, China). The protease inhibitor cocktail and protein marker were provided by Shanghai Yisheng Biotechnology Co., Ltd. (Shanghai, China).

### 3.2. Plant Materials

The specimen was collected in Zhaosu County, Ili Prefecture, Xinjiang, China. The specimen was identified as *Euphorbia uralensis* by Associate Professor Wang Xiangfei from the College of Pharmacy at Shihezi University and is currently stored in the Xinjiang Ministry of Education Key Laboratory of Botanical Drug Resource Utilization under specimen number NH20220802.

### 3.3. Extraction of Herbs

A total of 5 kg of whole *Euphorbia uralensis* grass were crushed and extracted nine times with an aqueous acetone solution using permeation extraction. The extract was collected and filtered, and the solvent was removed via vacuum concentration at 45 °C using a rotary evaporator, yielding the total extract of *Euphorbia uralensis* (Fr-Z: 0.9 kg). The extract was further separated into four parts via solvent extraction and vacuum drying. The fractions obtained were the petroleum ether fraction (Fr-S: 0.4 kg), the dichloromethane fraction (Fr-E: 0.2 kg), the ethyl acetate fraction (Fr-Y: 0.1 kg), and the n-butanol fraction (Fr-J: 0.1 kg). The separation flow chart is shown in Figure S1, and the extract was stored at −20 °C.

### 3.4. Cell Culture

MCF-7 cells were cultured in DMEM medium, while adriamycin-resistant MCF-7/ADR cells were cultured in RPMI-1640 medium. Before experimental use, MCF-7/ADR cells were cultured for two weeks in complete medium without DOX. The cells were cultured in medium containing 10% fetal bovine serum and 1% penicillin–streptomycin solution, maintained at 37 °C, and incubated in a 5% CO_2_-enriched atmosphere.

Extracts of *Euphorbia uralensis* were prepared as 5 mg/mL stock solutions in chromatographically pure DMSO and diluted as needed prior to experimental use. Ten compounds were formulated into a series of gradient concentrations (0–320 µM) as required.

### 3.5. Cytotoxicity Detection

Cells (MCF-7 and MCF-7/ADR) were seeded at 8 × 10^3^ cells per well in 96-well microplates during their logarithmic growth phase. Extracts of varying concentrations (0 to 100 µg/mL) were added to the cells and incubated for 24 h. Verapamil is a first-generation resistance reversal agent targeting ABCB1 and is one of the most commonly used antiarrhythmic drugs in clinical practice. Studies have shown that verapamil can reverse MDR in tumor cells by increasing the accumulation of intracellular chemotherapeutic agents, and it is the earliest applied MDR reversal agent [1]. Therefore, verapamil was chosen as a positive control to evaluate the MDR reversal activity of the isolated compounds in this study. Verapamil and other compounds were added at concentrations ranging from 0 to 220 µM. This study included three distinct groups: the solvent group (without compounds), the blank control group (no treatment), and the positive control group (verapamil). After 48 h of incubation, the supernatant was discarded, and the tumor cells were washed twice with PBS. Following the addition of 10 µL of CCK-8 reagent to each well, the plates were incubated for 1–4 h. Absorbance (A) at 450 nm was measured using an enzyme-linked immunosorbent assay (ELISA) reader. GraphPad Prism 9.0 was used to analyze the data from triplicate experiments. Verapamil and other compounds were added to cells at concentrations ranging from 0 to 220 µM, and the rest of the experimental procedure was the same as described above.

Cell viability was computed using the following formula:

$$\mathrm{Cell}\;\mathrm{Viability}\;(\%)=\frac{\left(\mathrm{OD}\;\mathrm{Drug}\;-\;\mathrm{OD}\;\mathrm{Blank}\right)}{(\mathrm{OD}\;\mathrm{control}\;-\;\mathrm{OD}\;B\mathrm{lank})}\;\times \;100$$

### 3.6. MDR Reversal Activity Detection

Cells (MCF-7 and MCF-7/ADR) were seeded at 8 × 10^3^ cells per well in 96-well microplates during their logarithmic growth phase. Extracts, compounds, and verapamil (positive control) were added to the cells and incubated for 24 h. Verapamil (55 μM) and DOX (negative control) were added separately to the cells. Furthermore, based on its maximum non-toxic dose, DOX was administered at concentrations ranging from 0 to 50 µM to the cells. Following a 24 h incubation period, the supernatant was discarded, and the cells were washed twice with PBS. Then, 10 µL of CCK-8 reagent was added to each well and incubated for 1 to 4 h. Optical density was measured at 450 nm using the a plate reader. The experiment was performed in triplicate. Verapamil (55 μM) and DOX (negative control) were added separately. The remaining experimental procedure followed the steps outlined above. Following data processing, the IC_50_ and reversal factor (RF) values were calculated using GraphPad Prism 9.0 software.

$$\mathrm{Equation}=\frac{{\mathrm{IC}}_{50(\mathrm{DOX})}}{{\mathrm{IC}}_{50(\mathrm{DOX}+\mathrm{compound})}}$$

### 3.7. GC-MS Analysis of Fr-S in Euphorbia uralensis

The Fr-S extracts were obtained using the same plant extraction procedure as 3.1.3, and a 2 mg/mL stock solution was prepared in chromatographic-grade acetonitrile.

The chromatographic column comprised HP-5MS Ultra Inert (30 m × 250 µm × 0.25 µm) with a temperature range of −60 °C to 325 °C (350 °C). The initial temperature was set to 40 °C, and the final temperature was set to 280 °C, with a heating rate of 10 °C/min. Heating was continued at a rate of 5 °C/min until reaching 300 °C, then held for 10 min. The sample injection volume was 1 μL, with a split ratio of 10:1 and a split flow rate of 10 mL/min. High-purity helium was used as the carrier gas, and electron ionization (EI) served as the ionization method, with the electron energy set at 70 eV. The MS ion source temperature was maintained at 230 °C, with a solvent delay time of 3 min. Qualitative analysis was performed via comparison with the NIST17.L standard spectral database.

### 3.8. Research on the Chemical Composition of Fr-E in Euphorbia uralensis

The Fr-E-3 fraction from the Fr-E extract exhibited the most potent MDR reversal activity, while the Fr-E-1 and Fr-E-2 fractions also showed some activity, as indicated by the results of activity-guided separation. Therefore, subsequent work focused on the separation and purification of the Fr-E extract. The separation and purification steps are illustrated in Figure S1.

#### 3.8.1. Separation and Purification of the Fr-E-1 Fraction

The Fr-E-1 fraction (50.65 g) was dissolved in petroleum ether and thoroughly mixed. It was then stirred with 400 g of 100–200 mesh silica gel until the mixture was uniform. Excess solvent was removed by drying, and the mixture was ground into a fine powder and sieved through a 160-mesh screen. Separation was performed using a dry loading method. A gradient elution was conducted using a solvent mixture of petroleum ether and dichloromethane, with ratios of 100:1, 80:1, 60:1, 40:1, 20:1, 10:1, 5:1, 1:1, and 0:100. Each fraction was analyzed via TLC during the elution process, and similar fractions were combined based on the results, yielding four sub-fractions: Fr-E-1-1 to Fr-E-1-4.

Fraction Fr-E-1-1 (10.3 g) was separated using silica gel chromatography with both dry packing and wet loading. A gradient elution was performed using a solvent mixture of petroleum ether and dichloromethane at the following ratios: 100:1, 90:1, 80:1, 70:1, and 60:1 in sequence. During the elution process, each fraction was analyzed using thin layer chromatography (TLC), and similar fractions were combined into two fractions: Fr-E-1-1A (4.7 g) and Fr-E-1-1B (3.2 g).

Fraction Fr-E-1-1A (4.7 g) was obtained after several rounds of separation and purification steps, yielding a small number of impurities (80 mg). To further purify the compound, a finer silica gel chromatography column was employed, with a mixed solvent of petroleum ether and dichloromethane as the eluent at a ratio of 80:1. After additional purification steps using silica gel chromatography, compound EUD-5 (10.5 mg) was successfully isolated.

Fraction Fr-E-1-1B (3.2 g) was subjected to gradient elution using a silica gel column chromatography with eluent ratios of 100:1, 90:1, and 80:1. After multiple separations, two major spots remained that could not be effectively purified. Therefore, a finer silica gel column was employed for purification with a mixed solvent of petroleum ether and dichloromethane (90:1) as the eluent. Finally, two monomeric compounds, EUD-8 (8.6 mg) and EUD-9 (9.7 mg), were isolated.

Fraction Fr-E-1-3 (7.3 g) was separated using silica gel column chromatography. The column was packed using the wet method, and the sample was loaded using the dry method to ensure effective loading of the fraction onto the chromatography column. A solvent mixture of petroleum ether and ethyl acetate was used as the eluent, with elution ratios of 80:1, 60:1, 40:1, 20:1, and 10:1. After TLC detection, the fractions were combined into three sub-fractions, Fr-E-1-3 (A-C).

Fraction Fr-E-1-3A (2.7 g) was eluted with petroleum ether and dichloromethane (70:1), yielding a yellow oil (1.6 g). Compounds EUD-10 (10.5 mg) and EUD-11 (15.9 mg) were isolated via silica gel column chromatography.

Fraction Fr-E-1-3B (1.8 g) was repeatedly eluted through a pressurized silica gel chromatography column, yielding an impure white oil (1.5 g). The fraction was further purified using a pressurized silica gel column with a mixed solvent of petroleum ether and dichloromethane (50:1, 30:1, and 15:1) as the eluent, ultimately yielding compound EUD-12 (9.7 mg).

#### 3.8.2. Separation and Purification of Fr-E-2 Fraction

Fraction Fr-E-2 (3.3 g) was eluted using a gradient of petroleum ether and dichloromethane with ratios of 15:1, 10:1, 5:1, 1:1, and 0:100. After TLC detection, the eluted fractions were combined into five fractions Fr-E-2 (A-C).

Compound EUD-13 (8.8 mg) was isolated from fraction Fr-E-2A through repeated silica gel column chromatography.

Fraction Fr-E-2C (1.8 g) was eluted using a gradient of petroleum ether and dichloromethane at ratios of 10:1, 5:1, and 1:1, resulting in the isolation of compound EUD-14 (12.8 mg).

#### 3.8.3. Separation and Purification of Fr-E-3 Fraction

Fraction Fr-E-3 (62.35 g) was eluted using a gradient of petroleum ether and ethyl acetate as the mobile phase with ratios of 70:1, 50:1, 25:1, 10:1, 5:1, 1:1, and 0:100. After TLC analysis, the eluted fractions were combined into four fractions Fr-E-3 (A-D).

Fraction Fr-E-3A (8.6 g) was analyzed using TLC, revealing several distinct spots. Using a petroleum ether and ethyl acetate mobile phase with ratios of 70:1, 50:1, 25:1, 10:1, 5:1, 1:1, and 0:100, compounds EUD-1 (10.3 mg) and EUD-2 (16.7 mg) were eluted and isolated.

Fraction Fr-E-3B (3.1 g) was found to have multiple concentrated spots after TLC analysis. Sephadex LH-20 gel was chosen for purification with a mobile phase of dichloromethane and methanol (1:1) for isocratic elution. According to the TLC analysis, the 7th tube showed relatively pure and clear spots, which were weighed (80 mg) and then loaded onto a finer Sephadex LH-20 gel for elution with an isocratic gradient of dichloromethane and methanol (1:1), resulting in the isolation of the compound EUD-3 (5.6 mg). Meanwhile, the TLC results indicated that the 4th tube contained three spots with very close Rf values, which were further purified using preparative TLC, ultimately yielding the compounds EUD-4 (6.6 mg) and EUD-6 (7.5 mg).

Fraction Fr-E-3C (6.0 g) was obtained using a gradient elution with a petroleum ether and ethyl acetate in ratios of 30:1, 15:1, 10:1, 5:1, 1:1, and 0:100. Following sample detection and combination, three sub-fractions Fr-E-3C (1–3) were obtained. Fraction Fr-E-3C-1 was further purified using repeated pressure silica gel column chromatography, which led to the isolation of the compounds EUD-7 (7.4 mg) and EUD-17 (3.5 mg).

After the TLC analysis, fraction Fr-E-3D (4.8 g) showed fewer spots of varying intensities. Purification was performed using Sephadex LH-20 column chromatography with a dichloromethane and methanol (1:1) isocratic elution. Following TLC analysis and fraction merging, 12 sub-fractions, Fr-E-3D (1–12) were obtained. Among these, Fr-E-3D-3 and Fr-E-3D-8 appeared relatively pure. TLC analysis at 254 nm indicated the presence of only three spots in these fractions. Semi-preparative liquid chromatography was used for further separation and purification with acetonitrile and water (70:30, flow rate 2 mL/min, injected 5 times), resulting in the isolation of the compounds EUD-15 (5.8 mg) and EUD-16 (6.9 mg).

### 3.9. Hoechst 33258 Nuclear Staining for Apoptosis Detection

To detect the effects of the compounds on cell apoptosis, we used the Hoechst 33258 nuclear staining method with minor modifications based on previous studies [56].

Cells (MCF-7 and MCF-7/ADR) were seeded at 3 × 10^8^ cells per well in 6-well plates during their logarithmic growth phase and incubated for 24 h. The respective concentration of active compounds was added to each cell group, while the control group received verapamil in drug-free medium. After 48 h of incubation, the cells were fixed with paraformaldehyde, rinsed, and stained with Hoechst 33258 solution. The cells were treated in the dark for 15 min and then photographed and recorded using a fluorescence microscope. The number of apoptotic cells in each slice was continuously monitored among a total of 1000 cells, and the apoptotic rate was subsequently calculated.

$$\mathrm{Cell}\;\mathrm{Viability}\;(\%)=\frac{\mathrm{number}\;\mathrm{of}\;\mathrm{apoptotic}\;\mathrm{cells}}{1000}\times 100$$

### 3.10. Study on the Mechanism of Action of EUD-17 in Reversing MDR in Tumor Cells

#### 3.10.1. Molecular Docking

The ABCB1 protein was used as the receptor, with verapamil and EUD-17 selected as the junction ligands. The structures of the top 10 compounds from the “C-T-P” network were downloaded from the PubChem database and exported in SDF format. ChemBio3D Ultra 14.0 software was used to open the small molecules. The “Calculation” > “MM2” > “Minimize Energy” options were selected to minimize the coordination energy, and the results were saved in PDB format. AutoDockTools-1.5.6 was used to add hydrogens and calculate charges for small molecules, which were then designated as ligands and saved in pdbqt format for backup. The protein structure was downloaded from the ProteinDataBank and saved as a PDB format file [57]. Using AutoDockTools-1.5.6, the protein was opened, dehydrated, and hydrogenated, and the charge calculations were performed. After designating the protein as the receptor, the docking area was defined. Hydrogens were added to the small molecule, charges were calculated, and it was selected as the ligand and saved in PDBQT format. Preprocessing steps, including dehydration, hydrogenation, and charge calculations, were performed using AutoDock Tools 1.5.6, and the files were saved in pdbqt format. AutoDockTools-1.5.6 and AutodockVina 4.21 were used for semi-flexible molecular docking of the ligand and receptor, and affinity detection was performed. The results were visualized using pymol 2.3.0.

#### 3.10.2. Molecular Dynamics (MD) Simulation

The molecular mechanisms of verapamil and EUD-17 binding to the ABCB1 protein were investigated using MD simulations to analyze their dynamic binding process. Appropriate molecular docking conformations were selected for MD simulation [2]. MD simulations for EUD-17, verapamil, and ABCB1 were performed using GROMACS (version 2022.2) software. The protein structure was refined using SPDBV 4.10, and the docked ligand was preserved in mol2 format using PyMOL 2.3.0. Ligand and topology files were processed using Sobpob 1.0 (dev3.1). GROMACS was used to process protein ensembles and topological structures, including the incorporation of cassettes, water molecules, and ions. Ion concentration equalization, energy minimization, and NVT pre-equilibration were performed to complete the kinetic simulation protocol. Following the simulation, GROMACS was used to evaluate protein–ligand binding stability by calculating the root mean square deviation (RMSD) and radius of gyration (RG).

#### 3.10.3. Effects of Compound EUD-17 on ABCB1 Protein Expression Levels

The MCF-7 and MCF-7/ADR cells were exposed to varying concentrations of chemicals. RIPA lysate was used to extract total cellular proteins, and protein concentrations were quantified using a BCA protein assay kit. SDS-PAGE loading buffer was added to the supernatant, and the mixture was boiled at 95–100 °C for 5 min to denature the proteins. The proteins were transferred onto a PVDF membrane, which was then blocked for 1 h at room temperature with a 5% blocking solution and incubated overnight at 4 °C with the primary antibody. The membrane was washed five times with TBST and then incubated with secondary antibodies for 30 min at room temperature. Protein detection was carried out using the ECL chemiluminescence method. The grey value of each protein band was analyzed using ImageJ software. The relative expression level of the target protein was calculated by dividing its grey value by that of the internal reference protein.

#### 3.10.4. Effects of Compound EUD-17 on Intracellular Rh123 Accumulation

To facilitate cell attachment, MCF-7 and MCF-7/ADR cells in the logarithmic growth phase were seeded at a density of 1 × 10^6^ cells/mL on 6-well microculture plates and incubated for 24 h at 37 °C in a CO_2_ incubator. Verapamil (55 µM) and EUD-17 at concentrations of 0.04 µM, 0.08 µM, and 0.16 µM were then added to MCF-7/ADR cells. The cells were cultured in a CO_2_ incubator for 24 h at 37 °C.

After incubation, the cells were washed with PBS and fixed with paraformaldehyde. Following fixation, the nuclei were stained with a Hoechst 33258 solution for 15 min under light-protected conditions to allow for cell localization. The dye was removed via washing with PBS, and Rh123 staining solution was added under dark conditions. The cells were incubated at room temperature for 30 min, followed by three washes with PBS. Intracellular accumulation of Rh123 was observed using fluorescence microscopy, and the average fluorescence intensity was quantified using ImageJ java 8 software.

#### 3.10.5. Data Statistics and Analysis

Statistical analysis was performed using GraphPad Prism 9.0, and all data are expressed as mean ± standard deviation (SD). A one-way ANOVA was used to analyze the findings, with results considered statistically significant at *p* < 0.05.

## 4. Conclusions

This study is the first to prepare effective parts and active components of *Euphorbia uralensis* using an MDR reversal activity-oriented method. Seventeen compounds, including one new compound and sixteen known compounds, were isolated and purified from the active parts and fractions. The main types of compounds identified were steroids, sesquiterpenes, triterpenes, and flavonoids, all of which were reported for the first time in *Euphorbia uralensis*. The chemical constituents of the petroleum ether fraction of *Euphorbia uralensis* were analyzed using GC-MS, identifying 22 compounds. The main components of this fraction were triterpenes, fatty acids, phenols, long-chain alkanes, and steroids, all of which were reported for the first time in *Euphorbia uralensis*. Among these compounds, palmitic acid reached a content level of 15.86%.

We evaluated the MDR reversal activity of compounds through in vitro cell experiments. The IC_50_ values of compounds EUD-1, EUD-2, EUD-15, and EUD-17 were significantly higher than those of the control group, with RF values of 2.0, 1.8, 2.0, and 3.5, respectively. We selected the compound punigratine (EUD-17) and evaluated its MDR reversal activity by assessing cell apoptosis using the Hoechst 33258 nuclear staining method and Western blot (WB) analysis. The results indicated that compound punigratine, as a substrate of the ABCB1 protein, can compete with anti-tumor drugs by inhibiting the efflux function of ABCB1 protein.

## Figures and Tables

**Table 1:** The he half maximal inhibitory concentration (IC_50_) and reversal fold (RF) values of MCF-7/ADR cells after extracts from each extraction and Fr-E flow fraction in combination with doxorubicin (DOX, 0–50 μM).

Extraction Site	Drug Concentration (μg/mL)	IC_50_ (Mean ± SD/µM)	RF
Control (DOX)	-	65.62 ± 5.54	1.0
Verapamil + DOX	55 µM	8.34 ± 1.35	7.9
Fr-S + DOX	6.25	30.57 ± 5.48	2.1
Fr-Y + DOX	12.5	57.74 ± 2.90	1.1
Fr-J + DOX	25	64.23 ± 5.83	1.0
Fr-E + DOX	12.5	21.99 ± 1.94	3.0
Fr-Z + DOX	12.5	56.74 ± 6.72	1.2
Fr-E-1 + DOX	12.5	31.86 ± 10.61	2.2
Fr-E-2 + DOX	12.5	33.96 ± 13.36	2.0
Fr-E-3 + DOX	12.5	25.60 ± 12.50	2.7
Control (DOX)	-	68.88 ± 14.75	1.0
Verapamil + DOX	55 µM	12.03 ± 2.19	5.7

Note: RF = (IC_50_ without inhibitor)/(IC_50_ with inhibitor).

**Table 2:** EUD-1’s ^1^H-NMR (chloroform-d, 400 Hz) and ^13^C-NMR (chloroform-d, 100 Hz) data.

Number	^13^C	^1^H
1	39.1	1.85, m, 1H; 1.07, m, 1H
2	29.2	1.91, m, J = 5.6, 3.5, 1Hα; 1.63, m, 1Hβ
3	77.9	3.56, m, 1H
4	35.7	2.12, m, 1H; 2.27, m, 1H
5	139.3	-
6	119.0	5.37, d, J = 4.8, 1H
7	38.8	2.32, m, 2H
8	73.9	-
9	44.5	1.49, dd, J = 13.0, 3.3, 1H
10	37.4	-
11	24.2	2.03, m, H; 1.65, m, 1H
12	74.9	4.70, dd, J = 12.0, 4.0, 1H
13	54.1	-
14	86.4	-
15	37.1	1.4, m, 1H; 2.06, m, 1H
16	24.8	1.83, m, 2H
17	57.2	3.14, m, 2H
18	12.1	1.19, s, 3H
19	18.3	1.18, s, 3H
20	217.8	-
21	33.1	2.21, s, 3H
1′	167.2	-
2′	113.5	5.72, brs, 1H
3′	167.2	-
4′	38.5	2.32, m, 1H
5′	21.0	1.08, d, J = 6.8, 3H
6′	21.0	1.08, d, J = 6.8, 3H
7′	16.6	2.17, s, 3H
1″	95.6	4.78, dd, J = 9.6, 1.6, 1H
2″	39.2	2.19, m, 1Hα; 1.58, m, 1Hβ
3″	77.6	3.63, m, 1H
4″	72.6	3.21, dd, J = 9.2, 3.2, 1H
5″	70.9	3.58, m, 1H
6″	18.4	1.28, d, J = 6.2, 3H
7″	57.4	3.43, s, 3H
8-OH	-	5.19, brs, 1H
14-OH	-	5.21, brs, 1H
4″-OH	-	3.61, dd, 1H

**Table 3:** IC_50_ values of MCF-7/ADR cells after combination of compounds with DOX.

Compound	Dose Concentration (µM)	IC_50_ (Mean ± SD/μM)	RF
Control (DOX)	-	57.08 ± 7.83	1.00
Verapamil + DOX	55.00	8.88 ± 0.84	6.40
EUD-1 + DOX	40.00	29.04 ± 1.61	2.00
EUD-2 + DOX	0.80	31.83 ± 4.15	1.80
EUD-3 + DOX	0.125	51.31 ± 7.47	1.10
EUD-4 + DOX	40.00	41.55 ± 7.86	1.40
EUD-5 + DOX	160.00	44.35 ± 5.50	1.30
EUD-6 + DOX	40.00	47.32 ± 4.36	1.20
EUD-7 + DOX	10.00	46.21 ± 5.83	1.20
EUD-15 + DOX	0.20	27.88 ± 3.17	2.00
EUD-16 + DOX	0.20	43.77 ± 5.27	1.30
EUD-17 + DOX	0.16	16.52 ± 1.93	3.50

## Data Availability

The original contributions presented in this study are included in the article and Supplementary Materials. Further inquiries can be directed to the corresponding author.

## Supplementary Materials

The following supporting information can be downloaded at: https://www.mdpi.com/article/10.3390/ijms26010412/s1.

## References

[1] Watanabe T., Rikitake R., Kakuwa T., Ichinose Y., Niino M., Mizushima Y., Ota M., Fujishita M., Tsukada Y., Higashi T. (2023). Time to treatment initiation for six cancer types: An analysis of data from a nationwide registry in japan. World J. Surg..

[2] Tang S.H., Li S.Y., Shi X.W., Sheng L.X., Mu Q.T., Wang Y., Zhu H.L., Xu K.H., Zhou M., Xu Z.J. (2024). CALCRL induces resistance to daunorubicin in acute myeloid leukemia cells through upregulation of XRCC5/TYK2/JAK1 pathway. Anti-Cancer Drugs.

[3] Ramirez M., Rajaram S., Steininger R.J., Osipchuk D., Roth M.A., Morinishi L.S., Evans L., Ji W.Y., Hsu C.-H., Thurley K. (2016). Diverse drug-resistance mechanisms can emerge from drug-tolerant cancer persister cells. Nat. Commun..

[4] Shankaraiah N., Nekkanti S., Ommi O., PS L.S. (2019). Diverse Targeted Approaches to Battle Multidrug Resistance in Cancer. Curr. Med. Chem..

[5] Holohan C., Van Schaeybroeck S., Longley D.B., Johnston P.G. (2013). Cancer drug resistance: An evolving paradigm. Nat. Rev. Cancer.

[6] Newman D.J., Cragg G.M. (2016). Natural products as sources of new drugs from 1981 to 2014. J. Nat. Prod..

[7] Guo Q., Cao H.Y., Qi X.H., Li H.K., Ye P.Z., Wang Z.G., Wang D.Q., Sun M.Y. (2017). Research Progress in Reversal of Tumor Multi-drug Resistance via Natural Products. Anti-Cancer Agents Med. Chem..

[8] Zou J.Y., Chen Q.L., Luo X.C., Damdinjav D., Abdelmohsen U.R., Li H.Y., Battulga T., Chen H.B., Wang Y.Q., Zhang J.Y. (2023). Natural Products Reverse Cancer Multi drug Resistance. Front. Pharmacol..

[9] Wei J., Liu Z., He J., Liu Q., Lu Y., He S., Yuan B., Zhang J., Ding Y. (2022). Traditional Chinese medicine reverses cancer multidrug resistance and its mechanism. Clin. Transl. Oncol. Off. Publ. Fed. Span. Oncol. Soc. Natl. Cancer Inst. Mex..

[10] Jiang D.J., Guo S.J., Kang A., Ju Y.H., Li J.X., Yu S., Bao B.H., Cao Y.D., Tang Y.P., Zhang L. (2020). Comparison of the short-chain fatty acids in normal rat faeces after the treatment of Euphorbia kansui, a traditional Chinese medicine for edoema. Pharm. Biol..

[11] Zhu H., Ren X.X., Huang Y.B., Su T., Yang L. (2023). Chemical Constituents of Euphorbia stracheyi Boiss (Euphorbiaceae). Metabolites.

[12] Benmerache A., Alabdul Magid A., Labed A., Kabouche A., Voutquenne-Nazabadioko L., Hubert J., Morjani H., Kabouche Z. (2017). Isolation and characterisation of cytotoxic compounds from Euphorbia clementei Boiss. Nat. Prod. Res..

[13] Grauso L., de Falco B., Lucariello G., Capasso R., Lanzotti V. (2021). Diterpenes from Euphorbia myrsinites and their anti-inflammatory property. Planta Medica.

[14] Magozwi D.K., Dinala M., Mokwana N., Siwe-Noundou X., Krause R.W.M., Sonopo M., McGaw L.J., Augustyn W.A., Tembu V.J. (2021). Flavonoids from the Genus Euphorbia: Isolation, Structure, Pharmacological Activities and Structure–Activity Relationships. Pharmaceuticals.

[15] Hamada H., Catherine L., Hassina H., Abdulmagid A.M., Laurence M., Mohammed B. (2007). Diterpenoids and triterpenoids from Euphorbia guyoniana. Phytochemistry.

[16] Rojas-Jiménez S., Valladares-Cisneros M.G., Salinas-Sánchez D.O., Pérez-Ramos J., Sánchez-Pérez L., Pérez-Gutiérrez S., Campos-Xolalpa N. (2024). Anti-Inflammatory and Cytotoxic Compounds Isolated from Plants of *Euphorbia* Genus. Molecules.

[17] Gao J., Aisa H.A. (2017). Terpenoids from Euphorbia soongarica and Their Multidrug Resi stance Reversal Activity. J. Nat. Prod..

[18] Zeng R., Wang X.X., Cui X.H., Yang Q., Zhu X.X., Wang Y.J. (2024). Stellera chamaejasme against multidrug resistance of triple-negative breast cancer MDA-MB-231 cell through Nrf2. China J. Chin. Mater. Medica.

[19] Bawazeer S., Rauf A., Mabkhot Y.N., Al-Showiman S.S., Patel S., Gul S., Raza M., Molnar J., Szabo D., Csonka A. (2021). Isolation of Bioactive Compounds from Pistacia integerrima with Promising Effects on Reverse Cancer Multidrug Resistance. Russ. J. Bioorganic Chem..

[20] Zhang W.K., Zhang X.Q., Ye W.C. (2007). Chemical constituents of the aerial parts of Euphorbia sororia. J. China Pharm. Univ..

[21] Hasan A., Tang D., Nijat D., Yang H.Q., Aisa H.A. (2021). Diterpenoids from Euphorbia glomerulans with potential reversal activities against P-glycoprotein-mediated multidrug resistance. Bioorganic Chem..

[22] Gao J., Chen Q.B., Liu Y.Q., Xin X.L., Yili A., Aisa H.A. (2016). Diterpenoid constituents of Euphorbia macrorrhiza. Phytochemistry.

[23] Yang H.Q., Mamatjan A., Tang D., Aisa H.A. (2021). Jatrophane diterpenoids as multidrug resistance modulators from Euphorbia sororia. Bioorganic Chem..

[24] Huang S.X., Yang J., Xiao W.L., Zhu Y.L., Li R.T., Li L.M., Pu J.X., Li X., Li S.H., Sun H.D. (2006). Three Novel Terpenoids from Schisandra pubescens var. Pubinervis. Helv. Chim. Acta.

[25] Richter R., Basar S., Koch A., König W.A. (2005). Three sesquiterpene hydrocarbons from the roots of Panax ginseng C. A. Meyer (Araliaceae). Phytochemistry.

[26] Tijjani A., Ndukwe I.G., Ayo R.G. (2012). Isolation and characterization of lup-20 (29)-ene-3, 28-diol (Betulin) from the stem-bark of Adenium obesum (Apocynaceae). Trop. J. Pharm. Res..

[27] Ma M., Shang X.Y., Wang S.J., Li S., Yang Y.C., Shi J.G. (2007). Chemical constituents from branch of Macaranga adenantha and their TNF-α inhibitory activity. China J. Chin. Mater. Medica.

[28] Jiao X.Y., Li J., Sun L.Q., Shi Z.C., Wang J.L., Zhao M., Zhang S.J. (2022). Chemical constituents from leaves of Zanthoxylum bungeanum Maxim. J. Qiqihar Univ. (Nat. Sci. Ed.).

[29] Wansi J.D., Chiozem D.D., Tcho A.T., Toze F.A.A., Devkota K.P., Ndjakou B.L., Wandji J., Sewald N. (2010). Antimicrobial and antioxidant effects of phenolic constituents from Klainedoxa gabonensis. Pharm. Biol..

[30] Liang N., Yang X.X., Wang G.C., Wu X., Yang Y.T., Luo H.J., Li Y.L. (2012). Study on the chemical constituents of Elephantopus mollis. J. Chin. Med. Mater..

[31] Meng W.T., Meng X., Niu L.T., Zhang S.S., Ouyang C.J., Ding C.H., Zhu L.J., Zhang X. (2023). A new bibenzyl derivative from stems of *Dendrobium officinale*. Chin. Med. Mag. China.

[32] Xu F., Wu Z.H., Yi Q.Q., Zhang T.L., Zhao L., Wang H.Q. (2021). Chemical constituents from ethyl acetate fraction of Callicarpa giraldii and their anti-inflammatory activities. Chin. Tradit. Pat. Med..

[33] Huang X.F., Luo J., Zhang Y., Kong L.Y. (2006). Chemical Constituents of Asparagus officinalis. Chin. J. Nat. Med..

[34] Chung I.M., Ali M., Chun S.C., Jin C.W., Cho D.H., Hong S.B., Ahmad A. (2007). New Aliphatic Alcohol and Ester Constituents from Rice Hulls of *Oryza sativa*. Chin. J. Chem..

[35] Wang X.B. (2018). Study on the Chemical Constituents of the Tropical Seagrass Enhalus acoroides. J. Hainan Norm. Univ. (Nat. Sci.).

[36] Lin L.L., Huang F., Chen S.B., Yang D.J., Chen S.L., Yang J.S., Xiao P.G. (2005). Chemical constituents in roots of Polygala fallax and their anti-oxidation activities in vitro. China J. Chin. Mater. Medica.

[37] Zhou Y.J., Wang J.H., Xue Y.F., Xu H., Chou G.X., Wang Z.T. (2021). Study on chemical constituents from active ethyl acetate fraction of *Dendrobium officinale*. Chin. Tradit. Herb. Drugs.

[38] Zhang Y.Y., Yan Y., He J., Yang T., Li X.X., Zhang W.K., Xu J.K. (2023). Study on the Chemical Constituents of Roots from *Euphorbia* ebracteolata Hayata. Chin. Pharm. J..

[39] Rafiq Z., Narasimhan S., Vennila R., Vaidyanathan R. (2016). Punigratane, a novel pyrrolidine alkaloid from Punica granatum rind with putative efflux inhibition activity. Nat. Prod. Res..

[40] Liu X.X., Ma H.M., He W.J., Sun Y., Lan W. (2018). Phytochemical Constituents Isolated from *Euphorbia rapulum*. Chem. Nat. Compd..

[41] Hasan A., Liu G.Y., Hu R., Aisa H.A. (2019). Jatrophane Diterpenoids from Euphorbia glomerulans. J. Nat. Prod..

[42] Rouzimaimaiti R., Maimaitijiang A., Yang H.Q., Aisa H.A. (2022). Jatrophane diterpenoids from Euphorbia microcarpa (prokh.) krylov with multidrug resistance modulating activity. Phytochemistry.

[43] Li G.H., Pan C.Y., Sun F.J., Wang X.R., Yin G.P. (2006). Reversal effect of 4 alkaloids on apoptosis of being obtained multi-drug resistance to tumour cell. Chin. Tradit. Pat. Med..

[44] Qiu W.L., Sun Q.Q., Qiu B. (2022). Action Mechanism Progress of Chinese Medicinal and Its Active Ingredients in Treatment of Hepatic Carcinoma. Inf. Tradit. Chin. Med..

[45] Ding L., Görls H., Hertweck C. (2021). Plant-like cadinane sesquiterpenes from an actinobacterial mangrove endophyte. Magn. Reson. Chem. MRC.

[46] Deng L.M., Tan T., Zhang T.Y., Xiao X.F., Gu H. (2019). miR-1 reverses multidrug resistance in gastric cancer cells via downregulation of sorcin through promoting the accumulation of in tracellular drugs and apoptosis of cells. Int. J. Oncol..

[47] Huang B.Y., Zeng Y., Li Y.J., Huang X.J., Hu N., Nan Y., Chen M.F., Yang Z.G., Chen Z.S., Zhang D.M. (2017). Uncaria alkaloids reverse ABCB1-mediated cancer multidrug resistance. Int. J. Oncol..

[48] Liu X.Y., Qin X.L., Zhong J.F. (2020). Effect of temperature on the stability of lactoglobulin: A molecular dynamics simulation. Food Ferment. Ind..

[49] Sun W., Jiao K. (2001). Interactions of Small Molecules with Protein and Its Application to Determination of Proteins. J. Qingdao Inst. Chem. Technol..

[50] Liang P.P., Wang Y.L., Huang H., Li G.Y., Wu H.Y. (2024). Identification of immune-related biomarkers of Alzheimer’s disease and prediction of medicine-food homology traditional Chinese medicines based on bioinformatics analysis and dynamic simulation. Chin. Tradit. Herb. Drugs.

[51] Kumju Y., Mira J. (2024). Determination of Potential Lead Compound from Magnolia officinalis for Alzheimer’s Disease through Pharmacokinetic Prediction, Molecular Docking, Dynamic Simulation, and Experimental Validation. Int. J. Mol. Sci..

[52] Sun F.F., Liu J.D., Xu J.F., Tariq A., Wu Y.N., Li L. (2024). Molecular mechanism of Yi-Qi-Yang-Yin-Ye against obesity in rats using network pharmacology, molecular docking, and molecular dynamics simulations. Arab. J. Chem..

[53] Jia H., Yang Q., Wang T., Cao Y., Jiang Q.Y., Ma H.D., Sun H.W., Hou M.X., Yang Y.P., Feng F. (2016). Rhamnetin induces sensitization of hepatocellular carcinoma cells to a small molecular kinase inhibitor or chemotherapeutic agents. BBA-Gen. Subjects. Gen. Subj..

[54] Moradzadeh M., Tabarraei A., Sadeghnia H.R., Ghorbani A., Mohamadkhani A., Erfanian S., Sahebkar A. (2018). Kaempferol increases apoptosi s in human acute promyelocytic leukemia cells and inhibits multidrug resistance genes. J. Cell. Biochem..

[55] Liu Y.Y., Lu Y.Y., Li X.Y., Zhang Z.R., Sun L.Z., Wang Y., He Z.R., Liu Z.Q., Zhu L.J., Fu L. (2022). Kaempferol suppression of acute colitis is regulated by the efflux transporters BCRP and MRP2. Eur. J. Pharm. Sci..

[56] Noriyoshi N., Momoyo I., Masataka M., Atsushi K. (2005). Induction of Apoptosis in Human Promyelocytic Leukemia Cell Line HL-60 by C-Benzylated Dihydrochalcones, Uvaretin, Isouvaretin and Diuvaretin (Medicinal Chemistry). Biol. Pharm. Bull..

[57] Piehl D.W., Burley S.K. (2024). Parallel delivery of experimentally determined structures and computed structure models at RCSB protein data bank (RCSB PDB, RCSB. ORG). Biophys. J..

