# Supplementary material for: Study on the Chemical Composition and Multidrug Resistance Reversal Activity of Euphorbia uralensis (Euphorbiaceae)
Source: Int J Mol Sci. 2025 Jan 6;26(1):412. doi: 10.3390/ijms26010412 (PMC11720411; doi:10.3390/ijms26010412)
Supplement: Supplementary file 1 [file ijms-26-00412-s001.zip › ijms-3350008-supplementary.pdf]

Study on the Chemical Composition and Multidrug Resistance Reversal Activity of *Euphorbia uralensis*

Yina Ding[1,2], Yuhao Liu[1,2], Qianru Dang[1,2], Zubair Akram[1,3], Anam Arshad[1,3], Haochan Zhu[1,2], Jianxiang Zhang[2], Bo Han[1,2*], Chimengul Turghun[1,2*]

1 Key Laboratory of Xinjiang Endemic Phytomedicine Resources Ministry of Education, Shihezi University College of Pharmacy, Shihezi, China
2 School of Pharmacy, Shihezi University, Shihezi, Xinjiang, China
3 School of Chemistry and Chemical Engineering, Shihezi University, Shihezi, China
Correspondence author: Chimengul Turghun, School of Pharmacy, Shihezi University, Shihezi 832000,
Xinjiang, P. R. China. Email: chimen722@sina.com
Correspondence author: Bo Han, School of Pharmacy, Shihezi University, Shihezi, Xinjiang, China Email: 524683221@qq.com

Yina Ding and Yuhao Liu contribute equally to this work and share the first author.

Supplementary materials

Table S1. Names and types of compounds obtained by isolation from *Euphorbia uralensis* 3
Table S2. ^{13}C-NMR Signal of Compound pubinernoid A 4
Table S3. ^{13}C-NMR Signal of Compound ginsinsene 5
Table S4. ^{13}C-NMR Signal of Compound Betulin 5
Table S5. ^{13}C-NMR Signal of Compound (24R)-24-stigmast-4-en-3-one 6
Table S6. ^{13}C-NMR Signal of Compound β-sitosterol 7
Table S7. ^{13}C-NMR Signal of Compound Oleanolic acid 8
Table S8. ^{13}C-NMR Signal of Compound Dibutyl phthalate 9
Table S9. ^{13}C-NMR Signal of Compound Auriculatum A 10
Table S10. ^{13}C-NMR signals of the compound di-(2-ethyl)hexyl phthalate 10
Table S11. ^{13}C-NMR Signal of Compound n-tetradecanoic acid 11
Table S12. ^{13}C-NMR signals of the compound 2,6,10,14-tetramethyl-18-butylcarboxymethyl-12-en-17β-ol 12
Table S13. ^{13}C-NMR Signal of Compound n-octadecanoic acid 13
Table S14. ^{13}C-NMR Signal of Compound p-hydroxybenzaldehyde 14
Table S15. ^{13}C-NMR signal of compound naringenin 14
Table S16. ^{13}C-NMR Signal of Compound glycyrrhetinic acid 15
Table S17. ^{13}C-NMR Signal of Compound punigratine 16
Table S18. Chemical constituents of petroleum ether parts of *Euphorbia uralensis* 17
Table S19. Effect of compounds on apoptosis rate of MCF-7/ADR cell injury ($\overline{X}$±s， n=3) 18
Table S20. Stable state binding energies and their composition (unit: kJ/mol) 18
Figure S1. Flowchart for the isolation of effective and study on chemical constituents of *Euphorbia uralensis.* 19
Figure S2. MDR reversal activity of total extracts and extraction sites 20
Figure S3. Reversal of MDR activity in MCF-7/ADR cells by compounds (n=3) 21
Figure S4. ^{1}H-NMR (top) and ^{13}C-NMR (bottom) spectra of EUD-1, a compound from *Euphorbia uralensis* 22
Figure S5. HMQC (top) and HMBC (bottom) spectra of EUD-1 compound from *Euphorbia uralensis* 23
Figure S6. ^{1}H-^{1}H COSY (top) and NOESY (bottom) spectra of EUD-1, a compound from *Euphorbia uralensis* 24
Figure S7. DEPT spectrum (top) and IR spectrum (bottom) of EUD-1 compound from *Euphorbia uralensis* 25
Figure S8. HRESI(+) MS spectrum of EUD-1 compound from *Euphorbia uralensis* 25
Figure S10. ^{1}H-NMR (top) and ^{13}C-NMR (bottom) spectra of ginsinsene, a compound from *Euphorbia uralensis* 27
Figure S11. ^{1}H-NMR (top) and ^{13}C-NMR (bottom) spectra of Betulin, a compound from *Euphorbia uralensis* 28
Figure S12. ^{1}H-NMR (top) and ^{13}C-NMR (bottom) spectra of (24R)-24-stigmast-4-en-3-one, a compound from *Euphorbia uralensis* 29
Figure S13. ^{1}H-NMR (top) and ^{13}C-NMR (bottom) spectra of β-sitosterol, a compound from *Euphorbia uralensis* 30

Figure S14. ^{1}H-NMR (top) and ^{13}C-NMR (bottom) spectra of Oleanolic acid, a compound from *Euphorbia uralensis*......31
Figure S15. ^{1}H-NMR (top) and ^{13}C-NMR (bottom) spectra of Dibutyl phthalate, a compound from *Euphorbia uralensis*......32
Figure S16. ^{1}H-NMR (top) and ^{13}C-NMR (bottom) spectra of Auriculatum A, a compound from *Euphorbia uralensis*......33
Figure S17. ^{1}H-NMR (top) and ^{13}C-NMR (bottom) spectra of di-(2-ethyl)hexyl phthalate, a compound from Euphorbia uralensis......34
Figure S18. ^{1}H-NMR (top) and ^{13}C-NMR (bottom) spectra of n-tetradecanoic acid, a compound from *Euphorbia uralensis*......35
Figure S19. ^{1}H-NMR (top) and ^{13}C-NMR (bottom) spectra of 2,6,10,14-tetramethyl-18-butylcarboxymethyl-12-en-17*β*-ol, a compound from *Euphorbia uralensis*......36
Figure S20. ^{1}H-NMR (top) and ^{13}C-NMR (bottom) spectra of n-octadecanoic acid, a compound from *Euphorbia uralensis*......37
Figure S21. ^{1}H-NMR (top) and ^{13}C-NMR (bottom) spectra of p-hydroxybenzaldehyde, a compound from *Euphorbia uralensis*......38
Figure S22. ^{1}H-NMR (top) and ^{13}C-NMR (bottom) spectra of naringenin, a compound from *Euphorbia uralensis*......39
Figure S23. ^{1}H-NMR (top) and ^{13}C-NMR (bottom) spectra of glycyrrhetinic acid, a compound from *Euphorbia uralensis*......40
Figure S24. ^{1}H-NMR (top) and ^{13}C-NMR (bottom) spectra of punigratine, a compound from *Euphorbia uralensis*......41
Figure S25. GC-MS total ion chromatogram of petroleum ether fraction of *Euphorbia uralensis*.42
Figure S26. Hydrogen bonding frequencies between small molecules and proteins.42

Table S1. Names and types of compounds obtained by isolation from *Euphorbia uralensis*

Number	Compound name	Type of Compounds
EUD-1***	3*β*-(2”-dehydroxy-3”-methyl-L-rha)-12*β*-[(3’,4’-trimethyl-1-oxo -2-pentenyl)oxy]-17*α*-acetyl-8*β*,14*β*-dihydroxypregn-10*β*,13*β*-di methyl-9*α*-hydrogen-5-en-20-one	Steroid
EUD-2**	(+)-loliolide	Sesquiterpenoids
EUD-3**	ginsinsene	Sesquiterpenoids
EUD-4**	Betulin	Triterpenoids
EUD-5**	(24R) -24-Ethylcholest-4-en-3one	Triterpenoids
EUD-6**	(-)-*β*-Sitosterol	Triterpenoids
EUD-7**	Oleanolic acid	Triterpenoids
EUD-8**	Dibutyl phthalate	Aromatics
EUD-9**	Auriculatum A	Aromatics
EUD-10**	di-(2-ethyl)hexyl phthalate	Aromatics
EUD-11**	n-tetradecanoic acid	Fatty acid
EUD-12**	2,6,10,14-tetramethyl-18-butanecarboxy-methylene-henecos-12-en-17*β*-ol	Terpenoids
EUD-13**	n-octadecanoic acid	Fatty acid
EUD-14**	p-hydroxybenzaldehyde	Aromatics
EUD-15**	Naringenin	Flavonoids
EUD-16**	glycyrrhetinic acid	Flavonoids
EUD-17**	punigratane	Alkaloids

(Note : *** denotes a new compound, ** denotes the first isolation from this plant)

1. Structural Analysis of Known Compounds

pubinernoid A: White amorphous powder with the molecular formula: $C_{11}H_{16}O_3$. Comprehensive analysis of ^{1}H-NMR (chloroform-d, 400 Hz) and ^{13}C-NMR (chloroform-d, 100 Hz) data revealed the following characteristic

signals: ^{1}H-NMR (chloroform-d, 400 Hz) showed three methyl hydrogen proton signals at δ_H 1.28 (s, 3H), δ_H 1.47 (s, 3H), and δ_H 1.78 (s, 3H); one hydrogen proton signal at C-2: δ_H 4.32 (s, 1H); and one olefin hydrogen proton signal at δ_H 5.69 (s, 1H). ^{1}H-NMR (chloroform-d, 400 Hz) data: δ_H 5.69 (s, 1H), δ_H 4.33 (p, J=3.6 Hz, 1H), δ_H 2.47 (dt, J=14.1, 2.6 Hz, 1H), δ_H 1.99 (dt, J=14.5, 2.6 Hz, 1H), δ_H 1.92 (s, 1H), δ_H 1.79 (s, 4H), δ_H 1.53 (dd, J=14.6, 3.7 Hz, 1H), δ_H 1.47 (s, 3H), and δ_H 1.27 (s, 3H). ^{13}C-NMR (chloroform-d, 100 Hz) spectral data are shown in Table S2, and the spectrum is shown in Figure S9. After comparing with the literature, the compound was identified as pubinernoid A.

Table S2. ^{13}C-NMR Signal of Compound pubinernoid A

^{13}C	Measurement value	Literature value
1	182.7	183.2
2	172.0	171.6
3	112.8	113
4	86.8	86.6
5	66.7	66.8
6	47.3	47.4
7	45.6	45.7
8	35.9	35.9
9	30.7	30.6
10	27.0	27
11	26.5	26.5

ginsinsene: White powder, molecular formula: $C_{15}H_{24}$. After TLC development, there are dark spots under 254nm. ^{1}H-NMR (chloroform-d, 400 Hz) shows three singlet methyl hydrogen signals at δ_H 1.10 (s, 3H), δ_H 1.39 (s, 3H), and δ_H 1.55 (s, 3H); one doublet methyl hydrogen signal at δ_H 0.83 (s, 3H); ^{13}C-NMR (chloroform-d, 100 Hz) gives two doublet quaternary carbon signals at δ_C 181.3 (C-2) and δ_C 150.32 (C-3) and one low-field quaternary carbon signal at δ_C 62.79 (C-6). ^{1}H-NMR (chloroform-d, 400 Hz) data: δ_H 0.87 (3H, d, J=6.61 Hz), δ_H 0.96 (d, J=12.9 Hz, 1H), δ_H 1.10 (s, 3H), δ_H 1.25 (s, 3H), δ_H 1.20 (m, 1H), δ_H 1.39 (m, 1H), δ_H 1.45 (m, 1H), δ_H 1.61 (m, 1H), δ_H 1.62 (m, 1H), δ_H 1.68 (s, 3H), δ_H 1.73 (m, 1H), δ_H 1.93 (t, 1H), δ_H 2.00 (m, 1H), δ_H 2.37 (m, 1H); ^{13}C-NMR (chloroform-d, 100 Hz) spectral data are shown in Table S3, and the spectrum is shown in Figure S10. After comparing with the literature,

the compound is identified as ginsinsene.

Table S3. ^{13}C-NMR Signal of Compound ginsinsene

^{13}C	Measurement value	Literature value	^{13}C	Measurement value	Literature value
1	44.1	44.7	9	29.7	28.9
2	-	119.9	10	41.2	42
3	150.3	151.2	11	45.7	46
4	40.1	40.1	12	14.1	14
5	34.4	34.2	13	34.3	33.7
6	62.8	61.1	14	22.7	23.6
7	39.8	39.8	15	16.6	17.3
8	29.7	29.5			

Betulin: White solid, molecular formula: $C_{30}H_{50}O_2$. It is easily soluble in petroleum ether and dichloromethane, with a melting point of 256-257 ℃. ^{1}H-NMR (chloroform-d, 400 Hz) shows six methyl hydrogen proton signals: δ_H 0.76 (s, 3H), δ_H 0.86 (s, 3H), δ_H 0.97 (s, 3H), δ_H 1.02 (s, 3H), δ_H 1.07 (s, 3H); two hydroxyl hydrogen proton signals: δ_H 4.56 (dd, J=2.6, 1.4 Hz, 1H), δ_H 4.70 (d, J=2.5 Hz, 1H); other ^{1}H-NMR data: δ_H 3.12 (dd, J=10.8, 5.3 Hz, 1H); δ_H 3.29 (d, J=10.7 Hz, 1H); δ_H 3.74 (dd, J=10.7, 1.9 Hz, 1H). ^{13}C-NMR (chloroform-d, 100 Hz) spectral data indicate a total of 30 carbon atoms, suggesting that the compound has a triterpene parent nucleus structure. The ^{13}C-NMR (chloroform-d, 100 Hz) spectral data are shown in Table S4. The spectral diagram of the compound is shown in Figure S11. After comparing with the literature, the compound is identified as Betulin.

Table S4. ^{13}C-NMR Signal of Compound Betulin

^{13}C	Measurement value	Literature value	^{13}C	Measurement value	Literature value
25	150.8	150.5	7	34.2	33.9
30	109.1	109.6	21	33.9	33.9
27	77.6	77.61	20	29.7	29.5

3	58.8	59.5	16	29.2	29.0
5	55.4	54.9	2	27.9	27.9
9	50.4	49.5	15	27.4	27.5
18	48.6	48.5	12	27	27.0
19	47.9	47.7	23	25.3	24.9
17	47.8	47.6	22	20.7	20.5
14	42.5	42.6	26	18.4	19.0
8	40.9	40.8	11	18.2	17.9
4	38.7	38.9	25	15.7	16.0
1	38.6	38.5	6	15.5	16.0
13	37.3	37.5	24	15.2	15.6
10	37.0	36.9	26	14.2	14.6

(24R)-24-stigmast-4-en-3-one: White powder, $C_{29}H_{48}O$, soluble in organic solvents such as petroleum ether and dichloromethane; ^{1}H-NMR (chloroform-d, 400 Hz) shows a set of exocyclic olefin proton signals at δ_H 5.70 (s, 1H); three sets of methyl proton signals at C_{21}, C_{27}, C_{29}: δ_H 0.85-0.78 (m, J=7.8 Hz, 9H); two hydrogen proton signals connected to the carbonyl group: δ_H 2.41-2.33 (m, 2H). The remaining hydrogen proton signals are: δ_H 2.30-2.22 (m, 1H), δ_H 2.03-1.97 (m, 2H), δ_H 1.87-1.78 (m, 2H), δ_H 1.71-1.59 (m, 3H), δ_H 1.56-1.44 (m, 5H), δ_H 1.29-1.21 (m, J=8.4 Hz, 6H), δ_H 1.16 (s, 3H), δ_H 1.14-0.97 (m, 7H), δ_H 0.95 (d, J=7.4 Hz, 2H), δ_H 0.90 (d, J=6.5 Hz, 3H), δ_H 0.85-0.78(m, 9H), δ_H 0.69 (s, 3H). The ^{13}C-NMR (chloroform-d 100 Hz) spectral data are shown in Table S5, and the spectral diagram is shown in Figure S12. After comparison with reference literature, the compound was identified as (24R)-24-stigmast-4-en-3-one.

Table S5. ^{13}C-NMR Signal of Compound (24R)-24-stigmast-4-en-3-one

^{13}C	Literature value	Measurement value	^{13}C	Literature value	Measurement value
1	35.2	35.8	16	25.9	24.4
2	34.1	34.1	17	56.2	56.1
3	198.9	199.9	18	12	12

4	124	123.9	19	19	19.2
5	170.2	171.9	20	36.1	36.3
6	32.5	32.3	21	19.4	18.9
7	31.6	34.2	22	33.9	33.2
8	35.7	35.9	23	26.4	28.4
9	53.7	54.0	24	46.1	46.0
10	38.6	38.8	25	30.4	29.9
11	21.1	21.4	26	21	21.2
12	39.8	39.8	27	21	20.0
13	42.6	42.6	28	23.2	23.3
14	56.5	56.2	29	12.2	12.2
15	26.2	26.3			

β-sitosterol: White needle-like crystal, molecular formula: $C_{29}H_{50}O$. It is easily soluble in chloroform, slightly soluble in ethanol or acetone, and insoluble in water. The reaction between acetic anhydride and concentrated sulfuric acid is positive, suggesting that the compound may be a terpene or sterol. ^{1}H-NMR (chloroform-d, 400 Hz) shows δ_H 5.42 (m, 1H), indicating that the compound contains an olefinic hydrogen (C_6 position) signal, with complex SP^3 hybridized carbon signals between δ_H 0.5-2.5, and the remaining hydrogen proton signals are: δ_H 3.63 (m, 1H), δ_H 1.013 (s, 3H); δ_H 0.92 (d, J=6.7 Hz, 4H); δ_H 0.88-0.73 (m, 9H); δ_H 0.68 (s, 3H); ^{13}C-NMR (chloroform-d, 100 Hz) spectral data are shown in Table S6, and the spectral diagram is shown in Figure S13. After comparison with the literature, the compound was identified as *β*-sitosterol.

Table S6. ^{13}C-NMR Signal of Compound *β*-sitosterol

^{13}C	Literature value	Measurement value	^{13}C	Measurement value	Literature value
1	37.3	37.3	16	28.3	28.2
2	31.5	31.9	17	56.1	56
3	71.5	71.8	18	11.9	11.8
4	42.1	42.3	19	19.4	19.3
5	140.6	140.8	20	36.2	36.1
6	121.7	121.7	21	18.8	18.7
7	31.8	31.6	22	33.9	33.9

8	31.9	31.9	23	26.1	26
9	50.2	50.1	24	45.8	45.8
10	36.4	36.5	25	29.2	29.1
11	21	21.1	26	19.8	19.8
12	39.7	39.8	27	19.0	19
13	42.3	42.3	28	23.1	23
14	56.7	56.8	29	11.9	11.9
15	24.2	24.3			

Oleanolic acid: White amorphous powder with the molecular formula of $C_{30}H_{48}O_3$, easily soluble in petroleum ether and dichloromethane. The reaction of acetic anhydride with concentrated sulfuric acid yields purple-red spots, indicating that the compound may be a terpene or a sterol. ^{1}H-NMR (chloroform-d, 400 Hz) shows seven methyl hydrogen proton signals: δ_H 0.76 (s, 3H), δ_H 0.78 (s, 3H), δ_H 0.90 (s, 3H), δ_H 0.91 (s, 3H), δ_H 0.93 (s, 3H), δ_H 0.99 (s, 3H), δ_H 1.14 (s, 4H); one cyclic olefin hydrogen proton signal: 5.29 (t, 1H); the remaining hydrogen proton signals are: 3.22 (dd, J=11.2, 4.7 Hz, 1H); ^{13}C-NMR (chloroform-d, 100 Hz) spectral data are shown in Table S7, and the compound's spectral diagram is shown in Figure S14. After comparison with the literature, the compound is identified as Oleanolic acid.

Table S7. ^{13}C-NMR Signal of Compound Oleanolic acid

^{13}C	Measurement value	Literature value	^{13}C	Measurement value	Literature value
28	180.5	181.9	7	32.6	32.5
13	143.1	143.9	22	32.4	32.4
12	122.7	122.2	20	30.7	30.6
3	79.0	78.9	2	28.1	28.5
14	55.2	55.1	30	27.7	28
5	48.3	47.6	29	27.0	27.6
9	47.6	47.6	27	26.8	26.9
17	46.5	46.5	15	25.9	25.8
19	45.9	46	16	23.7	23.5
18	41.6	41.1	11	23.6	23.3
8	39.3	38.6	24	23.4	23
4	38.8	38.3	23	17.4	18.2

1	37.1	37	6	17.1	16.9
10	33.8	33.8	25	15.5	15.5
21	33.5	33	26	15.3	15.2

Dibutyl phthalate: A pale yellow oil with the molecular formula $C_{16}H_{22}O_4$. After TLC development, there are distinct fluorescent spots visible at 254nm. It is easily soluble in solvents such as petroleum ether, dichloromethane, and acetone, and is miscible with most hydrocarbons. It is insoluble in water and exhibits low water solubility and volatility. ^{1}H-NMR (chloroform-d, 400 Hz) data: δ_H 7.65 (dd, J=5.8, 3.3 Hz, 2H), δ_H 7.50-7.42 (m, 2H), δ_H 4.24 (t, 4H), δ_H 1.68-1.61(m, 4H), δ_H 1.42-1.34 (m, 4H), δ_H 0.89 (t, 6H). ^{13}C-NMR (chloroform-d, 100 Hz) spectral data are shown in Table S8, and the spectrum is shown in Figure S15. Through literature review, the compound was identified as Dibutyl phthalate.

Table S8. ^{13}C-NMR Signal of Compound Dibutyl phthalate

^{13}C	Literature value	Measurement value
7，7'	167.7	167.7
1,2	132.3	132.3
4,5	130.9	130.9
3,6	128.8	128.9
8,8'	65.6	65.6
9,9'	30.6	30.6
10,10'	19.2	19.2
11,11'	13.7	13.8

Auriculatum A: A pale yellow oil with the molecular formula $C_{20}H_{30}O_4$. After TLC development, distinct fluorescent spots are visible at 254nm. It is easily soluble in solvents such as petroleum ether and dichloromethane, but insoluble in water. The ^{1}H-NMR data (chloroform-d, 400 Hz) are as follows: δ_H 8.10 (s, 2H), δ_H 7.72 (m, 1H), δ_H 7.54 (m, 1H), δ_H 4.26 (m, 2H), δ_H 1.68 (m, 2H), δ_H 4.31 (t, 1H), δ_H 1.43 (m, 2H), δ_H 1.35 (m, 2H), δ_H 1.28 (m, 2H), δ_H 0.89 (m, 3H). The ^{13}C-NMR data (chloroform-d, 100 Hz) are shown in Table S9, and the spectrum is depicted in Figure S16. After

comprehensive comparison with literature data, the compound is identified as Auriculatum A.

Table S9. ^{13}C-NMR Signal of Compound Auriculatum A

^{13}C	Literature value	Measurement value	^{13}C	Literature value	Measurement value
7	166.9	167.7	9	29.9	30.5
7'	166.8	165.9	9'	29.7	29.7
1	131.6	134.2	11	28.2	28.9
4	131.6	132.3	11'	23.1	23.9
6	131.5	131.9	12	22.3	22.9
5	131.4	130.9	12'	18.5	19.1
3	128.5	129.5	14	13.8	14.0
2	67.3	67.7	13	13.4	13.7
8	64.9	65.5	13'	10.7	11.1

Di-(2-ethylhexyl) phthalate: A pale yellow oil with the molecular formula $C_{24}H_{38}O_4$. After TLC development, it exhibits distinct fluorescent spots at 254 nm. It is easily soluble in solvents such as petroleum ether, dichloromethane, and acetone, and is miscible with most hydrocarbons but insoluble in water. The ^1H-NMR data (chloroform-d, 400 Hz) are as follows: δ_H 7.71 (dd, J=5.6, 2H), δ_H 7.53 (dd, J=5.6, 2H), δ_H 4.56-4.05 (m, 4H), δ_H 1.74-1.68 (m, 2H), δ_H 1.38-1.52 (m, 16H), δ_H 1.03 (m, 6H), δ_H 0.97 (t, 6H). The ^{13}C-NMR data (chloroform-d, 100 Hz) are shown in Table S10, and the spectrum is depicted in Figure S17. Upon comparison with the literature, the compound is identified as di-(2-ethyl)hexyl phthalate.

Table S10. ^{13}C-NMR signals of the compound di-(2-ethyl)hexyl phthalate

^{13}C	Measurement value	Literature value
-	167.7	167.9
1,2	132.3	132.5
3,6	130.9	131.1

4,5	128.8	129.0
1',1''	65.6	65.7
2',2''	31.9	32.1
3',3''	30.6	30.7
4',4''	29.7	29.9
5',5''	29.7	29.5
6',6''	22.7	22. 9
a',a'	19.2	19.3
7',7''	14.1	14.3
b',b'	13.7	13.9

n-tetracosanoic acid: A pale yellow oily substance with the molecular formula $C_{24}H_{48}O_2$. It is easily soluble in petroleum ether, dichloromethane, chloroform, and other solvents, and hardly soluble in methanol. Thin layer chromatography shows no dark spots under ultraviolet light. ^{1}H-NMR (chloroform-d, 400 Hz) displays a methyl proton signal at δ_H 0.91-0.82 (m, J=6.8 Hz, 3H); a broad strong peak integrating 18 hydrogens at δ_H 1.32 (d, J=18.8 Hz, 40H); ^{13}C-NMR (chloroform-d, 100 Hz) spectral data are shown in Table S11, and the spectrum is shown in Figure S18. After comparing with the literature, the compound was identified as n-tetradecanoic acid.

Table S11. ^{13}C-NMR Signal of Compound n-tetradecanoic acid

^{13}C	Literature value	Measurement value
1	178.4	177.5
2	34.0	33.3
22	31.9	31.7
21	29.0	29.1
5	29.3	29.3
6-20	29.6	29.5
3	24.7	24.8

23	22.7	22.4
24	14.1	13.5

2,6,10,14-tetramethyl-18-butanecarboxymethyl-12-en-17*β*-ol: Yellow solid, mo lecular formula: $C_{29}H_{56}O_3$. ^{1}H-NMR (chloroform-d, 400 Hz) shows two olefin pro ton signals: δ_H 7.71 (td, 3.3 Hz, 1H), δ_H 7.56-7.50 (m, 1H); one hydrogen signal connected to C-17 position: δ_H 4.31 (t, 1H); one methylene signal directly connec ted to the oxygen atom: δ_H 4.22 (qd, J=10.9, 5.9 Hz, 2H); the remaining hydroge n proton signals are: δ_H 2.04 (s, 1H), δ_H 1.70 (d, J=6.8 Hz, 2H), δ_H 1.68 (d, J=5. 9 Hz, 2H), δ_H 1.66 (d, J=7.5 Hz, 2H), δ_H 1.51 (d, J=6.9 Hz, 2H), δ_H 1.47 (d, J= 7.5 Hz, 2H), δ_H 1.46-1.43 (m, 4H), δ_H 1.42-1.41 (m, 2H), δ_H 1.40 (s, 2H), δ_H 1.3 8 (d, J=8.5 Hz, 2H), δ_H 1.34 (d, J=10.2 Hz, 8H), δ_H 1.32 (s, 2H), δ_H 1.28 (s, 10 H), δ_H 1.15 (s, 6H), δ_H 1.11-1.06 (m, 6H); ^{13}C-NMR (chloroform-d, 100 Hz) spec tral data are shown in Table S12, and the spectral diagram is shown in Figure S1 9. After comparing with the literature, the compound was identified as 2,6,10,14-tetramethyl-18-butylcarboxymethyl-12-en-17*β*-ol.

Table S12. ^{13}C-NMR signals of the compound 2,6,10,14-tetramethyl-18-butylcarb oxymethyl-12-en-17*β*-ol

^{13}C	Measurement value	Literature value	^{13}C	Measurement value	Literature value
1	14.0	14.2	16	38.6	38.7
2	38.0	37.4	17	68	68.1
3	29.6	29.7	18	30.5	30.04
4	29.4	29.7	19	22.6	22.9
5	29.3	29.4	20	10.9	10.9
6	31.9	31.9	21	13.8	14.1
7	29.1	29.1	22	13.6	13.7
8	29.0	29.0	23	18.5	19.7
9	28.8	28.9	24	19.1	19.1

10	30.3	30.3	25	65.5	65.5
11	27.1	27.9	1'	167.7	167.7
12	128.7	128.8	2'	39.3	39.3
13	130.8	130.9	3'	22.5	22.7
14,15	33.76	34.1	4'	14.0	14.0

n-Octadecanoic acid: White powder, molecular formula: $C_{18}H_{36}O_2$. Soluble in petroleum ether and chloroform, almost insoluble in water. ^{1}H-NMR (chloroform-d, 400 Hz) shows one methylene signal: δ_H 2.29 (d, J=7.5 Hz, 2H), δ_H 1.63 (m, 1H); one methyl signal: δ_H 0.81 (t, 3H); multiple overlapping methylene signals δ_H 1.19-1.22; ^{13}C-NMR (chloroform-d, 100 Hz) spectral data are shown in Table S13. The compound's spectral diagram is shown in Figure S20. After comparing with the literature, the compound was identified as n-octadecanoic acid.

Table S13. ^{13}C-NMR Signal of Compound n-octadecanoic acid

^{13}C	Literature value	Measurement value
1	179.8	178.9
2	33.9	33.4
3	31.9	32.2
4-15	29.7-29.0	28.7-28.3
16	24.7	23.7
17	22.7	21.7
18	14.1	13.1

p-hydroxybenzaldehyde : White powder (petroleum ether-acetone), molecular formula: $C_7H_6O_2$. After TLC development, there are obvious fluorescent spots under 254 nm. After coloration with 10% concentrated sulfuric acid and heating, it appears purplish red. ^{1}H-NMR (chloroform-d, 400 Hz) shows a signal of aldehyde hydrogen δ_H 9.86 (s, 1H); four signals of methine hydrogen δ_H 7.8 (d, J=8.6 Hz, 2H), δ_H 7.03 (d, J=8.6 Hz, 2H). ^{13}C-NMR (chloroform-d, 100 Hz) spectral data are shown in Table S14,

and the spectrum is shown in Figure S21. After comparing with the literature, it is determined that the compound is p-hydroxybenzaldehyde.

Table S14. ^{13}C-NMR Signal of Compound p-hydroxybenzaldehyde

^{13}C	Measurement value	Literature value
1	191.9	191.0
2	164.7	164.3
3	133.7	132.8
4	131.4	130.5
5	129.6	129.5
6	117.5	116.4

Naringenin: Yellow amorphous powder, molecular formula: $C_{15}H_{12}O_5$. It is easily soluble in chloroform, acetone, and methanol. ^{1}H-NMR (chloroform-d, 400 Hz) shows one oxygen-linked methine hydrogen: δ_H 5.32 (dd, J=3.0, 12.9 Hz, 1H); δ_H 7.44-7.36 (m, 2H), δ_H 6.94-6.86 (m, 2H), δ_H 5.96 (q, J=2.2 Hz, 2H), δ_H 5.46 (dd, J=12.9, 3.0 Hz, 1H) indicating an aromatic ring structure; one methylene hydrogen signal: 3.19 (dd, J=17.1, 12.9 Hz, 1H), 2.73 (dd, J=17.1, 3.0 Hz, 1H); ^{13}C-NMR (chloroform-d, 100 Hz) spectral data are shown in Table S15, and the compound spectrum is shown in Figure S22. After comparing with the literature, the compound was identified as naringenin.

Table S15. ^{13}C-NMR signal of compound naringenin

^{13}C	Literature value	Measurement value	^{13}C	Literature value	Measurement value
1	164.9	166.4	8	43	42.7
2	95.1	95.9	9	82.5	79.1
3	163.8	164.4	10	130.9	129.9
4	102.8	102.3	11	127.4	128.2
5	163.6	164.1	12, 14	116.1	115.2

6	94.6	95.8	13	157.4	157.8
7	196.8	196.3			

glycyrrhetinic acid: Yellow amorphous powder, molecular formula: $C_{15}H_{12}O_4$. Soluble in organic solvents such as methanol, ethanol, DMSO, etc. ^{1}H-NMR (chloroform-d, 400 Hz) shows one oxygen-linked methine hydrogen: δ_H 5.45 (dd, J=13.0, 2.9 Hz, 1H); δ_H 7.73 (d, J=8.6 Hz, 1H), δ_H 7.42-7.38 (m, 2H), δ_H 6.92-6.88 (m, 2H), δ_H 6.58 (dd, J=8.6, 2.3 Hz, 1H), δ_H 6.42 (d, J=2.3 Hz, 1H), indicating that the compound has an aromatic structure, and one methylene hydrogen signal: δ_H 3.05 (dd, J=16.7, 13.0 Hz, 1H), δ_H 2.68 (dd, J=16.8, 2.9 Hz, 1H); ^{13}C-NMR (chloroform-d, 100 Hz) spectral data are shown in Table S16, and the compound's spectral diagram is shown in Figure S23. After comparing with the literature, the compound was identified as glycyrrhetinic acid.

Table S16. ^{13}C-NMR Signal of Compound glycyrrhetinic acid

^{13}C	Literature value	Measurement value
1	163.5	164.5
2	107.4	111.2
3	130.8	129.
4	114.3	115.2
5	164	165.1
6	102	103.6
7	190.9	190.5
8	42.7	44.6
9	82.5	80.5
10	130.9	131.2
11, 15	127.4	128.9
12, 14	116.1	116.1
13	157.4	158.5

punigratine: light yellow powder, molecular formula: $C_{19}H_{39}N$. ^{1}H-NMR (chloroform-d, 400 Hz) shows three terminal methyl hydrogen signals: δ_H 1.04-0.72 (m, 6H), methyl groups attached to nitrogen shift to δ 2.37 (s, 3H), two methylene hydrogen signals: δ_H 1.41 (s, 2H), δ_H 1.58 (s, 2H); two methine hydrogen proton signals: δ_H 2.33 (t, J=12.2 Hz, 2H); ^{13}C-NMR (chloroform-d, 100 Hz) shows only nine signals, indicating that the compound may have a symmetrical structure. No peaks above 60 ppm indicate the absence of aromatic rings. δ_C 14.13 indicates the presence of methyl groups, and δ_C values from 22 to 31 indicate the presence of multiple methylene groups. The methyl group attached to the electronegative nitrogen atom peaks at δ_C 38.16. Carbon spectral data are shown in Table S17, and the compound's spectral diagram is shown in Figure S24. After comparing with the literature, the compound was identified as punigratine.

Table S17. ^{13}C-NMR Signal of Compound punigratine

^{13}C	Literature value	Measurement value
2，5	69.3	63.9
8	39.8	38.1
5', 5''	31.9	31.2
1',1''	31.7	31.0
3',3''	29.6	29.7
4',4''	29.3	29.3
2',2''	27.3	27.2
6',6''	22.7	22.7
7',7''	14.1	14.1

Table S18. Chemical constituents of petroleum ether parts of *Euphorbia uralensis*

Number	Name	Retention Time (min)	Molecular formula	Molecular Weight (m/z)	Percentage Content (%)
1	1-(1,5-dimethyl-4-hexenyl)-4-methylenebicyclo[3.1.0]hexane	10.811	$C_{15}H_{24}$	204.351	0.25
2	6-Methyl tridecane	11.24	$C_{14}H_{30}$	198.388	0.73
3	Caryophyllin	12.235	$C_{15}H_{24}O$	220.35	2.91
4	9(10)-Z-*α*-trans-limonene alcohol	12.585	$C_{15}H_{24}O$	220.35	0.5
5	2-Methyl-6-[(1S)-4-methylcyclohexyl-3-enyl-1-yl]hept-2,6-dien-1-ol	12.717	$C_{15}H_{24}O$	220.35	1.43
6	(E)-2-(6,10-dimethylundeca-1,5,9-trien-2-yl)ethylene oxide	12.829	$C_{15}H_{24}O$	220.35	0.47
7	Atlantic ketone	12.871	$C_{15}H_{24}O$	220.35	0.96
8	dolichodial	12.998	$C_{10}H_{14}O_2$	166.217	0.98
9	(+)-Calamusene B	13.072	$C_{15}H_{24}O$	220.35	6.69
11	Myristic acid	13.586	$C_{14}H_{28}O_2$	228.371	0.83
12	(E)-Atlantone	13.893	$C_{15}H_{22}O$	218.33	2.39
14	3,7,11,15-tetramethylhexen-1-ol	14.354	$C_{20}H_{40}O$	296.531	1.69
15	4-Methylcyclopentadecan-1-one	14.422	$C_{16}H_{30}O$	238.409	1.13
16	3,7,11,15-tetramethylhexen-1-ol	14.761	$C_{20}H_{40}O$	296.531	0.37
17	13-tetradecenic acid	14.984	$C_{14}H_{26}O$	210.356	0.65
18	Palmitic acid methyl ester	15.158	$C_{17}H_{34}O_2$	270.451	12.45
20	palmitic acid	15.471	$C_{16}H_{32}O_2$	256.42	15.86
21	butyl n-decyl phthalate	15.582	$C_{22}H_{34}O_4$	362.503	1.53
22	Ethyl 8-methylnonanoate	15.783	$C_{12}H_{24}O_2$	202.29	0.36

Table S19. Effect of compounds on apoptosis rate of MCF-7/ADR cell injury ($\overline{X}$±s，n=3)

Group	Concentration（μM）	Number of apoptotic cells	Apoptosis rate（%）
Control	-	32.33±2.05	3.23±0.21
Verapamil	55.00	71.67±1.25	7.17±0.12
EUD-1	40.00	55.00±0.82	5.50±0.08
EDU-2	0.80	59.67±0.47	5.97±0.05
EDU-15	0.20	41.67±1.7	4.17±0.17
EDU-17	0.16	64.00±1.63	6.40±0.16

Note: P<0.05 compared to Control group

Table S20. Stable state binding energies and their composition (unit: kJ/mol)

Complex	Verapamil-ABCB1	EUD-17-ABCB1
ΔEvdw	-130.03±1.51	-184.58±0.14
ΔEele	-11.82±0.62	1.65±0.11
ΔEpol	65.97±2.86	68.93±3.95
ΔEnonpol	-16.72±0.04	-25.26±0.58
ΔEMMPBSA	-92.59±2.06	-139.26±3.18
-TΔS	18.73±3.80	15.38±2.35
ΔGbind*	-73.86±5.78	-123.87±4.44

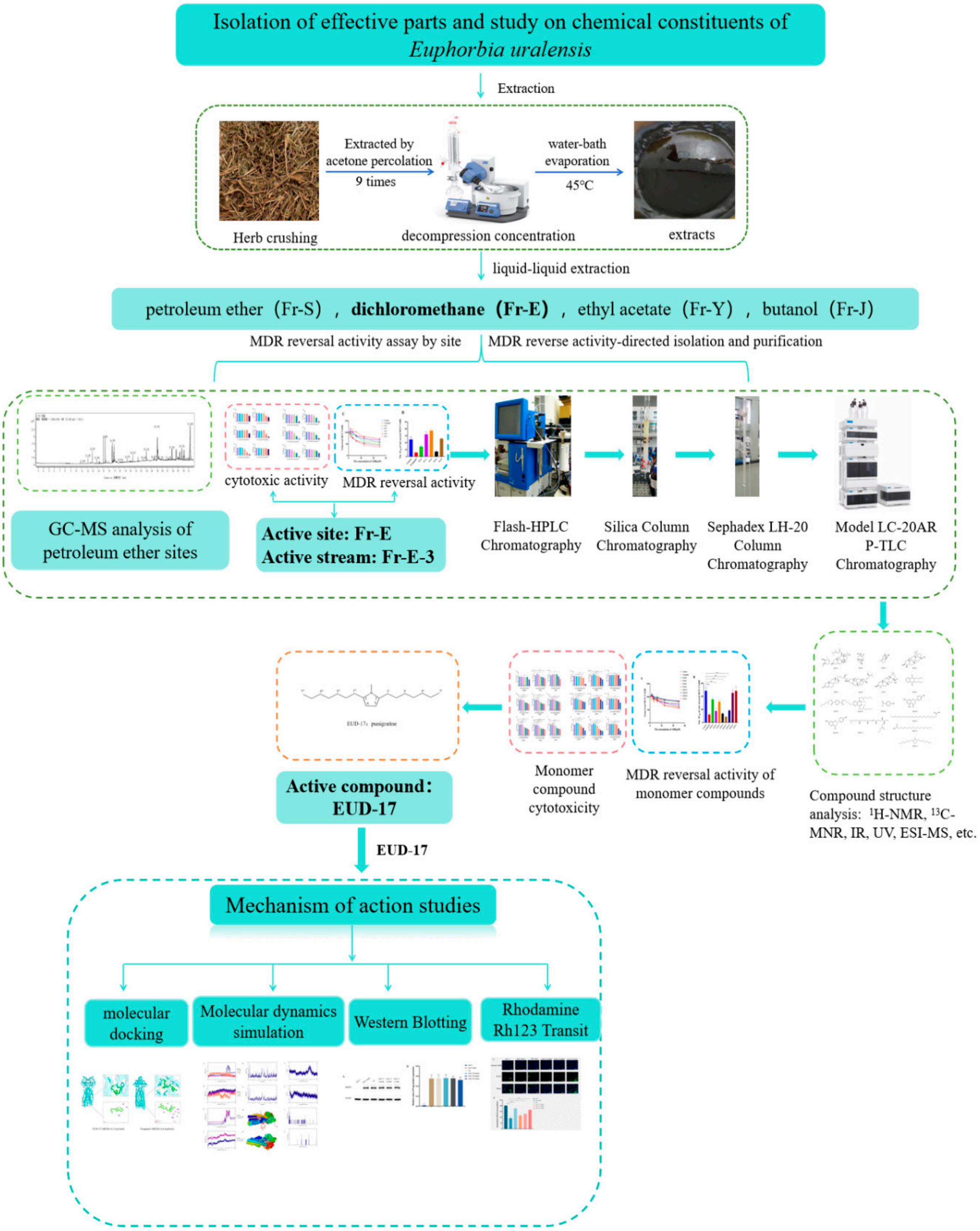

Figure S1. Flowchart for the isolation of effective and study on chemical constituents of *Euphorbia uralensis*.

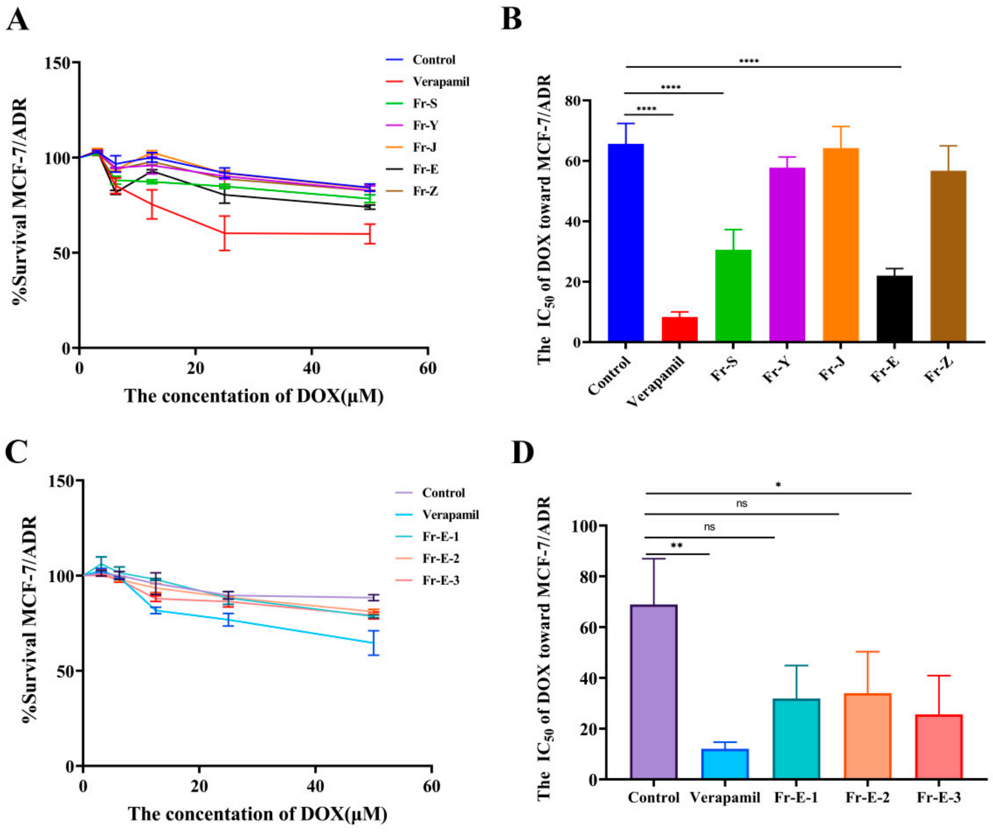

Figure S2. MDR reversal activity of total extracts and extraction sites

(A) Effect of DOX on MCF-7/ADR cell activity after treatment with positive drug Verapamil and extracts of each parts. (B) The IC_{50} value of DOX on MCF-7/ADR cell activity after treatment with Verapamil andand extracts of each parts. (C) Effect of DOX on MCF-7/ADR cell activity after treatment with positive drug verapamil and Fr-E site extract. (D) IC_{50} values of the effect of DOX on MCF-7/ADR cell activity.(n=3) .

Note:*P<0.05 compared to Control (DOX).

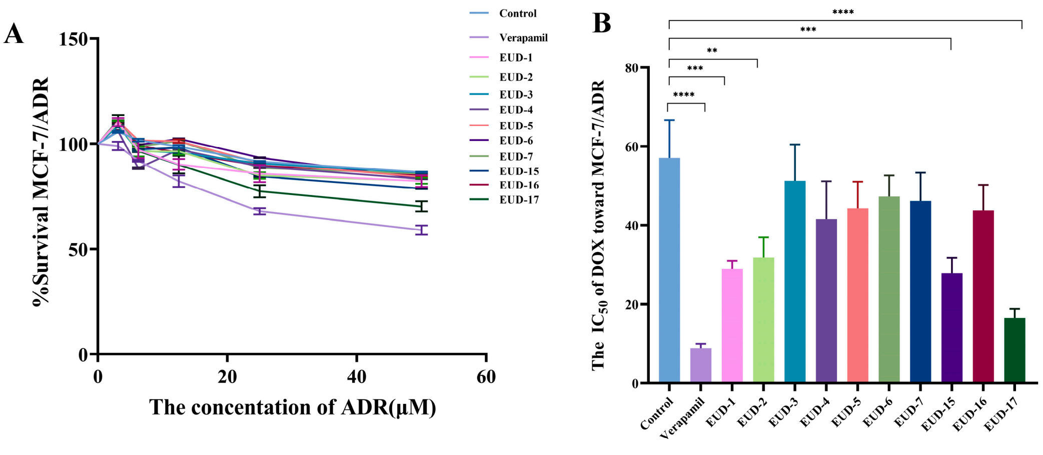

Figure S3. Reversal of MDR activity in MCF-7/ADR cells by compounds (n=3) (A) Cell viability of MCF-7/ADR after compound concomitant with Dox (B) Corresponding IC50 values .

Note:*P<0.05 compared to Control (DOX).

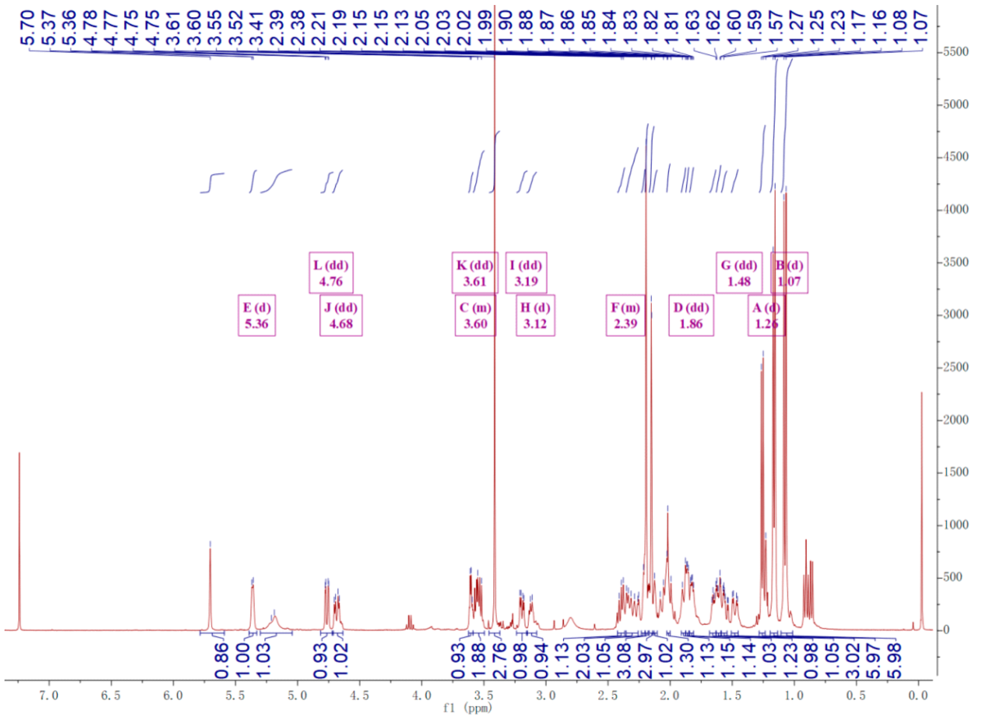

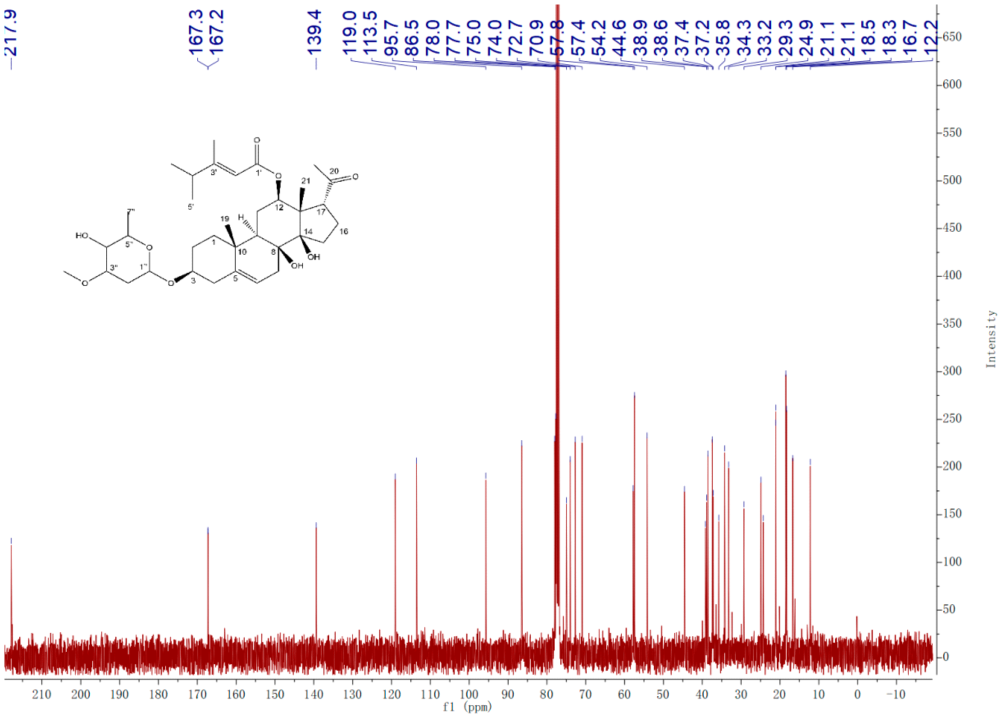

Figure S4. ^{1}H-NMR (top) and ^{13}C-NMR (bottom) spectra of EUD-1, a compound from *Euphorbia uralensis*

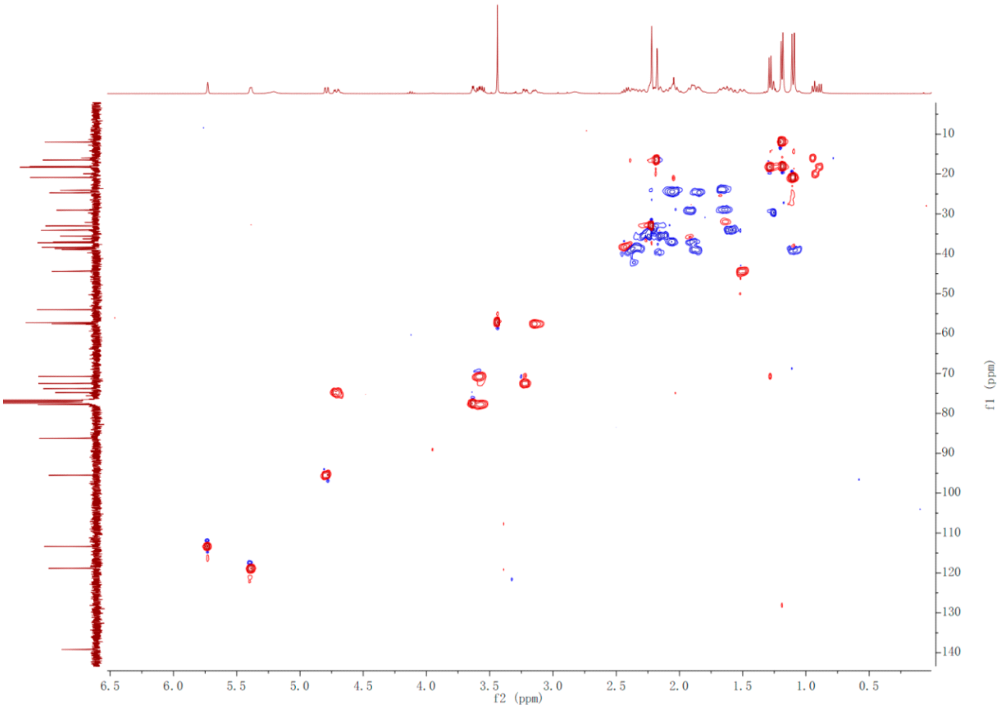

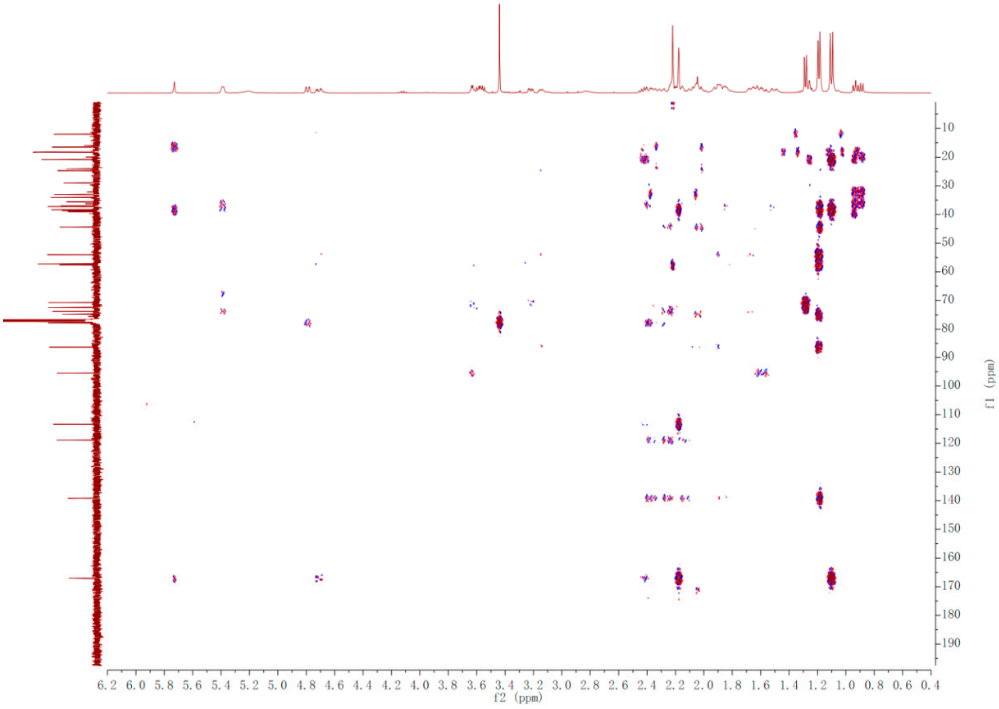

Figure S5. HMQC (top) and HMBC (bottom) spectra of EUD-1 compound from *Euphorbia uralensis*

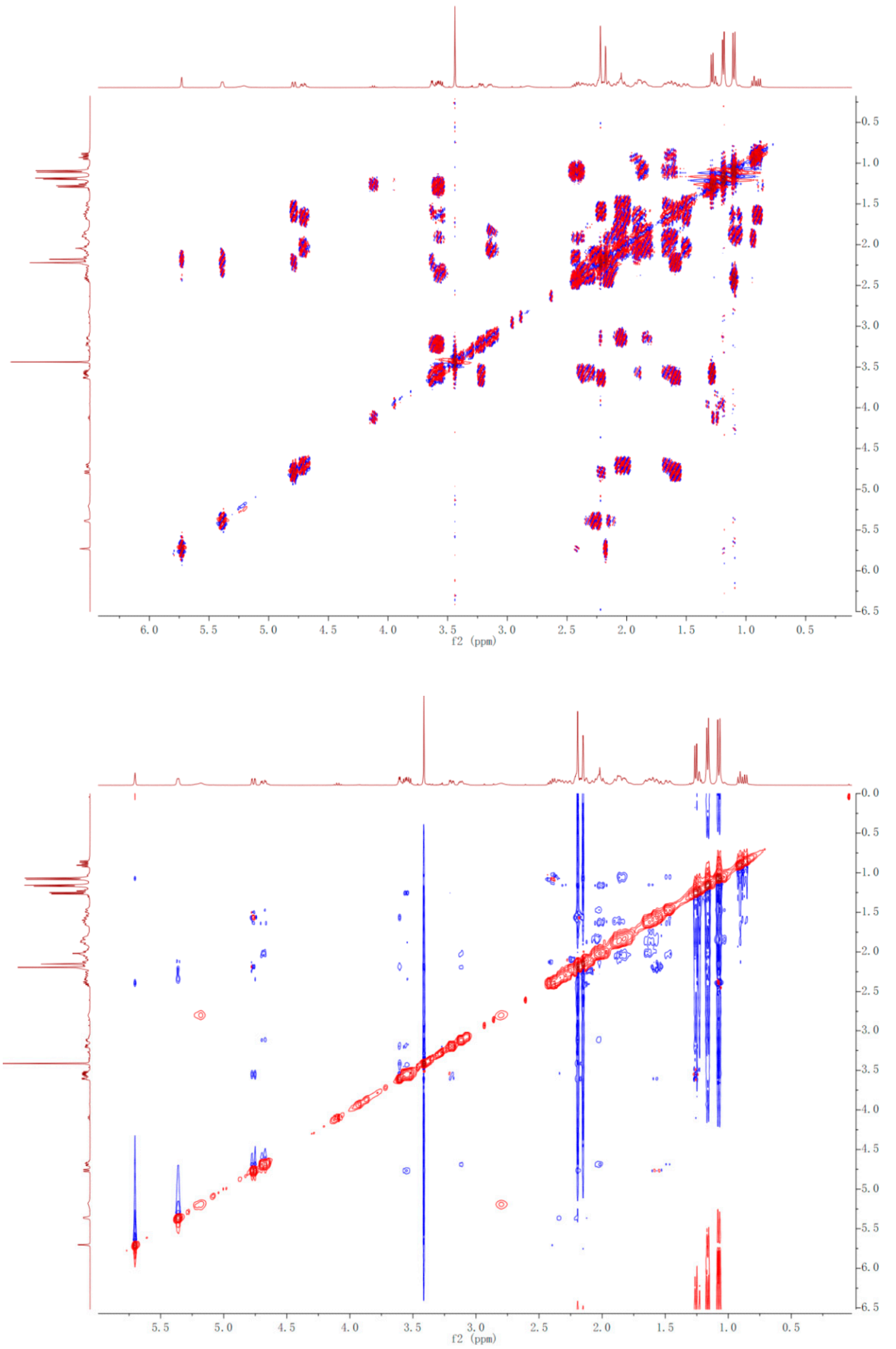

Figure S6. ^{1}H-^{1}H COSY (top) and NOESY (bottom) spectra of EUD-1, a compound from *Euphorbia uralensis*

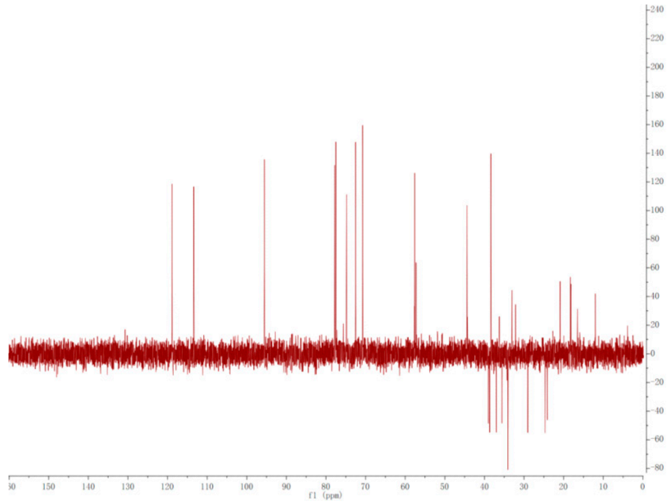

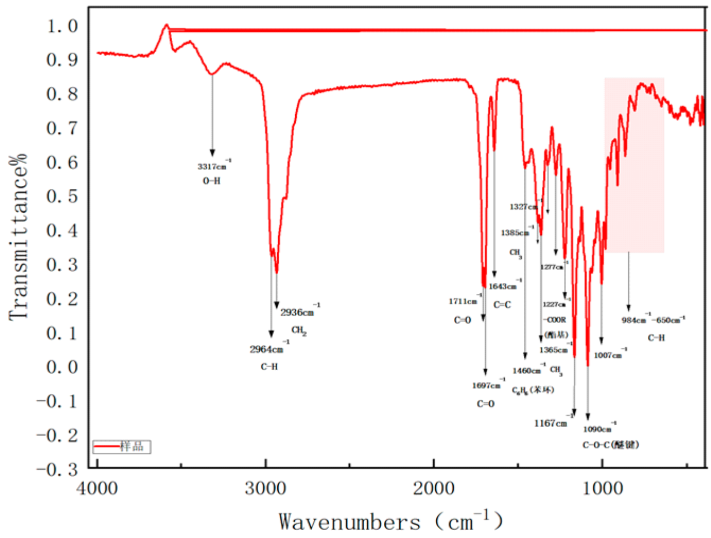

Figure S7. DEPT spectrum (top) and IR spectrum (bottom) of EUD-1 compound from *Euphorbia uralensis*

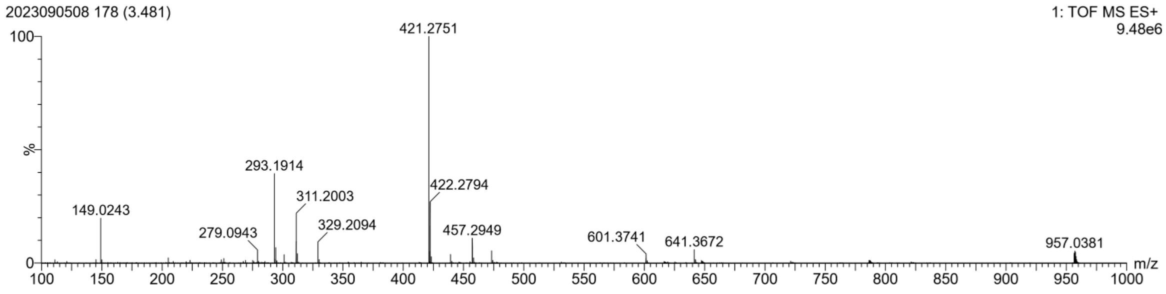

Figure S8. HRESI(+) MS spectrum of EUD-1 compound from *Euphorbia uralensis*

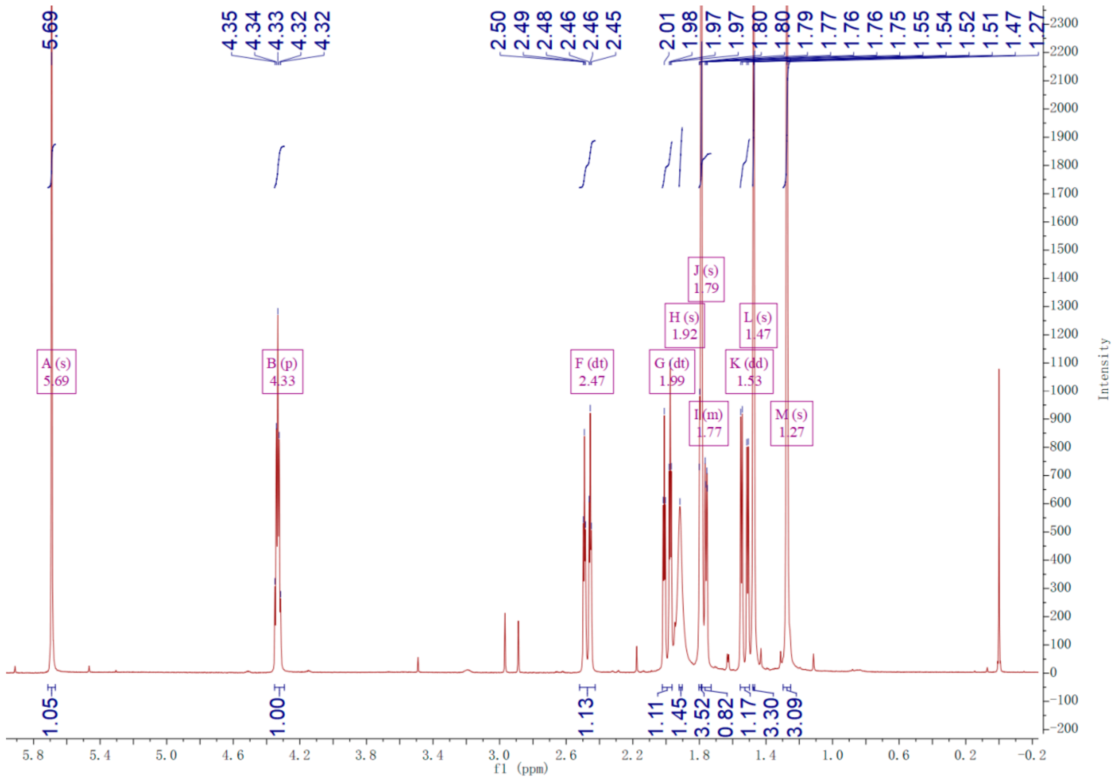

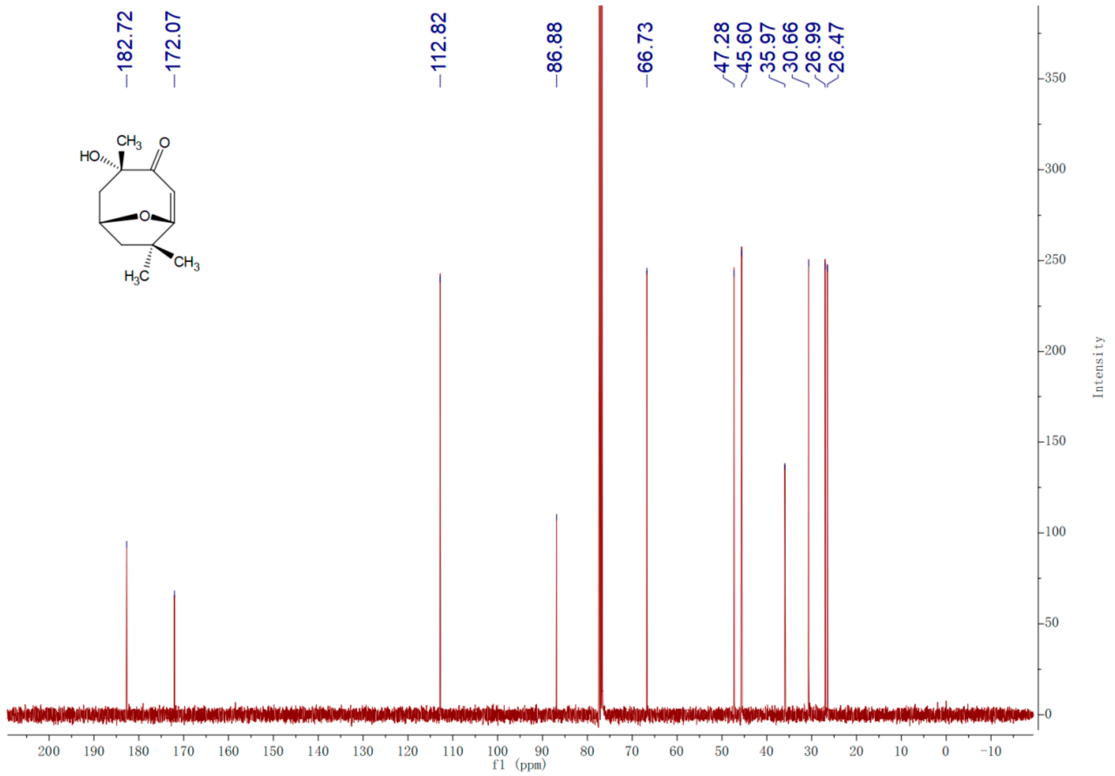

Figure S9. ^{1}H-NMR (top) and ^{13}C-NMR (bottom) spectra of pubinernoid A, a compound from *Euphorbia uralensis*

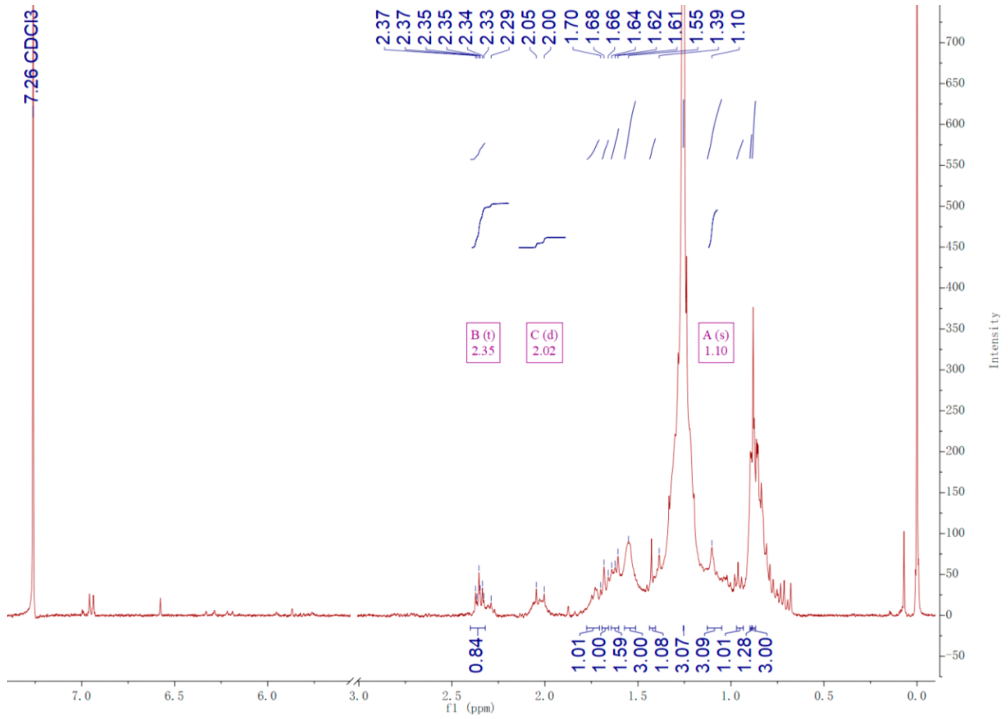

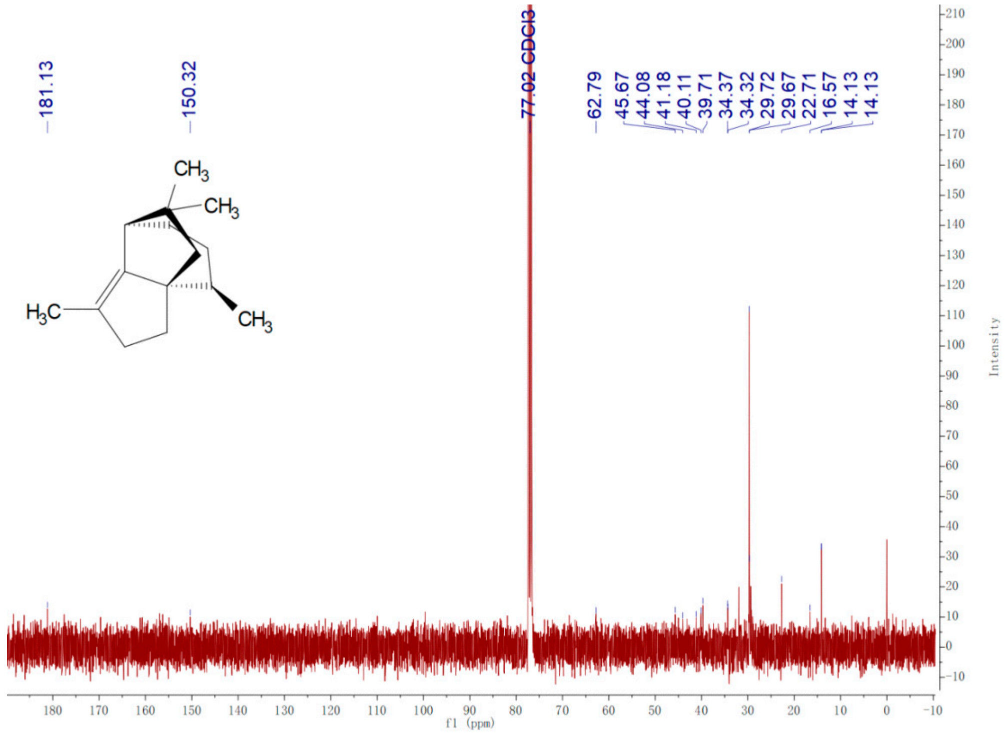

Figure S10. ^{1}H-NMR (top) and ^{13}C-NMR (bottom) spectra of ginsinsene, a compound from *Euphorbia uralensis*

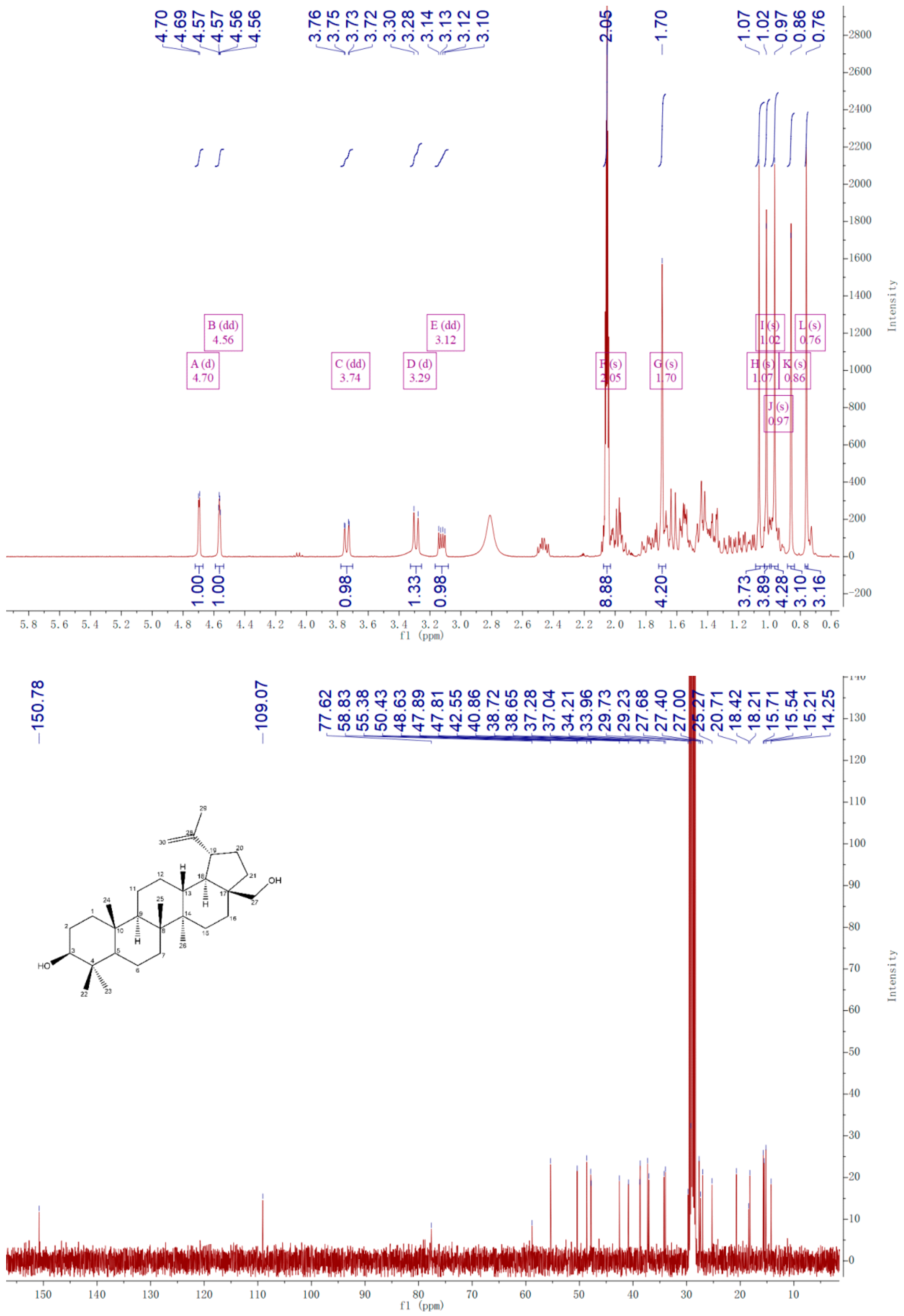

Figure S11. ^{1}H-NMR (top) and ^{13}C-NMR (bottom) spectra of Betulin, a compound from *Euphorbia uralensis*

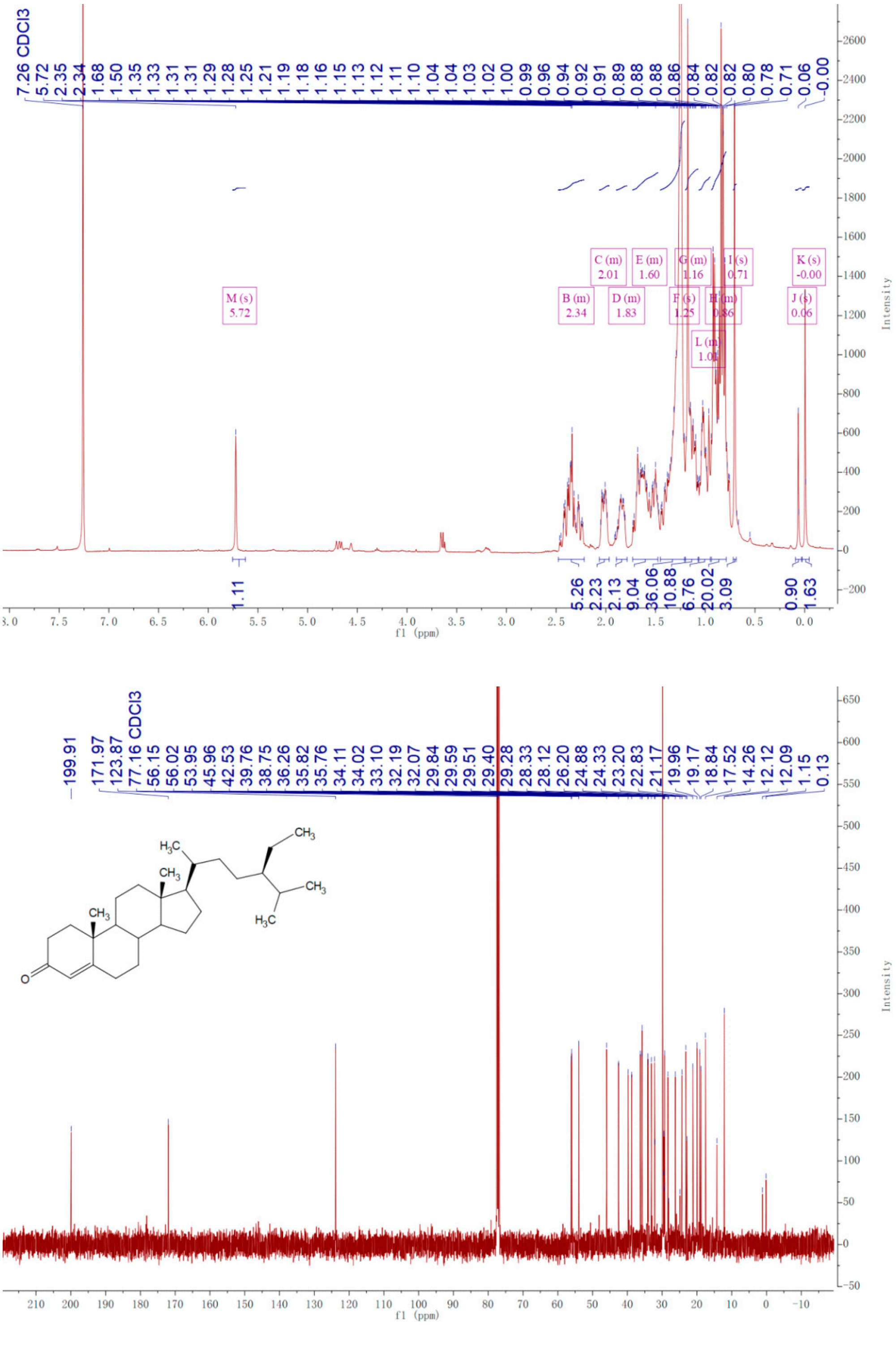

Figure S12. ^{1}H-NMR (top) and ^{13}C-NMR (bottom) spectra of (24R)-24-stigmast-4-en-3-one, a compound from *Euphorbia uralensis*

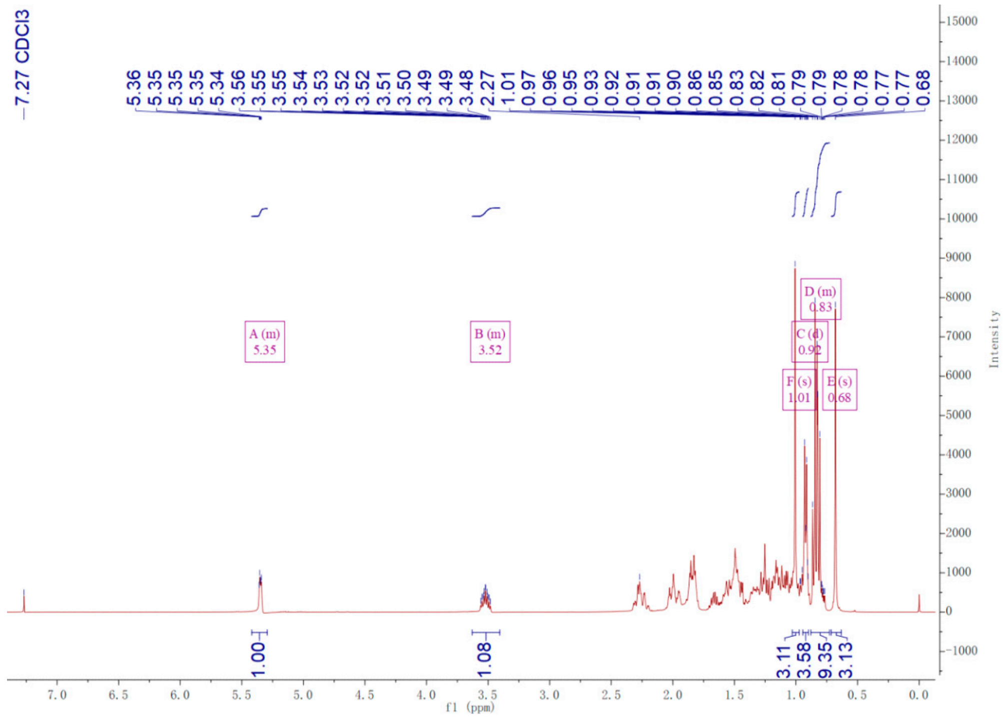

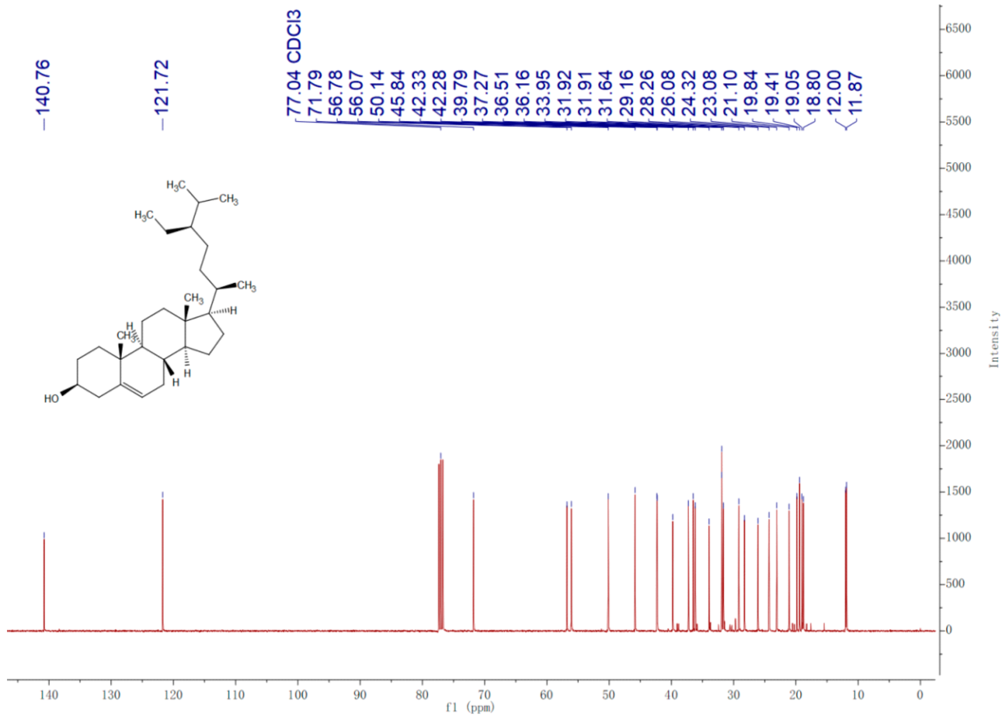

Figure S13. ^{1}H-NMR (top) and ^{13}C-NMR (bottom) spectra of *β*-sitosterol, a compound from *Euphorbia uralensis*

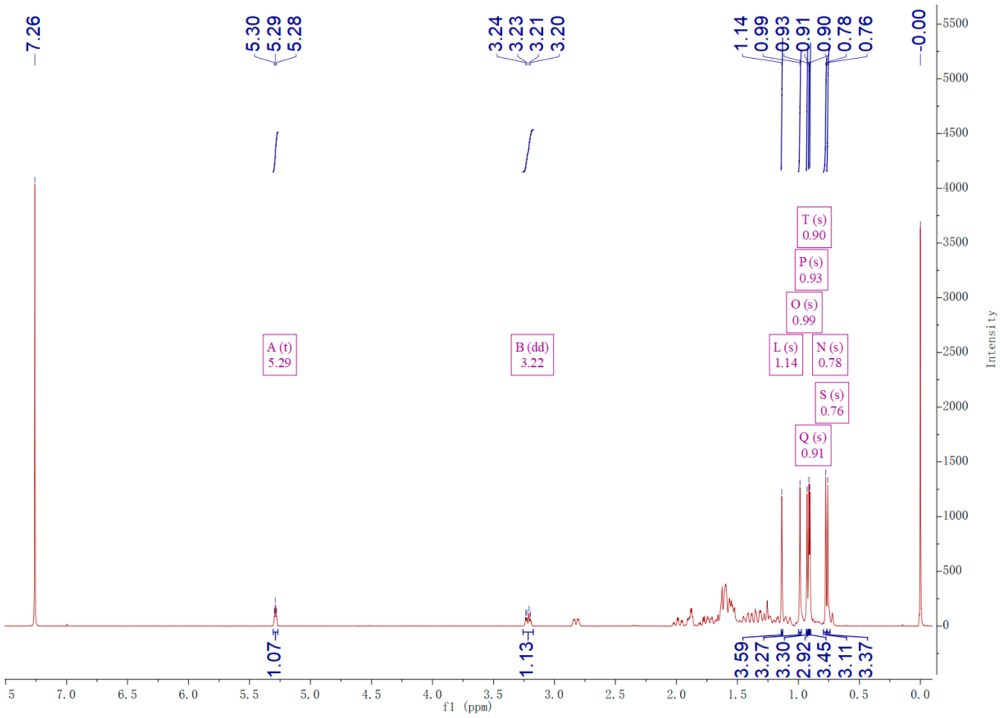

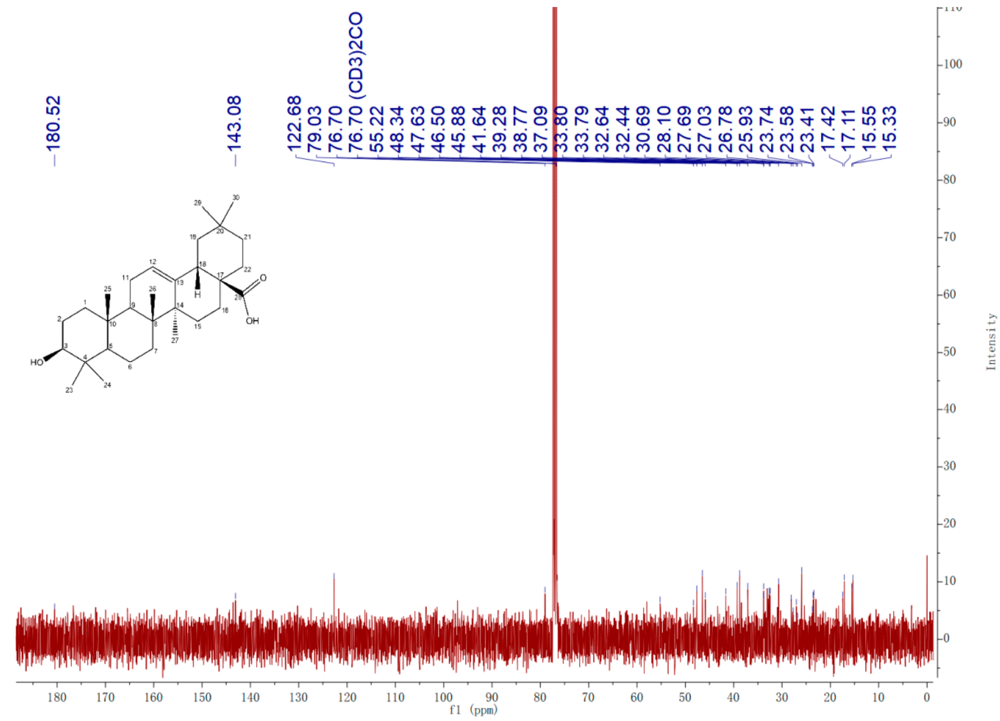

Figure S14. ^{1}H-NMR (top) and ^{13}C-NMR (bottom) spectra of Oleanolic acid, a compound from *Euphorbia uralensis*

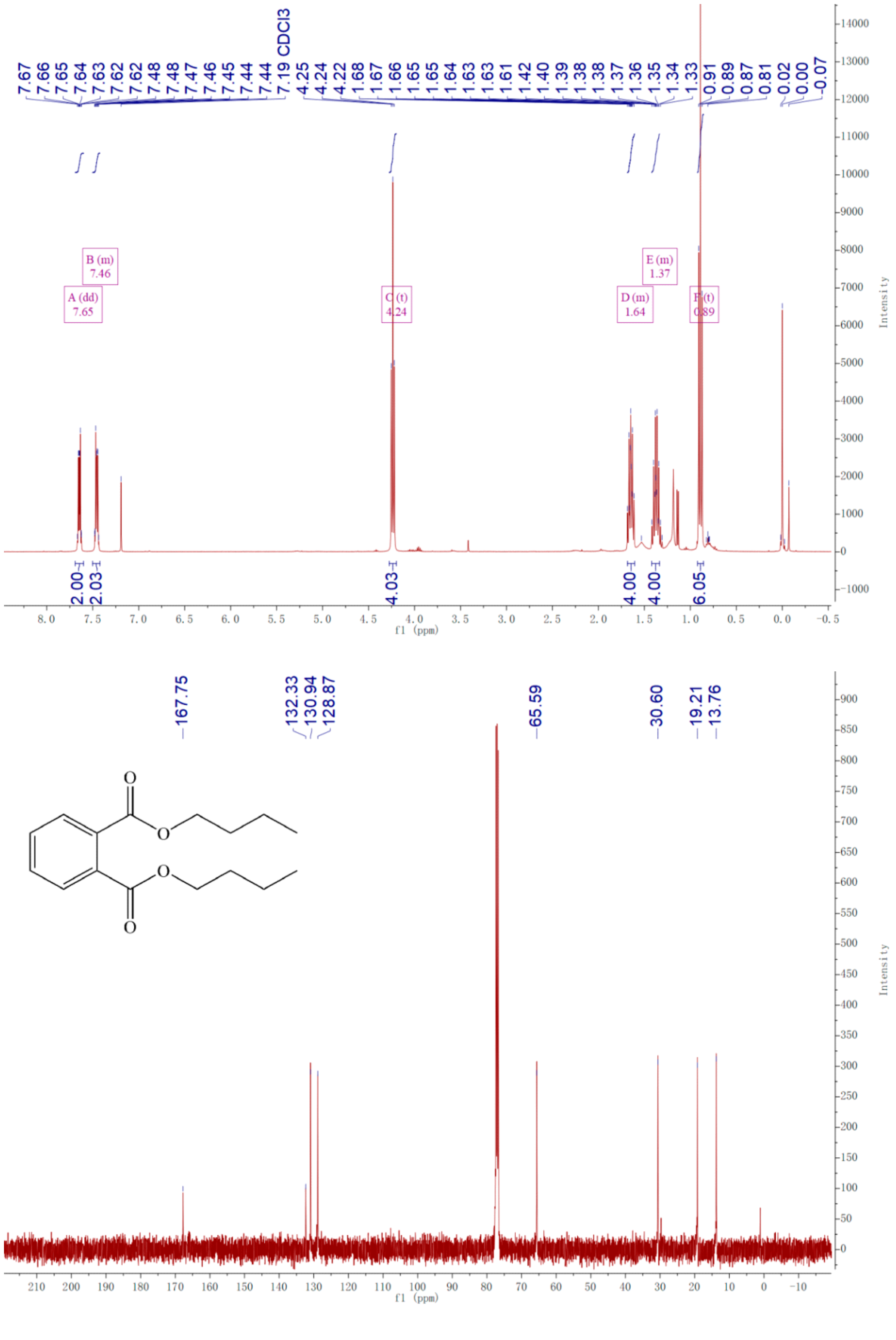

Figure S15. ^{1}H-NMR (top) and ^{13}C-NMR (bottom) spectra of Dibutyl phthalate, a compound from *Euphorbia uralensis*

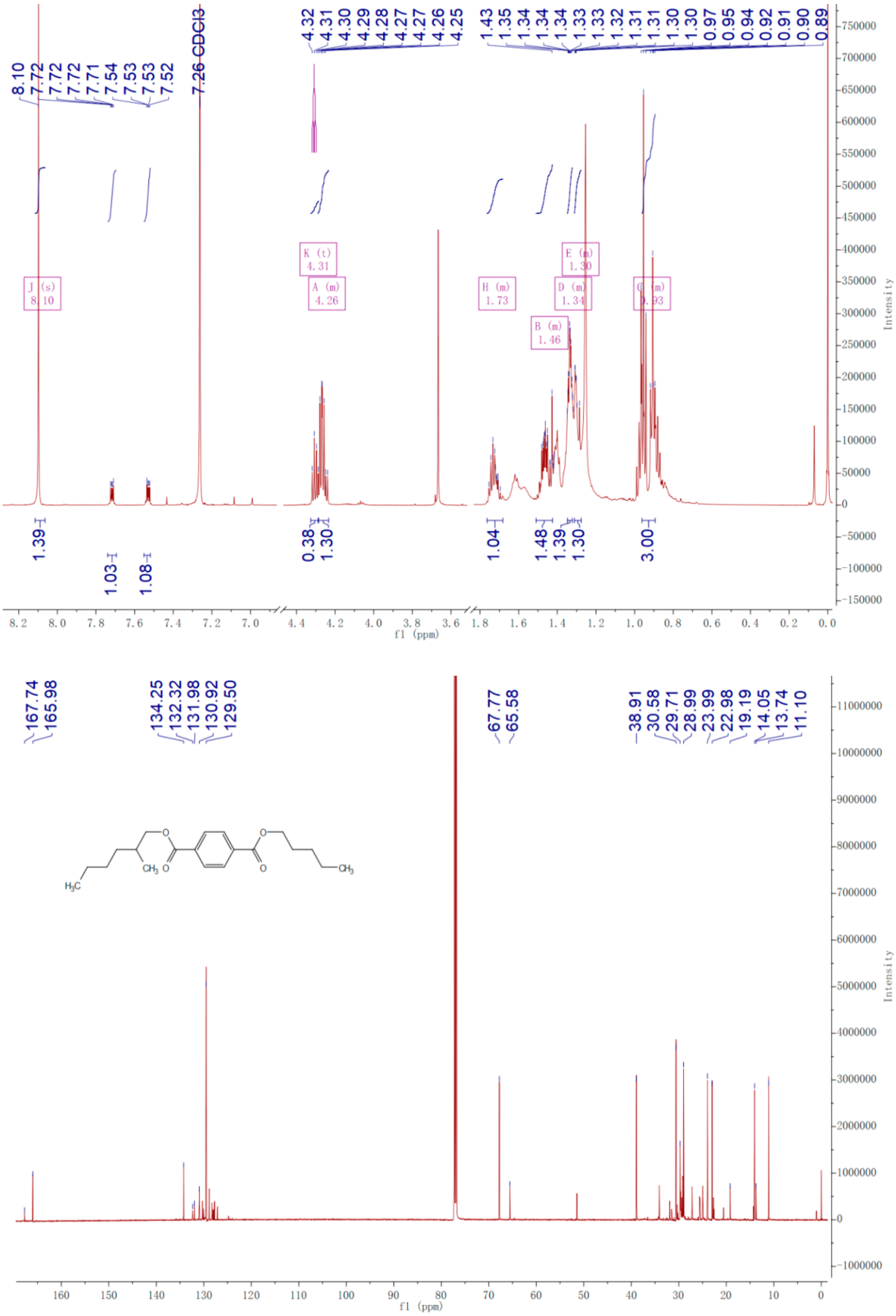

Figure S16. ^{1}H-NMR (top) and ^{13}C-NMR (bottom) spectra of Auriculatum A, a compound from *Euphorbia uralensis*

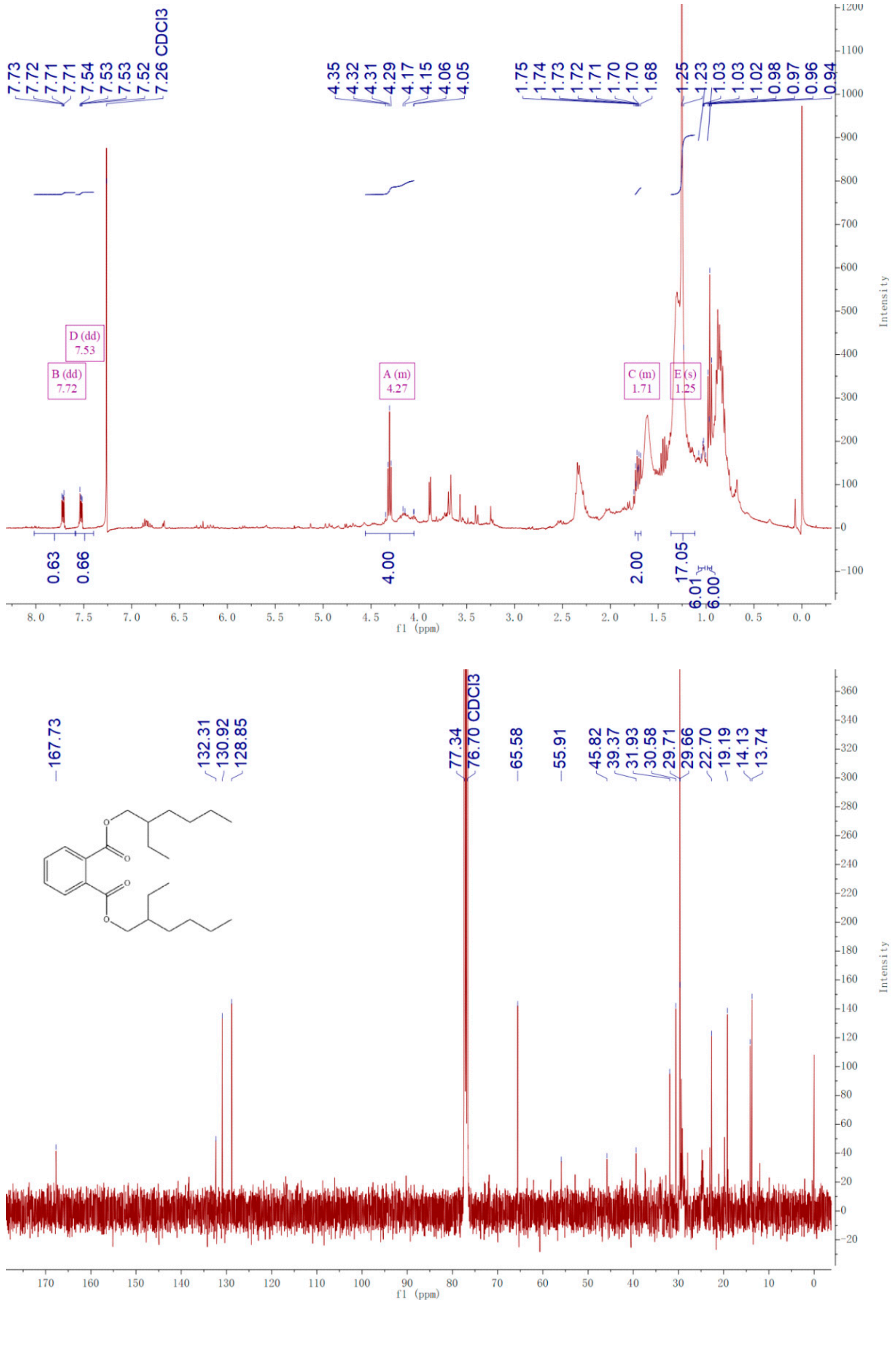

Figure S17. ^{1}H-NMR (top) and ^{13}C-NMR (bottom) spectra of di-(2-ethyl)hexyl phthalate, a compound from Euphorbia uralensis

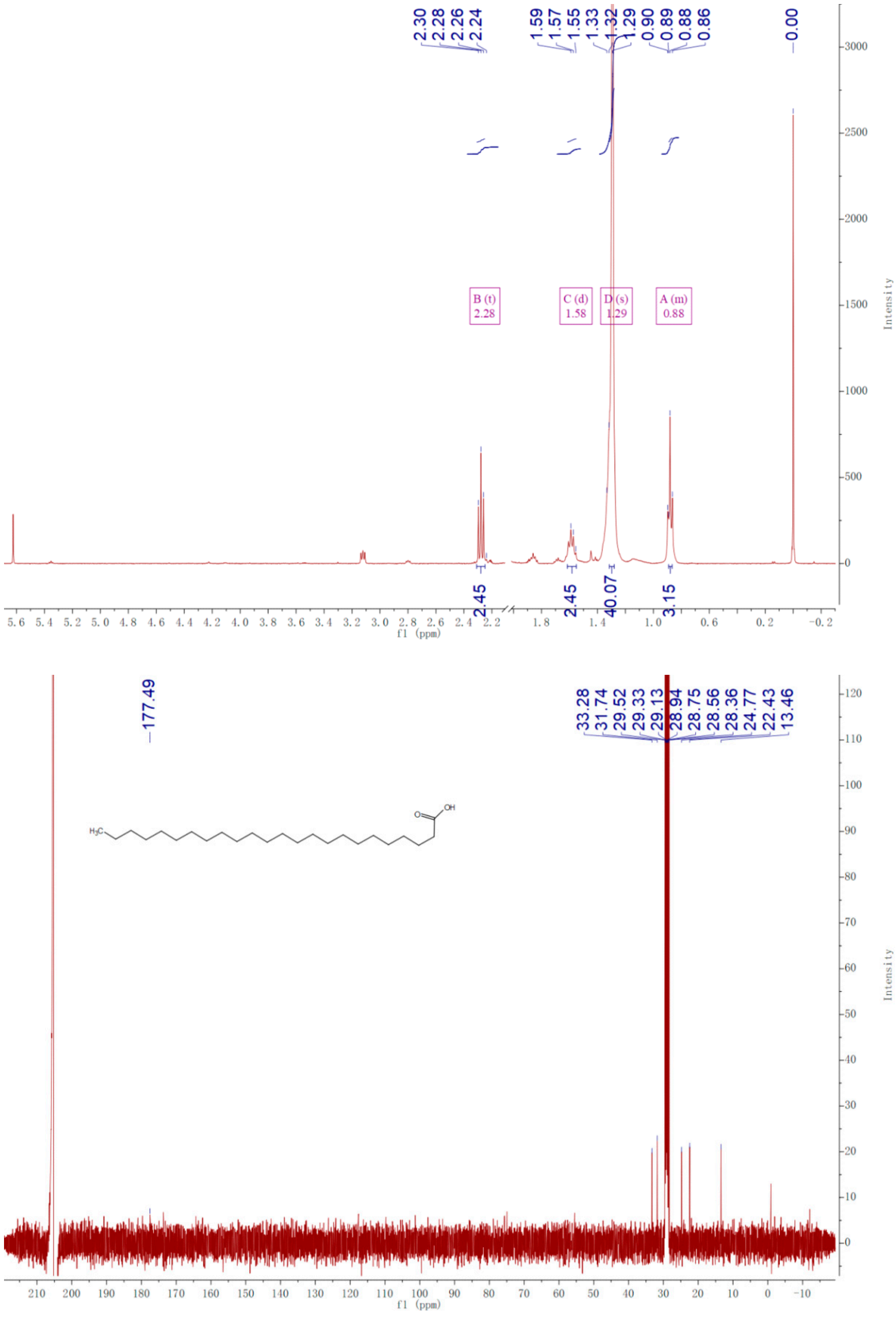

Figure S18. ^{1}H-NMR (top) and ^{13}C-NMR (bottom) spectra of n-tetradecanoic acid, a compound from *Euphorbia uralensis*

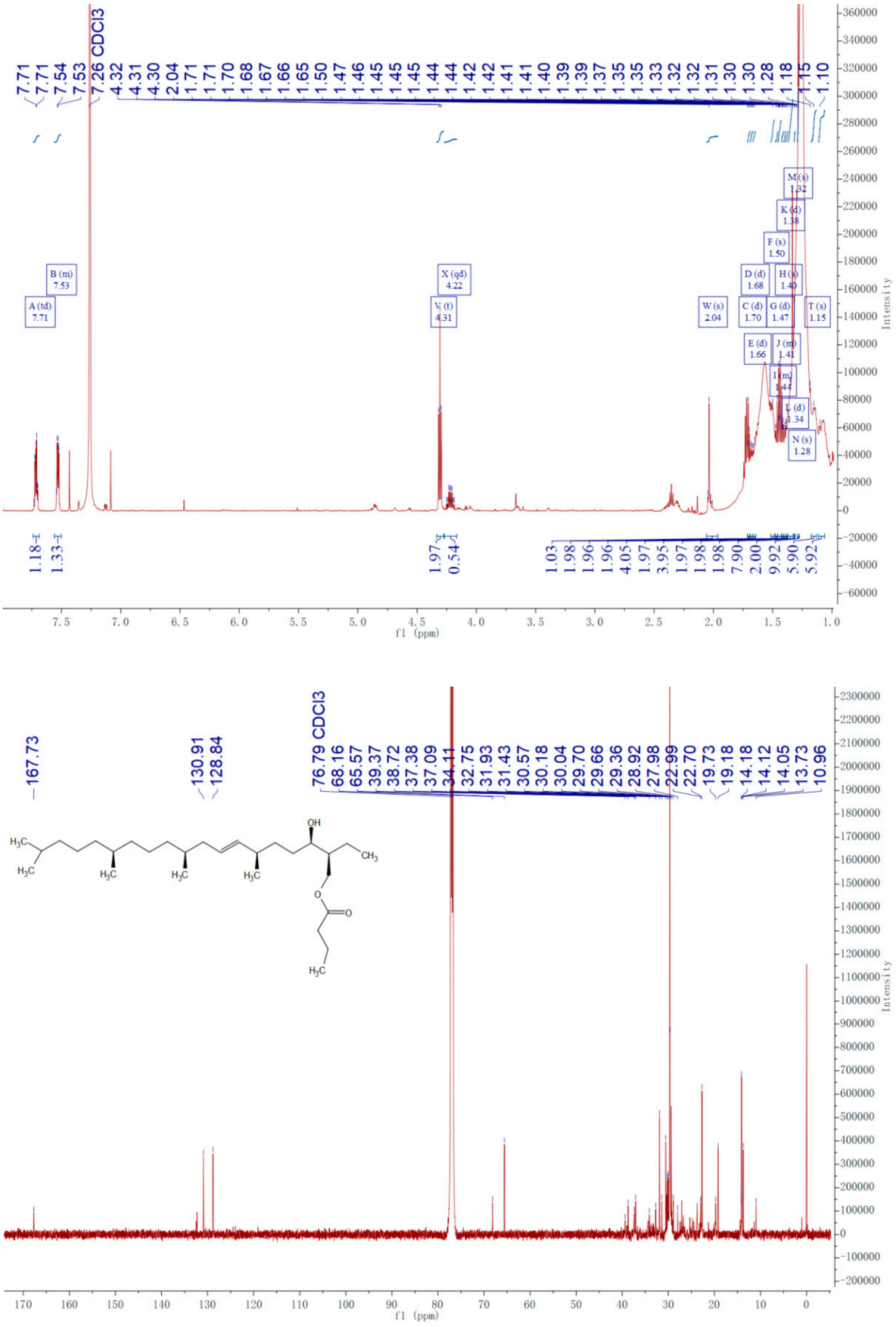

Figure S19. ^{1}H-NMR (top) and ^{13}C-NMR (bottom) spectra of 2,6,10,14-tetramet hyl-18-butylcarboxymethyl-12-en-17*β*-ol, a compound from *Euphorbia uralensis*

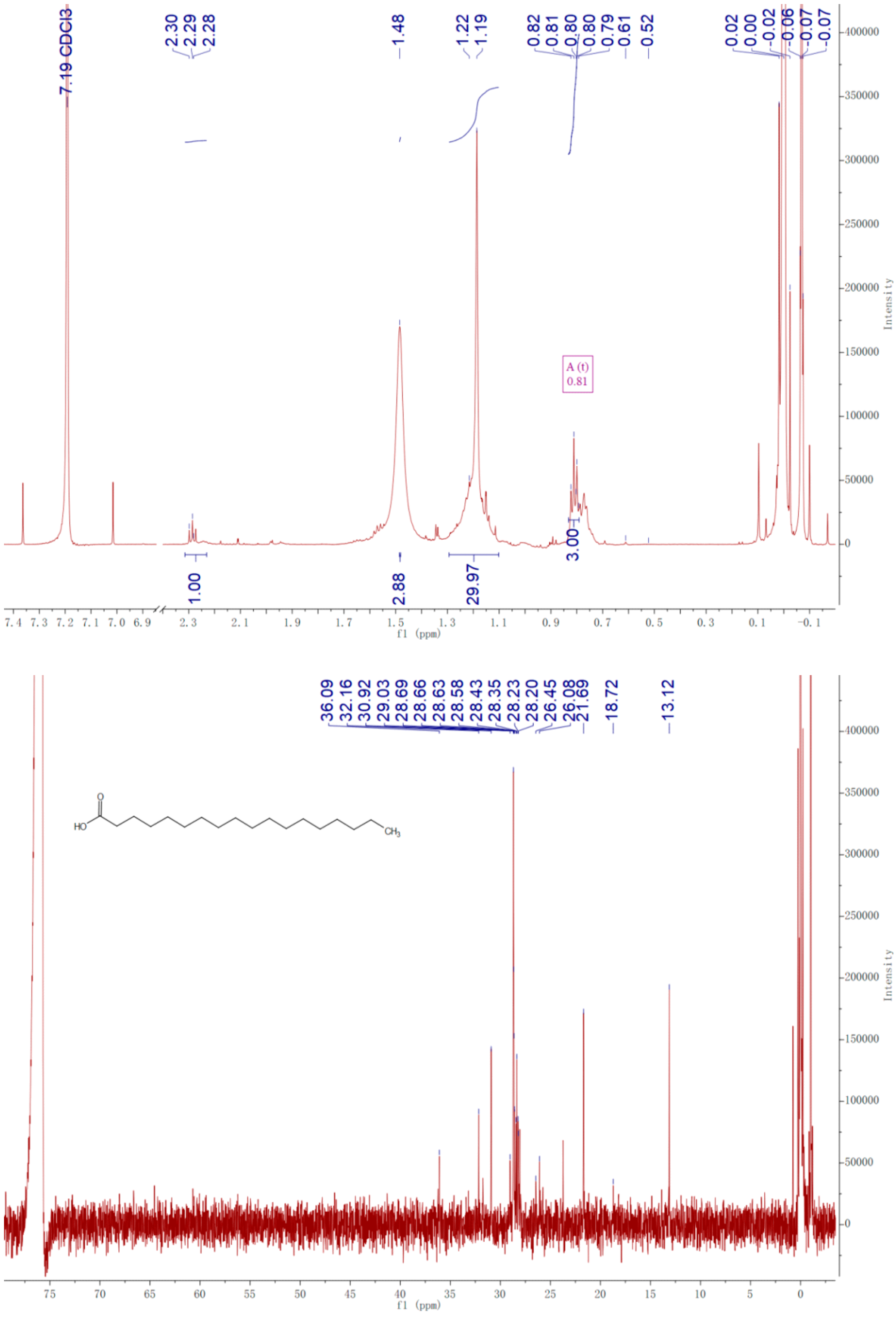

Figure S20. ^{1}H-NMR (top) and ^{13}C-NMR (bottom) spectra of n-octadecanoic acid, a compound from *Euphorbia uralensis*

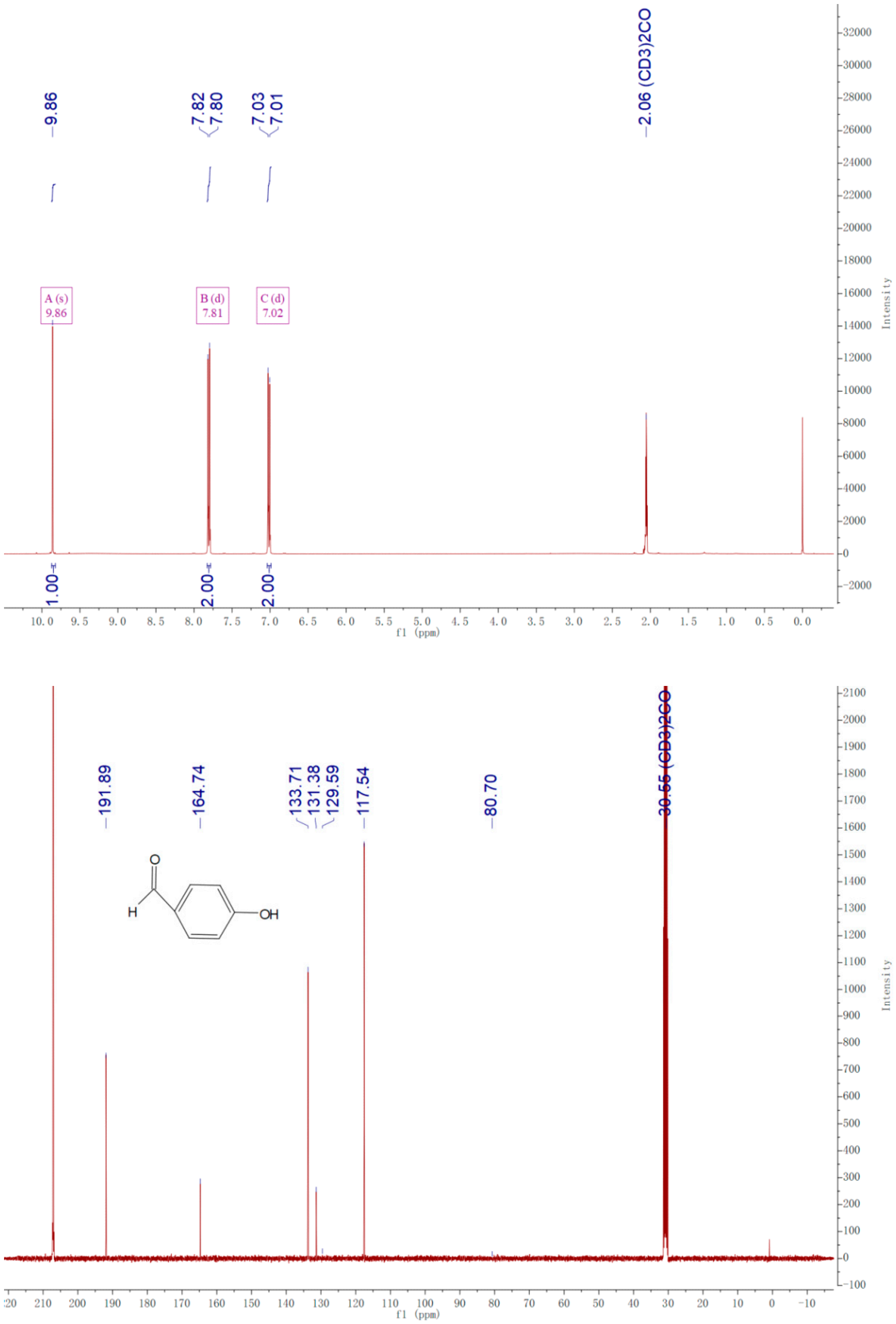

Figure S21. ^{1}H-NMR (top) and ^{13}C-NMR (bottom) spectra of p-hydroxybenzaldehyde, a compound from *Euphorbia uralensis*

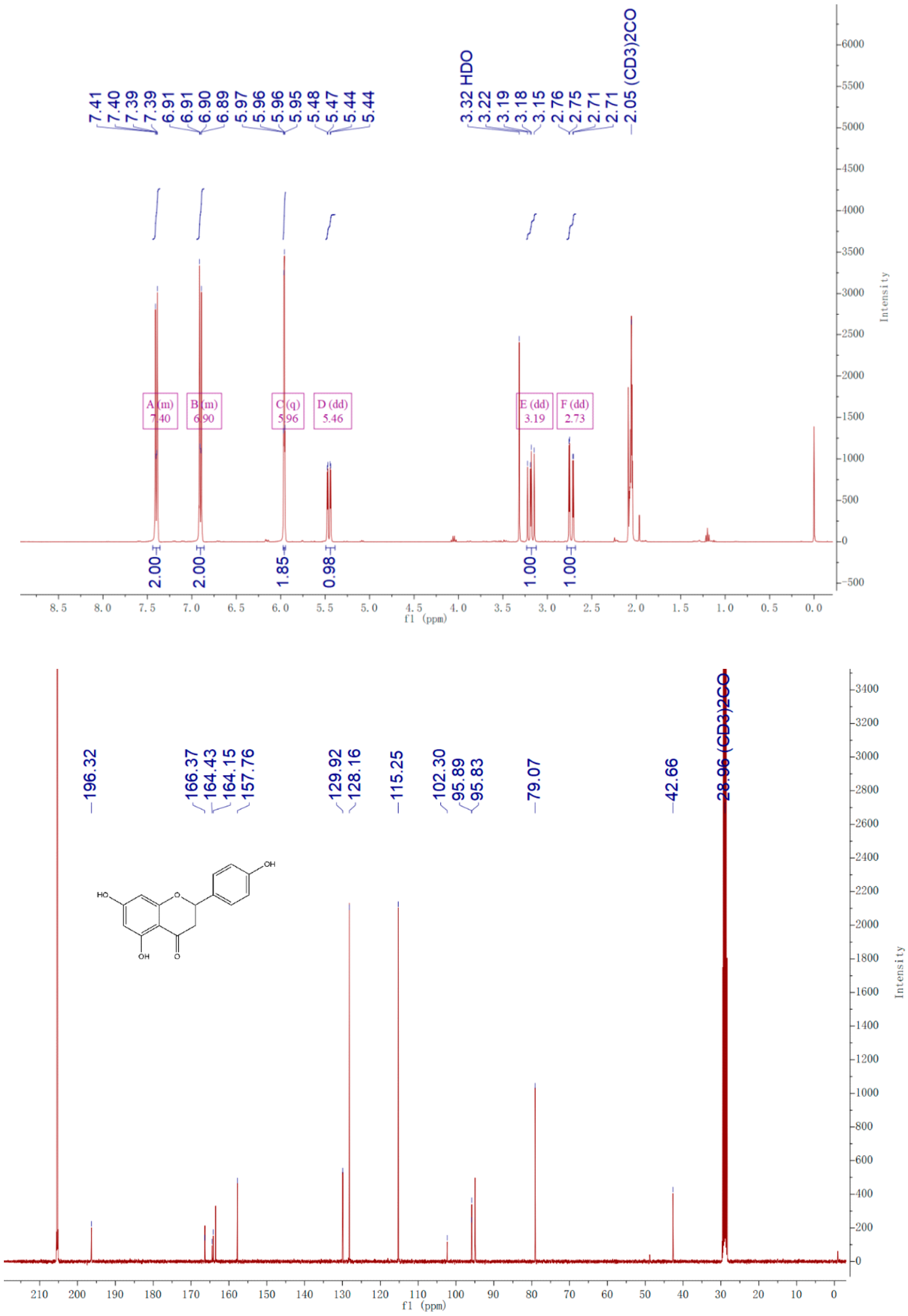

Figure S22. ^{1}H-NMR (top) and ^{13}C-NMR (bottom) spectra of naringenin, a compound from *Euphorbia uralensis*

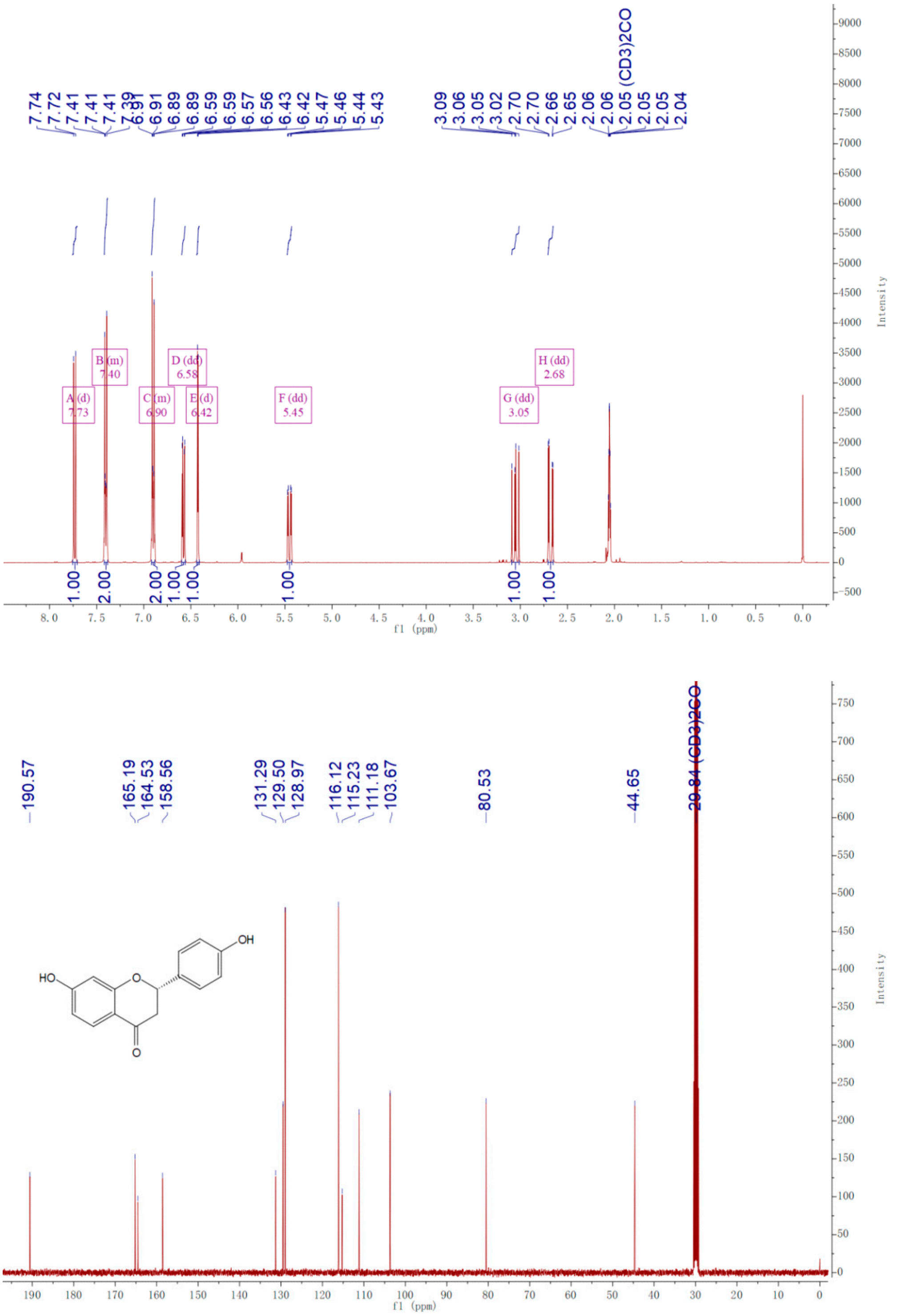

Figure S23. ^{1}H-NMR (top) and ^{13}C-NMR (bottom) spectra of glycyrrhetinic acid, a compound from *Euphorbia uralensis*

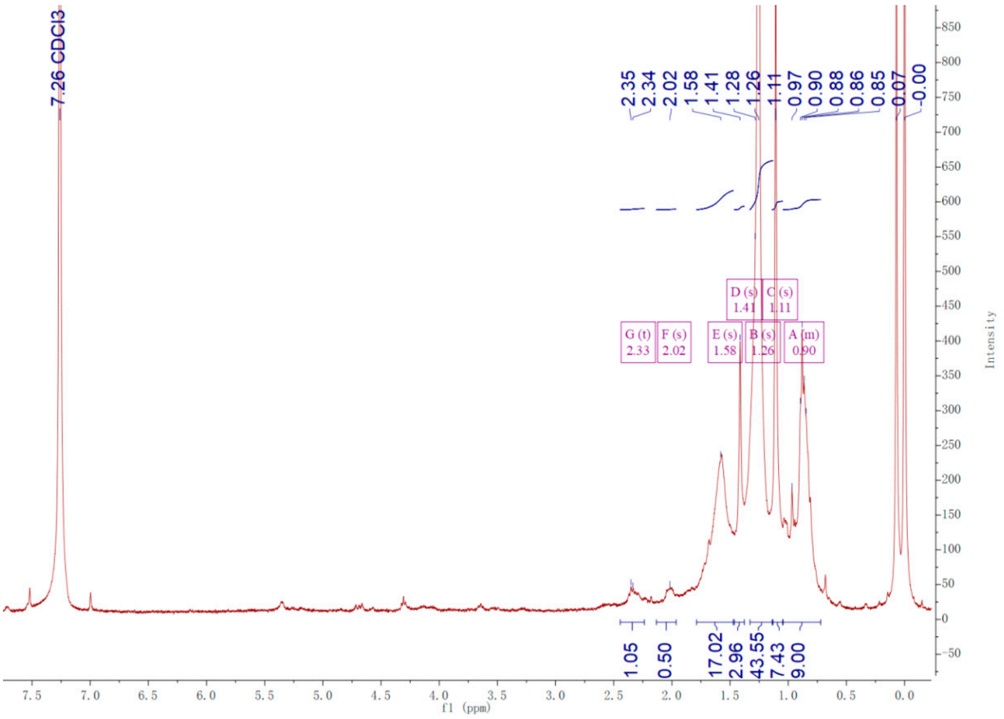

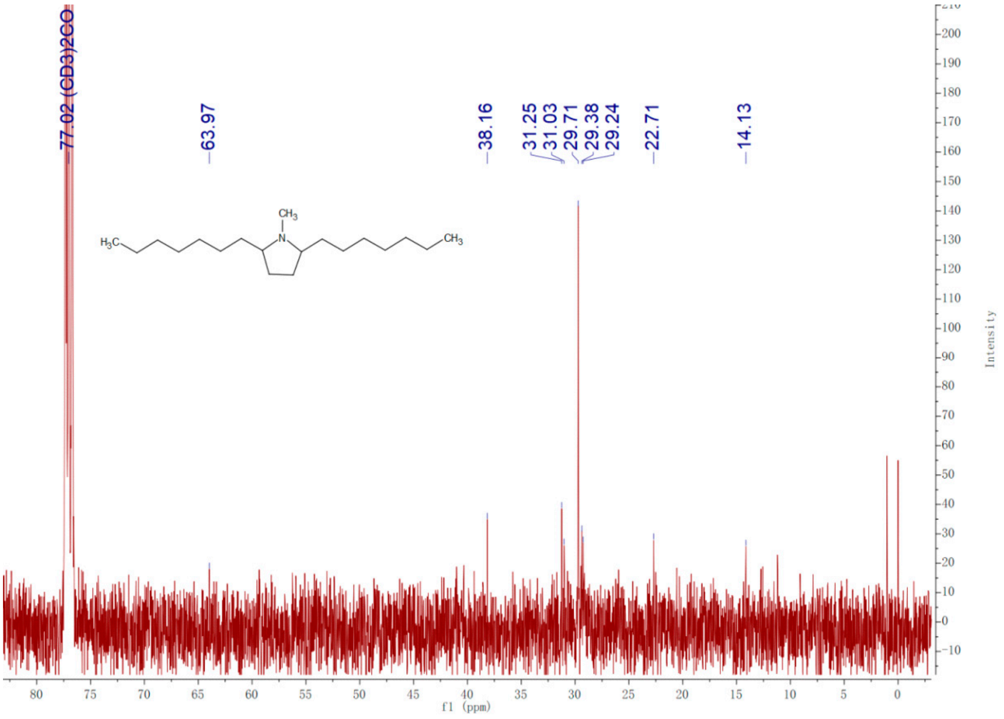

Figure S24. ^{1}H-NMR (top) and ^{13}C-NMR (bottom) spectra of punigratine, a compound from *Euphorbia uralensis*

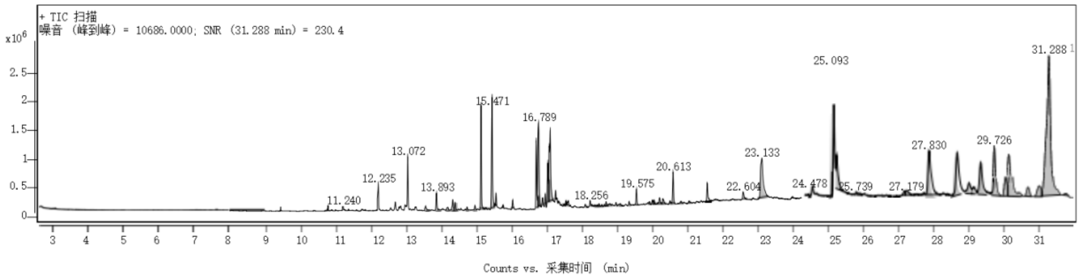

Figure S25. GC-MS total ion chromatogram of petroleum ether fraction of *Euphorbia uralensis*.

A

Index	Donor	Acceptor	Occupancy%
1	50TYR(chain_A#OH)	Ligand(18239#O)	**0.2%**
2	242LYS(chain_B#NZ)	Ligand(18236#O)	**0.6%**
3	242LYS(chain_B#NZ)	Ligand(18237#O)	**0.2%**
4	249LYS(chain_B#NZ)	Ligand(18239#O)	**0.1%**
5	276ARG(chain_B#NH1)	Ligand(18238#O)	**0.2%**
6	280ASN(chain_B#ND2)	Ligand(18239#O)	**0.7%**
7	1168LYS(chain_C#NZ)	Ligand(18207#N)	**0.3%**
8	1168LYS(chain_C#NZ)	Ligand(18236#O)	**0.3%**
9	1171ASP(chain_C#N)	Ligand(18237#O)	**0.1%**
10	1175GLN(chain_C#NE2)	Ligand(18236#O)	**0.8%**
11	1175GLN(chain_C#NE2)	Ligand(18237#O)	**0.1%**

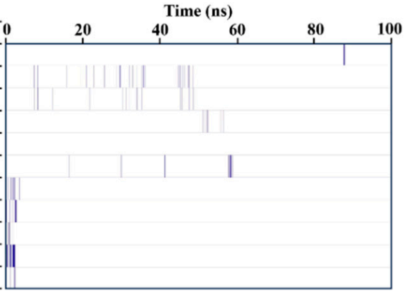

B

Index	Donor	Acceptor	Occupancy%
1	404ARG(chain_B#NH1)	Ligand(18207#N)	**1.4%**

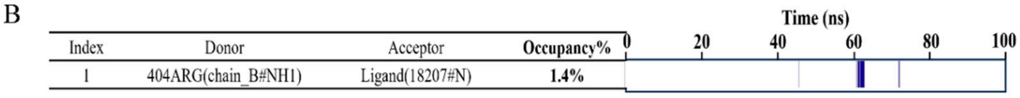

Figure S26. Hydrogen bonding frequencies between small molecules and proteins; A: Verapamil vs ABCB1; B: EUD-17 vs ABCB1.
